# Total Synthesis of Terpenes and Their Biological Significance: A Critical Review

**DOI:** 10.3390/ph15111392

**Published:** 2022-11-11

**Authors:** Aqsa Kanwal, Muhammad Bilal, Nasir Rasool, Muhammad Zubair, Syed Adnan Ali Shah, Zainul Amiruddin Zakaria

**Affiliations:** 1 Department of Chemistry, Government College University, Faisalabad 38000, Pakistan; 2 School of Chemistry and Chemical Engineering, Shandong University, Jinan 250100, China; 3 Faculty of Pharmacy, Universiti Teknologi MARA Cawangan Selangor Kampus Puncak Alam, Bandar Puncak Alam 42300, Selangor, Malaysia; 4 Atta-ur-Rahman Institute for Natural Product Discovery (AuRIns), Universiti Teknologi MARA Cawangan Selangor Kampus Puncak Alam, Bandar Puncak Alam 42300, Selangor, Malaysia; 5 Borneo Research on Algesia, Inflammation and Neurodegeneration (BRAIN) Group, Faculty of Medicine and Health Sciences, Sabah Universiti Malaysia, Jalan UMS, Kota Kinabalu 88400, Sabah, Malaysia

**Keywords:** terpenes, natural product, total synthesis

## Abstract

Terpenes are a group of natural products made up of molecules with the formula (C_5_H_8_)_n_ that are typically found in plants. They are widely employed in the medicinal, flavor, and fragrance industries. The total synthesis of terpenes as well as their origin and biological potential are discussed in this review.

## 1. Introduction

Terpenoids belong to the biogenic volatile organic compounds (BVOCs) that constitute a large and diverse class of secondary plant metabolites, with approximately 55,000 different structures [1,2,3]. The main role of these compounds is to protect plants from herbivores and pathogenic microorganisms by displaying direct toxicity, repelling herbivores, or attracting herbivores’ enemies [4,5,6,7]. The structure of terpenes is based on the linkage of isoprene units (C_5_H_8_) such as dimethylallyl pyrophosphate and isopentenyl pyrophosphate [8,9]. These two C5 building blocks generate various terpene compounds by head-to-tail condensation [10]. Depending on the number of linked isoprene units, the resulting terpenes are classified into hemi-, mono-, sesqui-, di-, sester-, tri-, sester-, and polyterpenes (C5, C10, C15, C20, C25, and longer chains of C5, respectively) [11]. Terpenes are hydrocarbons, while terpenes containing additional functional groups, usually containing oxygen, are called terpenoids [12].

In recent years, small subgroups of novel terpenes and terpenoids have been isolated or synthesized, providing more potentially chemotherapeutic terpene compounds for clinical trials [13]. Various terpenes and terpenoids, mainly monoterpenes and sesquiterpenes, can be continuously emitted from specialized storage organs in leaves, stems, and trunks of trees, while others are synthesized de novo after invasion of a pathogen to defend the plants [14,15,16,17]. Many volatile terpenoids such as menthol and perillyl alcohol are used as raw materials for spices, flavorings, fuels, and cosmetics. They are also used as pesticides, industrial raw materials, etc., such as pyrethrin and limonoids, which are often used as insecticides [18].

As deduced from their various structures, terpenes and terpenoids have been reported to exhibit diverse biological activities. Among them, the beneficial effects of terpene and terpenoid compounds on human health have been attracting the attention of numerous researchers, and their roles in various human disease processes, such as inflammatory diseases, tumorigenesis, and neurodegeneration, have been studied using cell and animal models for many decades, suggesting terpenes and terpenoids as potential chemopreventive and therapeutic agents for various diseases [19,20,21].

This review summarizes the total synthesis of significant terpenoid derivatives and their origins in order to meet the needs of drug research and development.

## 2. Hemiterpene

Hemiterpenes are the simplest among the terpenes. The most prominent hemiterpene, isoprene, is extracted from the leaves of conifers, poplars, willows and oaks, and *Hamamelis japonica* (herbs) [22,23]. One of the isoprene derivatives, bromo-3-methyl-2-butene, ordinarily known as prenyl bromide, can be utilized in place of a prenyl substrate in a variety of inorganic and organic compound syntheses [24]. Hemiterpene derivatives identified as hemiterpene glycoside were extracted from several floras such as *Spiraea prunifolia* [25,26], *Megoura crassicauda* [27], *Securidaca inappendiculata* [28,29], *Ilex rotunda* [30], *Ilex pubescens* [31,32], and *Spiraea canescens* [33]. They are mostly used in the treatment of autoimmune diseases such as rheumatoid stiffness (RA) and inflammation [34].

**Scheme 1:**
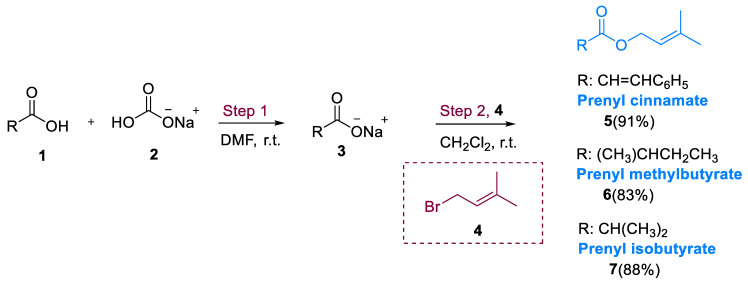
Synthesis of bromo-methyl-butene derivatives.

Hemiterpenes synthesis was accomplished via two steps. The main stage elaborates an acid–base reaction amongst carboxylic acid (**1**) and sodium bicarbonate (**2**) in DMF leading to the synthesis of RCO_2_Na and H_2_CO_3_ as byproducts. Secondly, there was a nucleophilic reaction between prenyl bromide (**4**) and sodium carboxylate (**3**). This was found in three hemiterpenes: prenyl cinnamate (**5**), prenyl methyl butyrate (**6**), and prenyl isobutyrate (**7**) (Scheme 1) [35].

## 3. Monoterpenes

### 3.1. Menthol

Menthol (**15**) is a main volatile oil component of a small number of aromatic plants that have been shown to have antibacterial, anti-inflammatory, and anticancer properties [36]. These plants are also utilized as insect repellents and fumigants [37]. Menthol (**15**), neomenthol (**17**), isomenthol (**16**), and neoisomenthol (**18**) are some of the eight chiral centers forms known as “menthols.” *Mentha piperita* and *Mentha arvensis* are the most common plants from which menthol (**15**) is extracted.

A metal–acid (bifunctional) catalyst is used to transform citral (**9**) into menthol (**15**). Citral (**9**) is hydrogenated to citronellal (**8**), citronellal is cyclized to isopulegols (**11–4**), and isopulegols (**11–14**) are hydrogenated to menthols (**15–18**). In this pathway, C=C hydrogenation and cyclization reactions are often stimulated by the metal and acid sites of catalysts. During the hydrogenation, dihydro citronellal (**10**), and 3,7-dimethyl-1-octanol (**19**) are produced, whereas citronellal and citronellol hydrogenation yields are chief perfumery compounds.

**Scheme 2:**
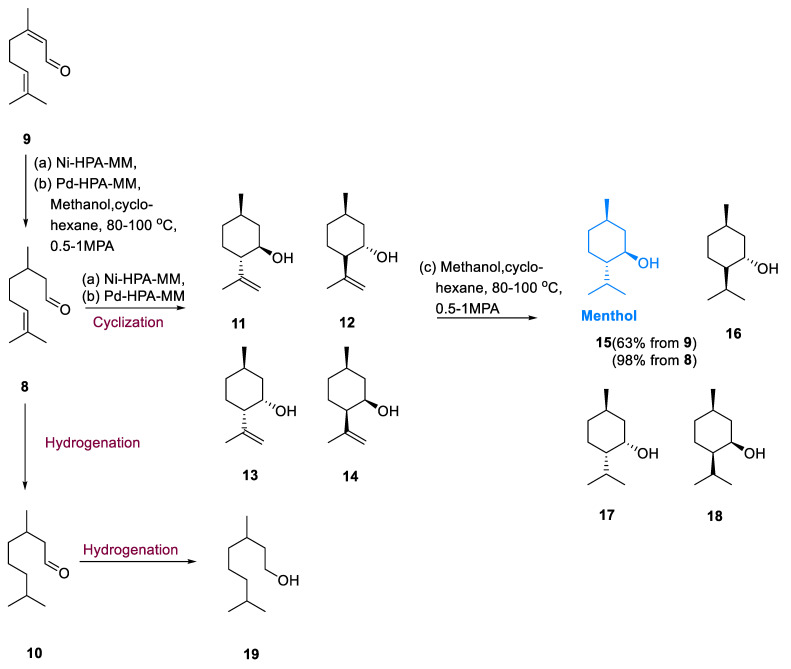
Total synthesis of menthol.

Pd as well as Ni precursors are filled with phosphoglutamic acid and acidic support (HPA-MM). For the synthesis of menthol, these catalysts have been used in citral and citronellal hydrogenations. The conversion of citral (**9**) to menthols (**15–18**) was catalyzed by a stable proportion of metallic and acidic positions, which increased selectivity. The strongest menthol selectivity was achieved by medium Bronsted and robust Lewis acidic sites that were preoccupied with Ni intermediate-containing metal sites. Optimized conditions (8 wt%, P 0.5–1.0 MPa and T = 80 °C) were helpful for obtaining the highest menthol selectivity (Scheme 2) [38].

### 3.2. 9-Hydroxy-Isoegomaketone

9-hydroxy-isoegomaketones (**25**) originate from the dried leaves of *Perillae Folium* belonging to the *Perilla. frutescens* varieties *frutescens, P. frutescens var. acuta,* and *P. frutescens var. crispa* (Lamiaceae). They devour a bitter flavor and deep possession, performing on the spleen and lung canals, and are also used as an antipyretic, diuretic, purifying, and tranquilizing mediator in recent drugs of East Asia [39]. Ethnopharmacologically, the foliage of Lamiaceae was used to prevent the gastrointestinal tract from seafood poisoning and inflammatory ailments [40].

Monoterpenoids are the chief components in the requisite fat of Lamiaceae, and they have anticancer [41,42], antioxidant [43], antifungal [44,45], neuroprotective [46], angiogenesis inhibitory [47], and anti-inflammatory properties [48]. They have also displayed retarding action on LPS-induced nitric oxide formation in RAW 264.7 cells [49].

**Scheme 3:**
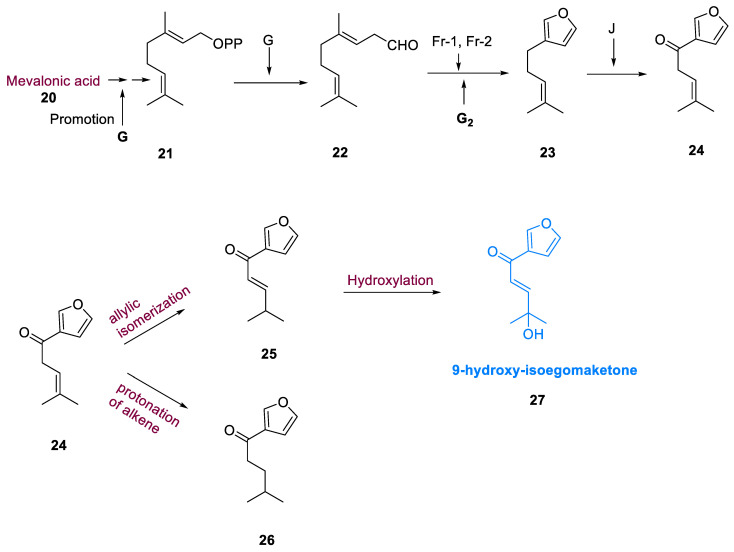
Total synthesis of 9-hydroxy-isoegomaketone.

A biosynthetic pathway for monoterpenes and phenylpropanoids was identified in Lamiaceae. The mevalonate route has been used to synthesize isoegomaketone (**25**) and perilla ketone (**26**) from GPP, geranyl diphosphate (**21**). By promoting the central gene (G), which is thought to be necessary for the instigation of synthesis of monoterpenes, GPP (**21**) generated a linear monoterpene, geranial cis-citral (**22**). Geranial (**22**) cis-citral, was transformed to perillene (**23**) via the polymeric genes (Fr1 and Fr2) regulating furan formation and gene G_2_ regulating cis-citral oxidation, and the dominant gene (J) controlled perillene oxidation at the C-6 position, increasing perillene conversion to egomaketone (**24**). Compounds (**25**) and (**26**) are prepared through allylic isomerization and proton transfer of the intermediate egomaketone’s alkene (**24**). The hydroxylation of (**25**) results in the formation of 9-hydroxy-isoegomaketone (**27**) (Scheme 3) [50].

### 3.3. Rhazinilam

The structures of monoterpene indole alkaloids are very diverse, and they have a vast variety of pharmacological activities [51]. Several anticancer drugs have come from this alkaloid subfamily. *Rhazya stricta, Kopsia* sp., and *Melodinus Australia* are anticancer alkaloids having IC_50_ values in the range of 0.6−1.2 μM that have been extracted from the deserts of the Arabian Peninsula, Iraq, and Oceania [52].

**Scheme 4:**
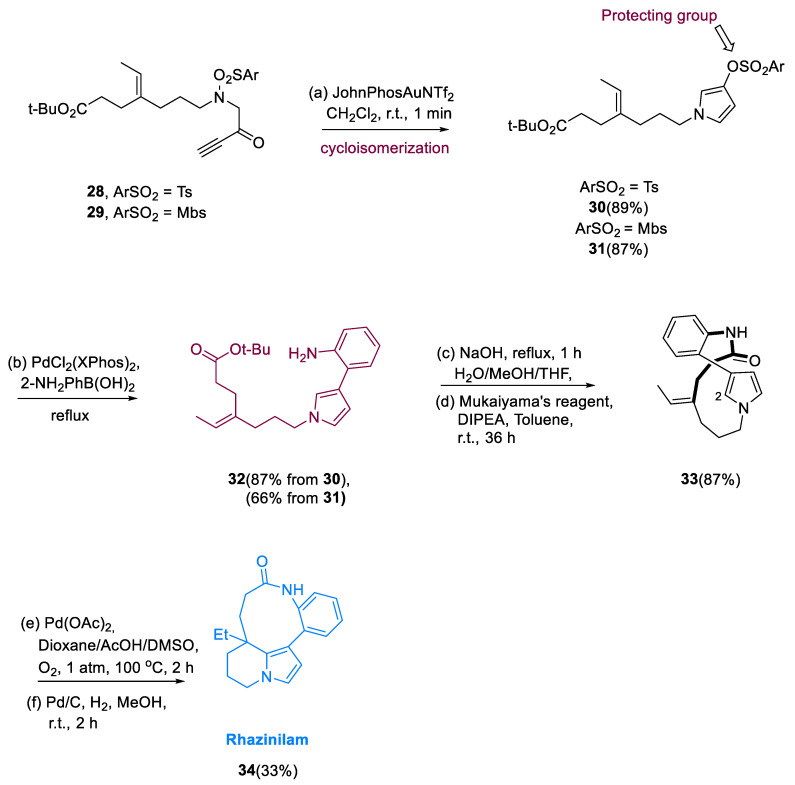
Total synthesis of rhazinilam.

*N*-ArSO_2_- or *N*-OMePhSO_2_ glycine containing methyl ester derivatives, as well as tertiary butyl having ethylidene iodoheptanoate containing moiety, were used to make two linear precursors (**28,29**) in three steps. Starting with each derivative, the gold-catalyzed cycloisomerizationsulfonyl migration cascade was carried out in the presence of JohnPhosAuNTf_2_ in dichloromethane. The *N*-alkylated pyrrolyl sulfonates have been synthesized with good efficiency by JohnPhosAuNTf_2_. The coupling of (**30,31**) with the unprotected aniline boronic acid gave the aniline pyrrole derivatives (**32**) in a good yield from tosylate (**30**) but a lower yield from (**31**). Macrolactamization, as well as intramolecular Heck addition involving oxidation, and reduction of the resultant vinyl at the terminal site, were carried out to complete the synthesis. According to Zakarian’s conditions, macrolactam synthesis was simple and achieved a good overall yield, furnishing rhazinilam (**34**), which was quantitatively produced by hydrogenation of the latter under standard conditions (Scheme 4) [53].

### 3.4. Kopsiyunnanine K

The Kopsia genus belongs to the Apocynaceae family, a chief source of alkaloids containing indole monoterpenoids having a wide range of biological activities, such as antirheumatism, anti-inflammatory and cholinergic activities, and structural diversity [54,55]. *Kopsia arborea*, a plant used for the insulation of kopsiyunnanine K (**45**), is native to the province of Yunnan, China [56].

Alkylation of δ-valerolactone (**35**) with methanolhydrolyzed ethyliodide, the primary alcohol masked with a methoxymethyl group, gives (**36**). Alkaline hydrolysis of (**36**) with chiral alcohol (**37**) generates ester derivative (**38**) from δ-valerolactone (**35**). *O*-protection of allyl alcohol derivative (**38**) with methoxymethyl undergoing chiral rearrangement via asymmetric Ireland–Claisen was performed by using Bronsted base as KHMDS (potassium hexamethyl disilazane) and (Me)_3_SiCl (TMSCl) in PhMe. The addition of methanol to the resulting carboxylic acid (**40**) gave ester (**41**) a 92% yield with 92% ee (two steps).

**Scheme 5:**
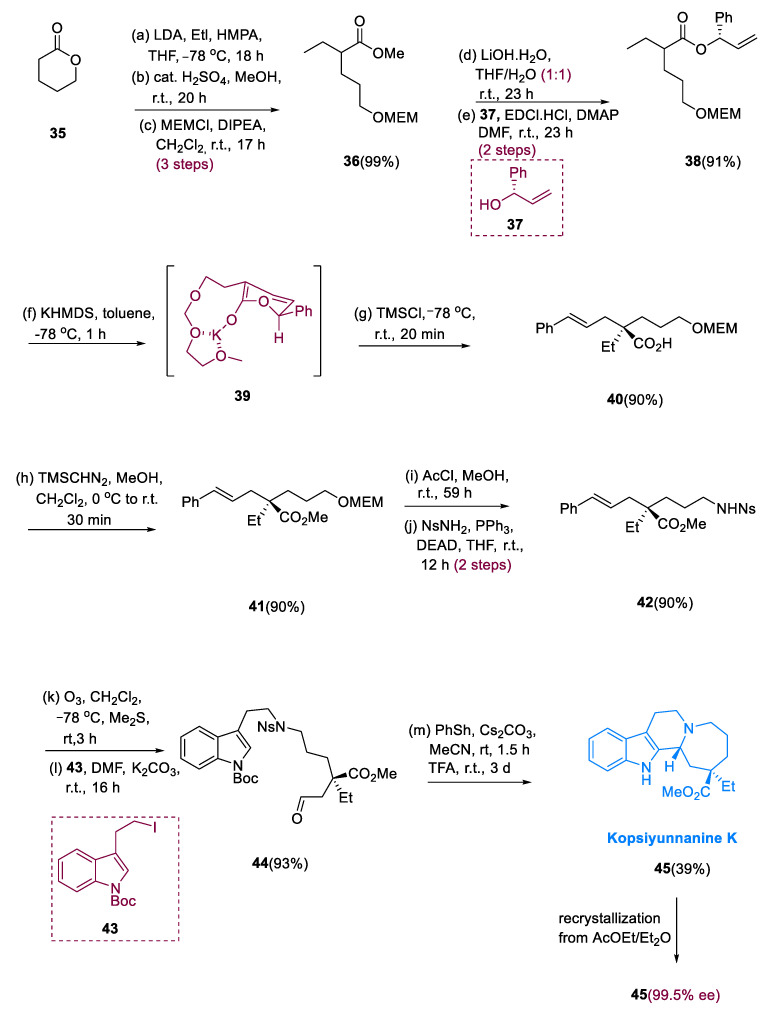
Total synthesis of kopsiyunnanine K.

The methoxymethyl (MEM) moiety was deprotected, and then the Mitsunobu reaction with NsNH_2_ was used to give an amine function, yielding amide (**42**). Alkylation of the obtained aldehyde amide with indole unit (**43**) was carried out after ozonolysis of component (**42**) to generate aldehyde (**44**). In the presence of CF_3_COOH, undergoing deprotection of the Ns group on the N_b_ position in (**44**) was followed by intramolecular Pictet–Spengler diastereoselective reaction of the amine, yielding kopsiyunnanine K (**45**) as a major diastereomer in a quantifiable quantity. After recrystallization, an optically pure product was obtained (Scheme 5) [57].

### 3.5. Pyrano [3,2-α] Containing Carbazole–Monoterpenoid Alkaloids

Carbazoles with pyrano[3,2-α] substituents are a subgroup of carbazole alkaloids found in the Rutaceae family (belonging to the genera Glycosmis, Clausena, and Murraya), having significant insect antifeedent activities and a variety of structural characteristics [58,59]. In 1964, Chakraborty et al. isolated girinimbine (**54**) from terrestrial plants [60]. From *Murraya koenigii*, Kapil’s coworkers discovered mahanimbicine 61 and bicyclomahanimbicine (**63**) in 1970.

The proper structure for bicyclomahanimbicine was proposed by Crombie and Whiting et al. [61]. Murrayamine-J (**62**), Murrayamine-M (**64**) [62], and Murrayamine-G (**65**) [63] were isolated from *Murraya euchrestifolia* by Wu et al. Chakraborty et al. isolated the carbazoles having hexacyclic alkaloid isomurrayazoline (**66**) from *Murraya koenigii* stem bark in 1982 [64].

The diarylamine (**48**) was obtained by coupling toluidine (**47**) via Buchwald–Hartwig with *m*-bromoanisole (**46**) in dicyclohexylphosphino-2′, 6′-dimethoxybiphenyl (SPhos). 

The main product of the oxidative palladium(II)-catalyzed cyclization of methoxy-containing methylcarbazole derivatives (**49**) yielded 89%. The methyl ether was cleaved to yield 2-hydroxy-6-methylcarbazole (**51**) in a 91% yield. Isogirinimbine (**53**) was obtained in a 63% yield by generating the ether having dimethylpropargyl moiety using the synthesis of Godfrey, by a Claisen rearrangement that is thermally induced, a 1,5-hydrogen shift, and closing of the electrocyclic ring. The 3-methylregioisomer of a molecule (**54**) was found as a by-product of the isogirinimbine synthesis (**53**). *N*-tosylcarbazole (**56**) was used to protect the 2-methoxy-6-methylcarbazole (**55**) at first.

The breakdown of the methyl ether was obtained via catalyzation of copper with the carbonate derivatives (**57**) and underwent thermal rearrangement yields of 52/13% of compound pyrano[2,3-β]carbazole (**59**) and pyrano [3,2-α] carbazole (**58**) in a (7.7:1) ratio. To end with, by removing the *Ts’* moiety with tetrabutylammonium fluoride in compound (**59**) at high temperatures, (±)-4-(±)-mahanimbicine **(61)** was obtained. Oxidation of (±)-4-(±)-mahanimbicine **(61)** with DDQ (2, 3-di chloro-5, 6-dicyano-1, 4-benzoquinone) gives compound (**62**) (Murrayamine J). Bicyclomahanimbicine (**63**) was produced by intramolecular [2 + 2] cycloaddition of (±)-4-(±)-mahanimbicine **(61)**. Murrayamine-M (**64**) was produced by oxidizing (**63**) with DDQ. (±)-(±)-4-mahanimbicine **(61)** was swiftly entirely transformed into murrayamine-G (**65**) in a 65% yield after being treated with CSA (camphor-10-sulfonic acid) at r.t. to 70 °C for 384 h. 

**Scheme 6:**
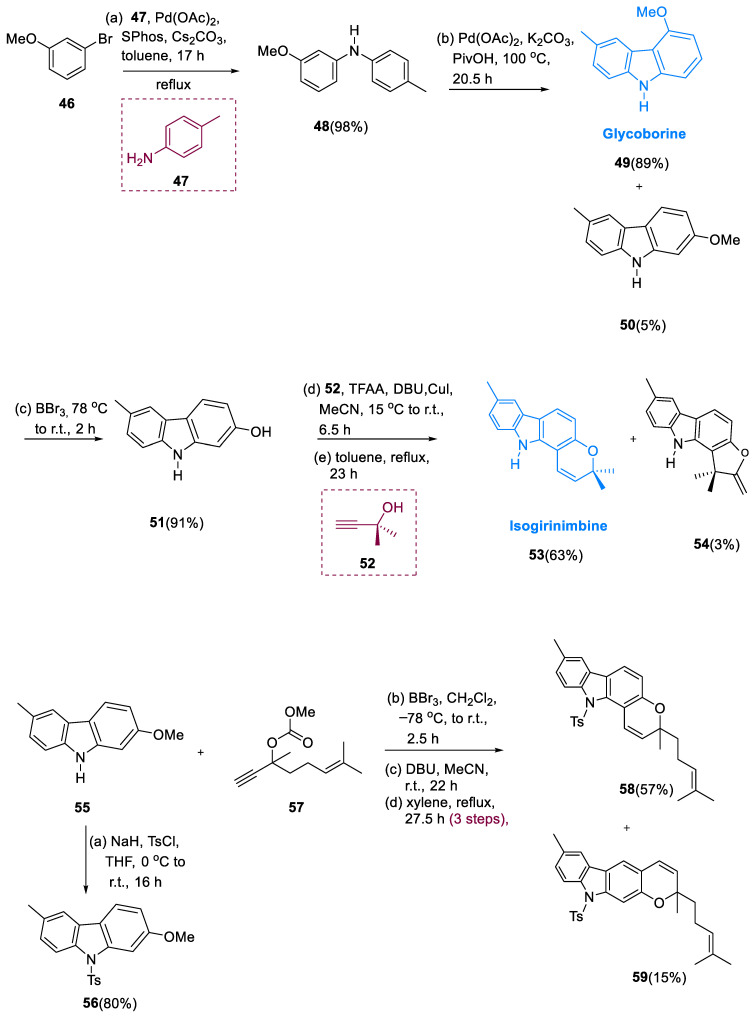

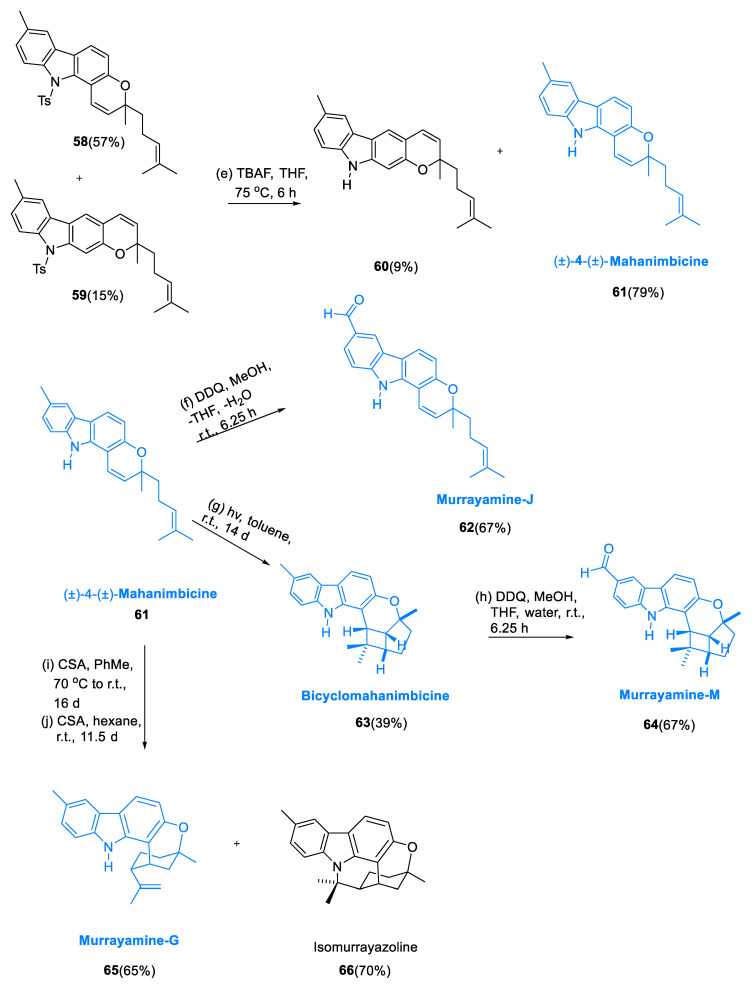
Synthesis of derivatives of pyrano [3,2-α] containing carbazole–monoterpenoid alkaloids.

Catalytic quantity of CSA in hexane at r.t. and cycloisomerization of (±)-mahanimbicine [(±)-**61**] gave a (1:1) mixture of (**65**) and (**66**) in a 65/70% yield, which was then separated by HPLC, giving pure isomurrayazoline (**66**) in a 16% yield (Scheme 6) [65].

### 3.6. Angustilodine

Indole alkaloids belonging to monoterpene, Angustilodine (**100**), isolated from the leaves of the *Alstonia angustiloba*, a Malayan species, were isolated by Kam and Choo [66]. Morita and coworkers evaluated alkaloids from the same plant in 2008 and identified alstilobanine E, an *N*-dimethyl analog of angustilodine (**99**). They were determined to have mild relaxant effectiveness against phenylephrine-induced cardiac ring contractions in thoracic rats [67].

Indole (**67**) was treated with oxalyl chloride first, then TMS(CH_2_)_2_OH (triethylamine or trimethylsilylethanol) was added to generate oxoacetate diester (**68**) in high yield. At low temperatures, two equivalents of lithium hexamethyldisilazane were used to convert compound (**68**) (indole oxoacetate derivative) to the dianion (**70**). By the addition of one equivalent of α-chloro oxime (**69**) to this dianion (**70**), the coupled product was obtained as a mixture (1.2:1) of C(15–16) diastereomers (**71, 72**) in a virtually quantifiable amount, with the oxime moiety further protected with ether having TBS group (**73**). After reducing the keto moiety of oxoacetate (**73**) with NaBH_4_, the alcohol-containing crude mixture underwent acylation with pyridine/acetic anhydride to obtain the desired acetates (**74, 75**) as stereoisomers in a 36/55% yield.

By using flash chromatography, (**75**) as a (3:1) mixture of acetate enantiomers and keto group (**74**) as a (2:1) mixture of acetates were easily obtained. In a (1:9) ratio TEA/EtOH mixture, molecule (**76**) was catalytically hydrogenated with 10% Pd/C for reduction by intermediate azafulvene (**77**). Further, acetate molecule (**75**) treated with triethylamine/ethanol, which afforded (**76**) a high yield. Thermodynamically stable diastereoisomer (**79**) was isolated in a 73% yield by treating ester (**78**) with KHMDS (potassium hexa methyldisilazide) at 78 °C, slowly heating to 0 °C, and then finally quenching in acidic media.

The CBz derivative (**80**) was the first indole to be *N*-protected. The *O*-TBS protective group was removed, resulting in oxime (**81**), which was hydrolyzed to the piperidone under acidic conditions. Using CH_2_Cl_2_/CF_3_COOH, the ester having TMS of keto acid (**83**) can be selectively extracted from the molecule (**82**). Thus, in dichloromethane, treatment of ketoacid derivatives with PPY (*4*-pyrrolidinopyridine), DIPEA (diisopropylethylamine), and *N*-propyl-2-bromo pyridinium triflate, as well as CH_3_COOH to decrease *C*-16 epimerization, resulted in the synthesis of an inextricable 97:3 ratio of the cis-azadecalin (**87**) and the transfused composite (**85**) in a 93% yield. The indole lactones (**85**), 94% of which are unprotected, and molecule (**87**) (3%), were obtained by dehydrogenation of this mixture. Intermediate (**88**) was then deprotonated with two equivalents of lithium hexamethyldisilazide, giving dianion (**89**), which was then alkylated with monomeric formaldehyde and yielded α-hydroxymethyl containing ester (**90**) as a single diastereomer. TBS ether (**91**) was used to protect the hydroxymethyl carbon of ester (**90**). Lithium borohydride has been used to reduce γ-lactone (**91**), yielding diol (**92**). IBX has been used to cleanly oxidize diol (**92**), yielding aldehyde (**93**).

**Scheme 7:**
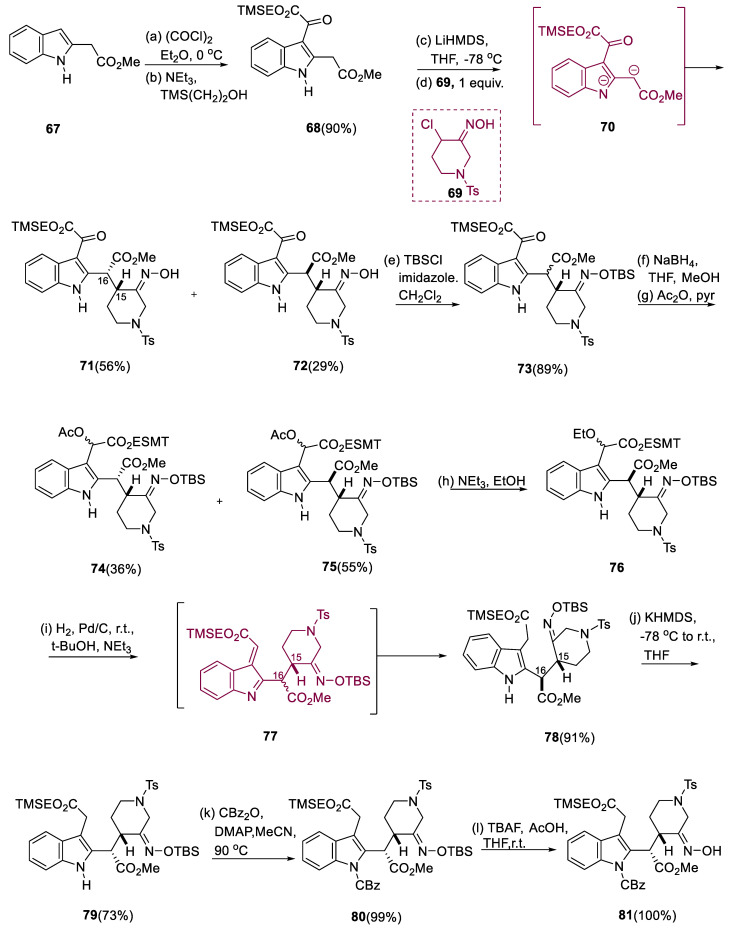

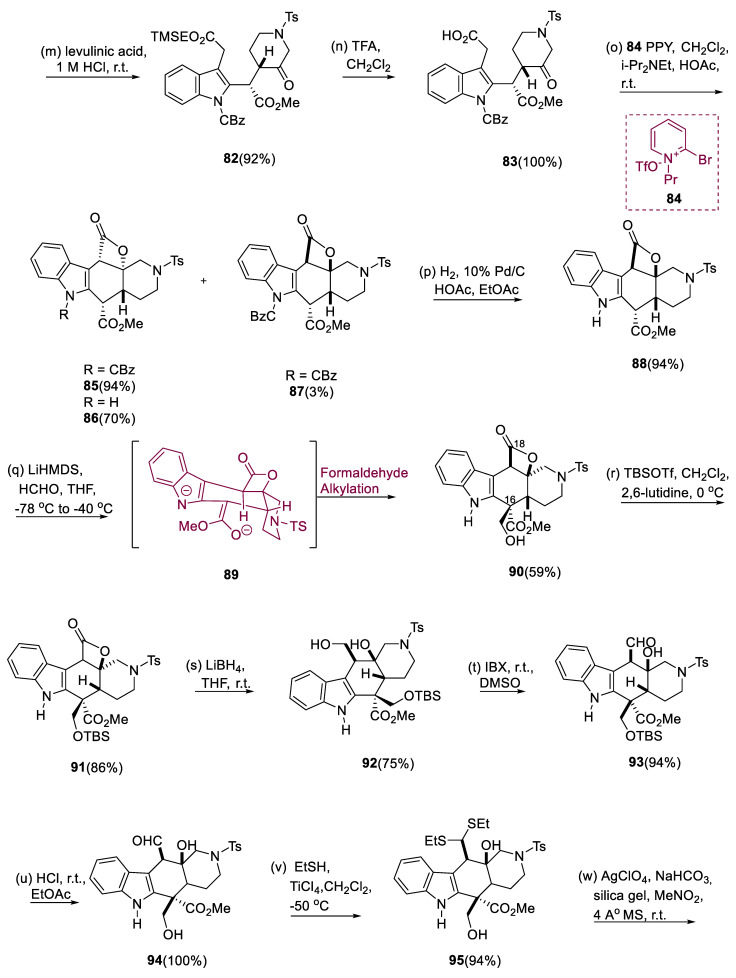

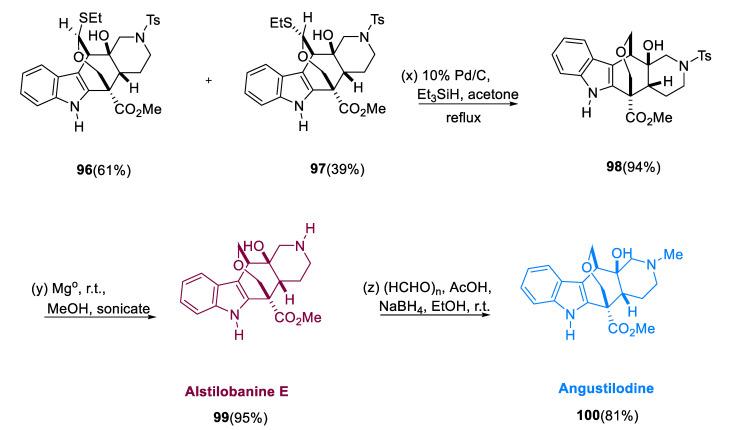
Total synthesis of (±)-angustilodine.

With ethanethiol oxidized by titanium tetrachloride, eliminating the TBS group from aldehyde (**93**) yielded compound (**94**), diol aldehyde, which then gives dithioacetal diol (**95**) in high yield. Diol (**95**) undergoes cyclization with silver perchlorate catalysis, yielding hemithioacetals as a mixture of diastereomers (**96, 97**) 3:1 in high yield. The required oxepane (**98**) was obtained in excellent yield by reducing hemithioacetals (**96**) and (**97**) with triethylsilane catalyzed by 10% palladium on charcoal. After removing the sulfonamide-protecting group of (**98**) with magnesium/methanol, racemic alstilobanine E (**99**) was obtained. By using sodium borohydride/paraformaldehyde, undergoing reductive *N*-methylation of alstilobanine E gives racemic angustilodine (**100**) (Scheme 7) [68].

### 3.7. Pleurolactone

J. K. Liu et. al. proposed the extraction of pleurolactone (**109**), a novel menthane-containing monoterpenoid, from the fungus *Pleurotus eryngii* in 2013 [69]. Pleurolactone (**109**) has four contiguous stereocenters and a hexahydrobenzofuran structure. They suppress nitric oxide synthesis in macrophages that have been stimulated by lipopolysaccharide.

**Scheme 8:**
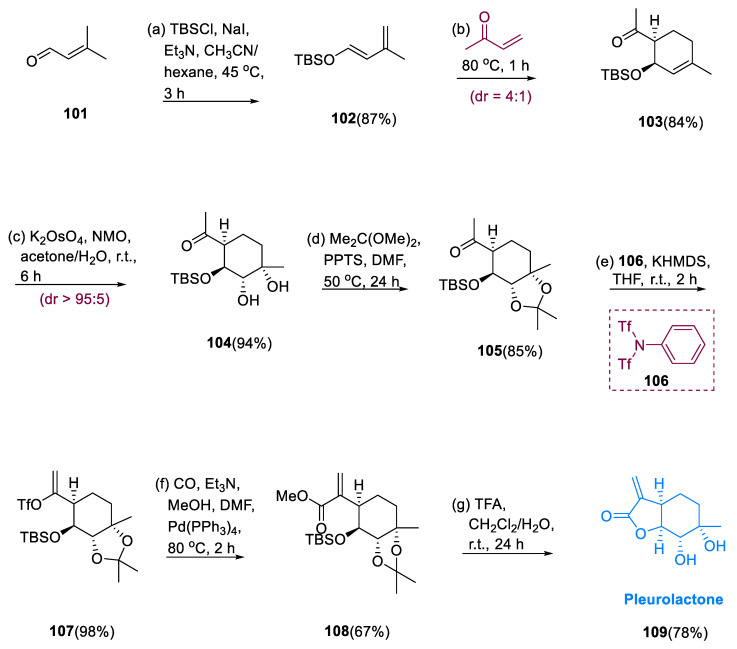
Total synthesis of pleurolactone.

The diene (**102**) was obtained in an 87% yield by treating aldehyde (**101**) with sodium iodide, tertiary butyldimethylchlorosilane, and triethylamine. Endo-adduct (**103**) was obtained in an 84% yield by the methyl vinyl ketone and a diene (**102**) undergoing a Diels–Alder reaction with (4:1) diastereoselectivity. Dihydroxylation of endo-adduct (**103**) with potassium osmate diastereoselectively yielded the required diol (**104**) as the main product in a 94% yield. Acetonide (**105**) was obtained in 85% by the protection of the dihydroxyl group with DMP (2,2-dimethoxypropane). Vinyl triflate (**107**) was obtained in 98% by treating acetonide (**105**) with potassium hexamethyldisilazide and compound (**106**) (*N*-phenyl trifluoromethane sulfonimide). The carbon monoxide insertion of triflate (**107**) with Pd(PPh_3_)_4_ in the presence of methanol yielded the methyl ester (**108**) in 67%. Then, after treating methyl ester (**108**) with trifluoroacetic acid in dichloromethane/water for 24 h at r.t., both the acetonide and TBS groups were deprotected, and the γ-lactone ring was formed simultaneously, yielding the target molecule (**109**) in a 78% yield (Scheme 8) [70].

## 4. Sesquiterpenoids

### 4.1. Abiespiroside A

Abiespiroside A (**120**), which was extracted from the Chinese fir *Abies delavayi*, has inhibitory effects against nitric oxide generation, which is a therapeutic incentive for inflammatory ailments such as arthritis [71]. Beshanzuenones D (**122**) and C (**121**) were extracted from the shed trunk of *Abies beshanzuensis*, a Chinese fir leaf. With IC_50_ values of 59.7 and 40.4 M, however, they were shown to inhibit PTP 1B (protein tyrosine phosphatase 1B), the main target for the cure of obesity and type 2 diabetes [72].

A Rubottom oxidation is used to convert (+)-carvone (**110**) to hydroxyl ketone (**111**). The reduction is extremely effective, giving the required trans-diol (**112**) in a 96% yield with a diastereomeric ratio of (5:1). Increasing the equivalents of PdCl_2_(PPh_3_)_2_ to 0.1 equivalents afforded bicyclic lactones (**113**) and (**114**) in 50% as inseparable diastereomers (2:1) in toluene at 80°C under 7 atm of CO from hydrated SnCl_2_ and PdCl_2_(PPh_3_)_2_. By converting PPh_3_ to (+)-DIOP and by introducing a DIOP (chiral bidentate ligand), the diastereoselectivity was enhanced, and the lactone products (**113,114**) were generated at 97 °C as a diastereomeric mixture (4:1) in an 81% yield. After the protection of secondary alcohol with TBS ether, the diastereomers (**113**,**115**) were recovered. The Dreading–Schmidt reaction went easily after treating lactone (**115**) with the metallozinc mixture made from MBrMA (**116**) [methyl 2-(bromo methyl) acrylate] and Zn metal, yielding the required product (**117**) in a 65% yield with diastereoselectivity (20:1) at the spirocyclic center.

**Scheme 9:**
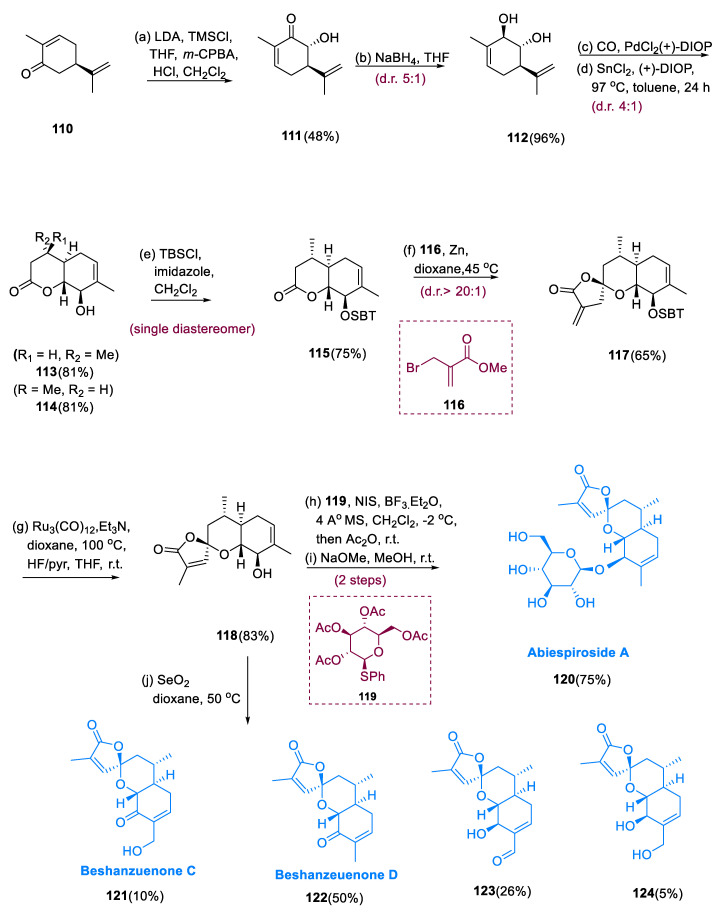
Total synthesis of abiespiroside A and their analogs.

Both Ru_3_(CO)_12_ and DBU were capable of isomerizing the α-methylene oxa-spirolactone from the exomethylene into the metastable internal alkene, which is more stable. The Ru_3_(CO)_12_ double bond was isomerized completely, and DBU only gives a maximum of 50% conversion. After separating the TBS moiety, product (**118**) was obtained in a 83% yield. β-selective glycosidation is achieved by treating molecule (**118**) with tetra-acetylated thioglycoside (**119**). Abiespiroside A (**120**) was synthesized in 75% from (**118**) by methanolysis of the acetyl groups. Treatment of (**118**) with SeO_2_ resulted in four separate oxidation molecules, including beshanzuenone D (**122**) and beshanzuenone C (**121**), which were extracted in a (50%/10%) yield, and two additional oxidation by-products were also exposed: (**123**), containing α-hydroxy aldehyde; and (**124**), containing 1,3-diol (5% yield) (Scheme 9) [73].

### 4.2. Cyperolone

Cyperolone (**139**) is a bicyclo [4.3]nonane sesquiterpene that is cis-fused. Hikino et al. were the first to isolate cyperolone (**139**) from LINNE *Cyperus rotundus* (Nutgrass Japanese) in 1966 [74]. Traditional oriental medicine has long used the rhizomes of Japanese nutgrass to treat a variety of ailments, including menstrual abnormalities and reproductive conditions [75].

Under basic parameters, (R)-(-)-carvone (**125**) is oxidized to chlorohydrin (**126**) and then degraded. Since the hydrolysis stage produced only moderate yields, we found this two-step diol (**127**) route to be superior to alternative sequences as it was scalable and yield-reliable. The use of pyridinium chlorochromate undergoes oxidative rearrangement to compound (**128**), the cyclicenone, following selective protection as the triisopropylsilyl ether of the primary hydroxyl group. The Grignard reagent, which is synthesized from propargyl bromide, was smoothly coupled with tertiary hydroxyl, giving siloxy enyne (**129**).

**Scheme 10:**
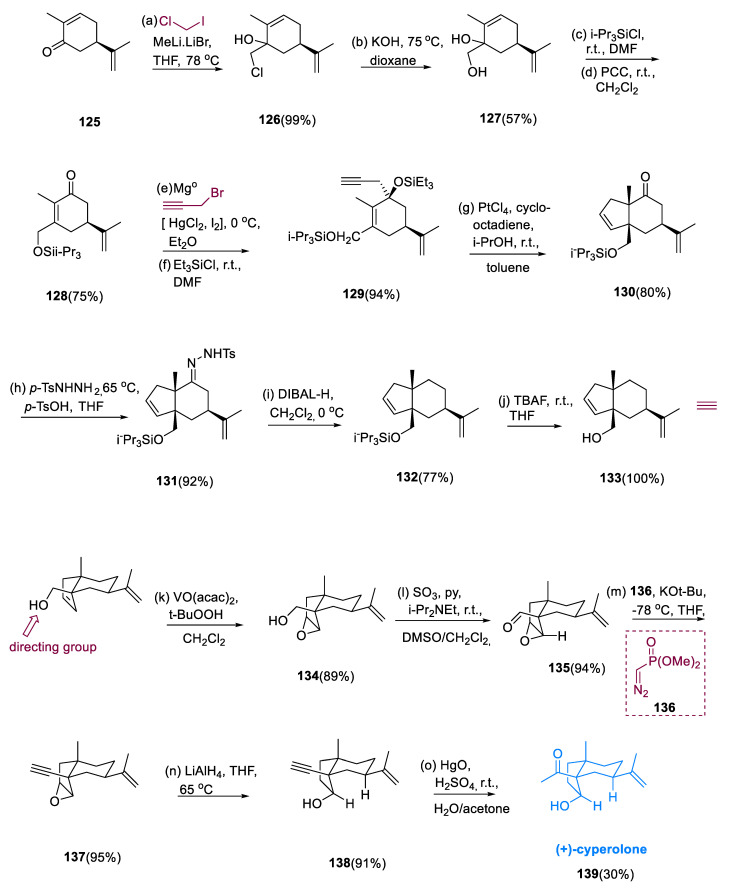
Total synthesis of (+)-cyperolone.

Only platinum catalysts were used to convert enyne (**129**) to bicycle (**130**). Both PtCl_4_ and PtCl_2_ were dynamic precatalysts in substoichiometric quantities of cyclooctadiene. The best yields were found using PtCl_4_ (20 mol%) and cyclooctadiene (80 mol%) in MePh at r.t. The carbonyl group was removed by first generating the tosyl hydrazone (**131**) and then treating it with DIBAL-H. Alcohol (**133**) was obtained by deprotecting the primary tri-isopropylsilyl ether. The homoallylic alcohol moiety was used for vanadium-catalyzed epoxidation in alcohol (**133**). Alkyne (**137**) was obtained through oxidation of the hydroxy group in a molecule (**134**) and then treated with the Seyferth–Gilbert (**136**) homologation. The secondary alcohol (**138**) was obtained in a 91% yield by nucleophilic epoxide opening with LiAlH_4_. Finally, utilizing mercuric oxide (HgO) in aq. H_2_SO_4_/acetone, terminal alkyne (**138**) was effectively transformed into (+)-cyperolone (**139**) in a 30% yield (Scheme 10) [76].

### 4.3. Illisimonin A

Illisimonin A (**150**), a sesquiterpenoid having a tricyclo[5.2.1.1,6]decane carbon structure, isolated from *Illicium simonsii* [77]. Illisimonin A in SH-SY5Y cells displayed antitumor, cytostatic, and antiproliferative activities against cell injury that is induced through oxygen–glucose deprivation.

The benzyloxymethyl ether was used to protect 2-methylcyclopenta-1, 3-dione (**140**) in its enol state. Following that, an aldol reaction with vinylogous ester having ketone (**141**) to produce tertiary alcohol (**142**) as a diastereomer. The silacycle (**143**) was created simultaneously from a diastereomeric mixture and heated up to 40 °C for 15 h. Purification and desilylation resulted in a high yield of the required Diels–Alder intermediate, racemic compound (**144**), as a single diastereomer. When the tertiary alcohol is silylated, the cyclopentenone is activated intramolecularly, and after deprotonation is confined as the silyl enol ether.

**Scheme 11:**
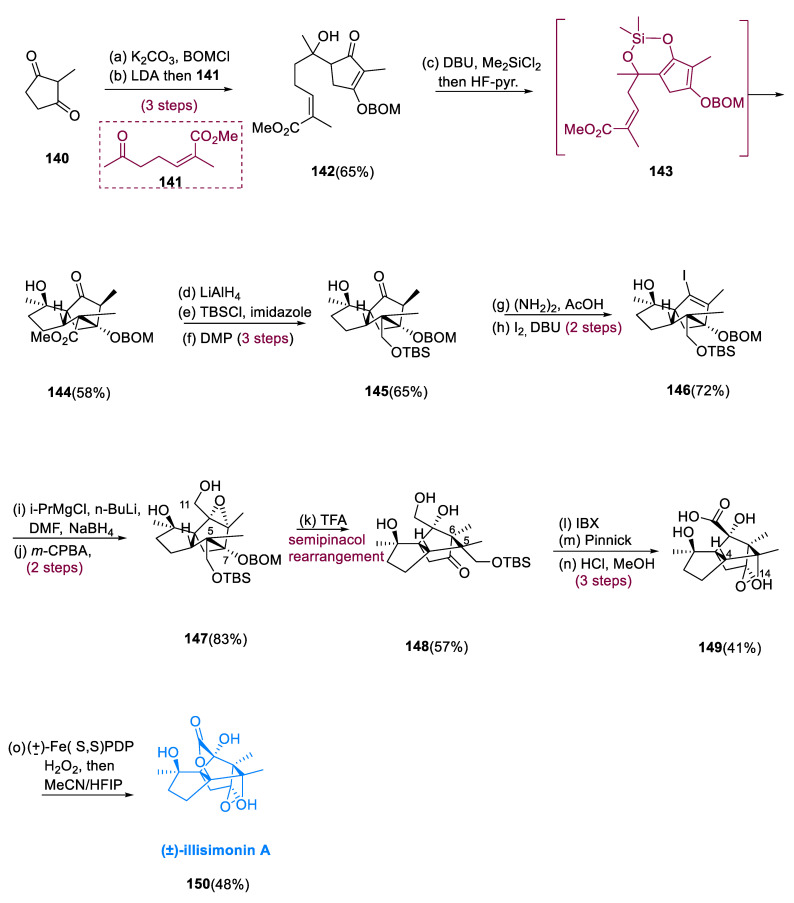
Total synthesis of (±)-illisimonin A.

The methyl ester had to be converted to the main alcohol. Worldwide lithium aluminum hydride reduction (LAH), undergoing TBS (tertiary butyldimethylsilyl ether) protection of the newly yielded primary alcohol, and C10 ketone reoxidation provided the most straightforward transition, yielding molecule (**145**) in overall good yield (65%). Barton’s technique was used to make vinyl iodide (**146**). The crude allylic alcohol was produced by Bouvealt aldehyde and simultaneous reductions, which was then oxidized with *m*-perchloro benzoic acid (*m*-CPBA) to create epoxide (**147**) in a satisfactory yield, which was oxidized to a lactone (**150**) by White’s FePDP with H_2_O_2_ as the stoichiometric scavenger after being handled with TFA and reduced into acid (**149**) (Scheme 11) [78].

### 4.4. Dysidavarone A

Lin et al. reported in 2012 that dysidavarone A (**162**), a new sesquiterpenoid quinone, was isolated and structurally elucidated from *Dysidea avara*, a marine sponge gathered along Yongxing Island located in the sea of South China. With IC_50_ values of 9.98 and 21.6 mm, these natural marine compounds have exhibited inhibitory efficacy against PTP1B (proteintyrosine phosphatase 1B) [79]. PTP1B is a main adverse regulator in leptin and insulin signaling pathways, as well as a positive regulator in malignancy and cancer development [80].

Under the Birch condition, the crucial reductive alkylation of enone (**151**) with molecule (**152**) went quickly and effectively, yielding the predicted coupling compound (**153**) in an 81% yield as the only diastereomer. Deprotection in the molecule (**153**) of the TBS group resulted in the production of hemiacetal (**154**) in a 98% yield. To directly synthesize the quinone system, hemiacetal (**154**) was allowed to be treated with an O_2_ balloon (molecular oxygen) in acetonitrile at room temperature for 15 h in *N, N′*-bis (salicylidene)ethylene diaminocobalt(II) (salcomine), yielding the required quinone (**155**) (86%). The formation of methoxyquinone (**156**) in high yield was achieved by reacting quinone (**155**) with five equivalents of LiN (SiMe_3_)_2_ in the presence of two equivalents of CuBr•Sme_2_ in a diluted THF solution from 40 °C to r.t. for 48 h (84%).

**Scheme 12:**
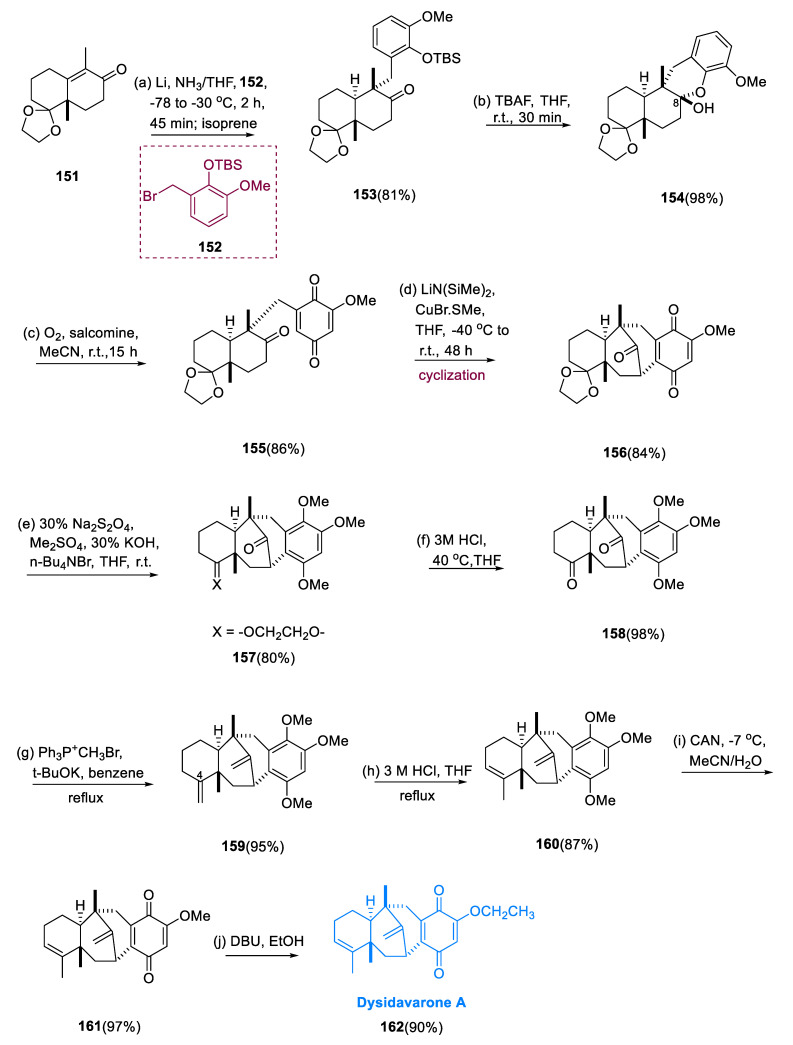
Total synthesis of dysidavarone A.

Reductive methylation was used to convert methoxyquinone (**156**) to trimethoxybenzene (**157**) (80% yield) involving 30% Na_2_S_2_O_4_, Me_2_SO_4_, 30% KOH, nBu_4_NBr, THF, r.t.). The resultant diketone (**158**) underwent Wittig methylenations to create bis-exo-olefin (**159**) in high yield (95%) after deprotection of the ethylene acetal ring in trimethoxybenzene (**157**) (98% yield). The equivalent endo-olefin (**160**) was produced in an 87% yield undergoing site-selective isomerization of the C_4_ exo-olefinic bond in olefin (**159**). Then, the quinone system was successfully regenerated by treating endo-olefin (**160**) with CAN (ceric ammonium nitrate) in H_2_O/MeCN at low temperature, resulting in a 97% yield of the required methoxyquinone (**161**). Finally, when methoxyquinone (**161**) was exposed to ethanol in DBU (diazabicyclo-undecene) at r.t., the desired dysidavarone A (**162**) was formed in a 90% yield (Scheme 12) [81].

### 4.5. Rumphellaone A

Rumphellaone A (**173**) was obtained from the *Rumphella antipathies* gorgonian coral, which has also synthesized many other caryophyllane and clovane sesquiterpenes [82]. Rumphellaone A (**173**) contains 4,5-seco-caryophyllane with an extraordinary γ-lactone species that is cytotoxic to *T* cells against acute lymphoblastic meningioma cells having IC_50_ values of 12.6 g/mL [83].

Total synthesis of product (**173**), rumphellaone A, started from a molecule having (*R*)-(**164**), which was obtained via *O*-iodo benzyl bromide alkylation, coupling with HC_2_Si(Me_3_)_3_ via Sonogashira, and TMS deprotection from commercially accessible (*R*)-**163** (methyl heptenol). In a 75% average yield, the major diastereomer of cyclobutane (**166**) was achieved with (97.3:2.7 er). The stereoselective reduction of the double bond of the cyclobutane (**166**) was subsequently investigated under various conditions, including Pd-catalyzed hydrogenation and cleavage of (C-H and C-C) bond of the benzylic ether, which simultaneously yielded alcohol (**167**). Following acetal and ketone protection, aryl ring cleavage oxidatively generated cyclobutane derivatives (**168**) (carboxylic acid), which were almost quantitatively yielded after methylation and ketone protection. The requisite trans-geometry at the ring containing cyclobutane moiety was accomplished via epimerization of ester on the α-position, providing pure trans-(**170**), yielding 86% in epimerization hydrolysis.

**Scheme 13:**
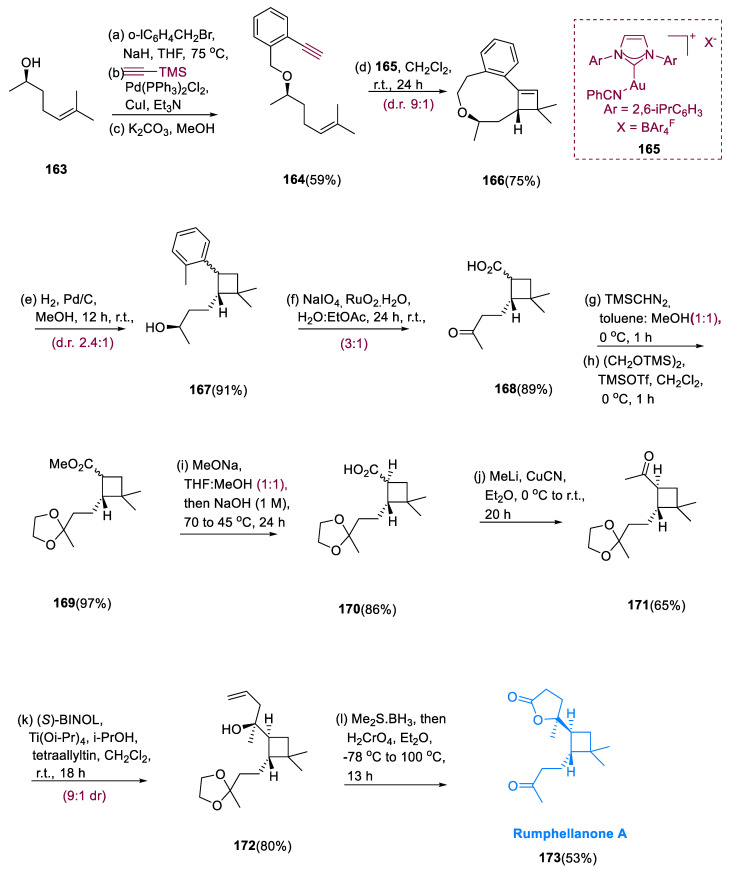
Total synthesis of rumphellanone A.

Methyl lithium and CuCN underwent treatment with molecule (**170**), resulting in methyl ketone (**171**). The required tertiary alcohol (**172**) was synthesized in an 80% yield with diastereoselectivity (9:1) using (*S*)-BINOL. The allylic alcohol (**172**) underwent reverse Wacker oxidation to yield the final γ-lactone. The final product was a lactol that was oxidized with PCC to yield the target lactone product (**173**), rumphellaone A (Scheme 13) [84].

### 4.6. Thapsigargin

Thapsigargin (**186**) was discovered in 1978, although it was used as a popular folk medicine in prehistoric eras. Thapsigargin (**186**) is a classic target for total synthesis as it is common oxidizing species of the ancient guaianolide family [85]. The prodrug derivative of thapsigargin (**186**) in latestage clinical trials is currently being used for several tumors. Thapsigargin (**186**) is a robust antagonist against the protein SERCA-pump with promise to be used in a scope of healing areas [86,87,88].

To make decalin (**176**), we started via Robinson annulation between (+)-(**175**) (dihydro carvone) and (**174**) (ethyl vinyl ketone). After the annulation event, exposing the mixture to an O_2_ atmosphere leads to installation of the alcohol at the C-6 (_Ƴ_-position) diastereoselectively. In one step, in a sequence involving bromination and elimination, dienone (**177**) from decalin (**176**) was efficiently obtained in an 85% yield. Diol (**178**) was obtained in a 60% yield by hydrolysis of (**177**) with Burgess solution and dihydroxylation via chemoselective/diastereoselective 5:1 ratio with AD-mix-α of terminal olefin. This step was responsible for the following oxidation with the diol (**178**) at C-8.

Selective protection of primary alcohol, simultaneously involving CH allylic oxidation via diastereoselectivity with SeO_2_, provided allylic alcohol (**179**). Mitsunobu inversion with butyric acid led to the simple fitting of the butyrate **(180)** with the preferred stereochemical geometry at C-8 after the allylic alcohol (**179**) was synthesized. Interestingly, at trans-synthesis, at C-11 the major targeted stereoisomer reacted favorably, leading to a diastereomerically 10:1 dr abundant product (**180**). Enone (**181**) was obtained at 50% by irradiating compound (**180**), by 0.01 M in CH_3_COOH via a mercury lamp. The required α-octanoylated enone (**182**) was obtained by treating enone (**181**) with KMno_4_ in benzene refluxing in octanoic and octanoic anhydride.

**Scheme 14:**
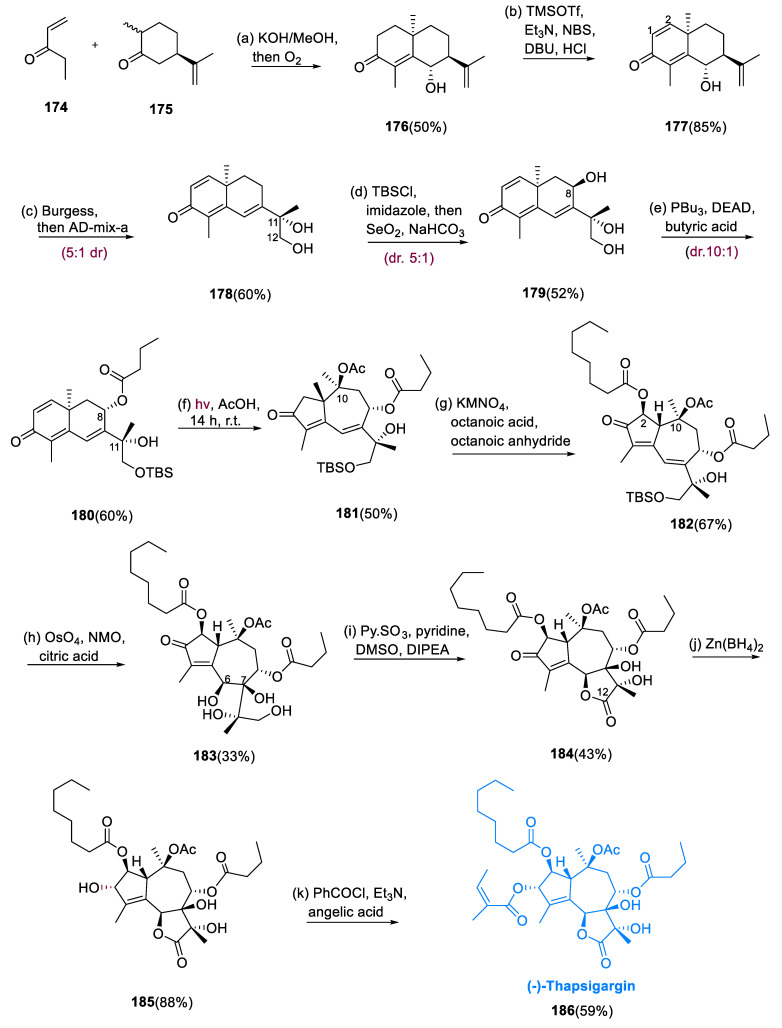
Total synthesis of (–)-thapsigargin.

While efficient, dihydroxylation of α-octanoylated enone (**182**) with stoichiometric OsO_4_ was difficult due to the prolonged approach to adding C-6 and C-7 oxygen atoms. After many experiments, it was observed that using the Upjohn method employing citric acid made the reaction catalytic at 50 °C. Molecule (**183**) was synthesized in a 33% yield under these reaction conditions, coupled with TBS tetraol (23%). Lactonization under Parikh–Doering conditions yielded lactone (**184**) via the reasonably stable lactol, possibly via the intermediacy of a lactol. The penultimate product (**185**), as the final natural product, is known to be potentially noxious at a very low quantity; lactone (**184**) was chosen as the synthesis’s end point. Using zinc borohydride conversion and esterification through anhydride of benzoyl chloride/angelic acid, an analytically pure compound (**186**) was prepared from lactone (**184**) (Scheme 14) [89].

### 4.7. α-Ekasantalic Acid

Illicium lanceolatum produces tricyclane sesquiterpenoids [90]. Sesquiterpenoids contain the tricyclane ring [tricycle [2.2.1.02,6] heptane] in their configuration. Antitumor, antifungal, and bactericidal activities were found in them. They are attached to tubulin and are responsible for initiating the G2/M cell cycle and leukemia cells (HL-60); they suppress migration of MCF-7 breast tumor cells and MDA-MB-231 by attacking the *β*-catenin pathway, an antagonist of D2 and 5-HTreceptors [91,92,93,94]. They are also proven to have antibacterial properties against the oral pathogen *Porphyromonas gingivali* as well as moderate activity against *Helicobacter pylori* [95]. Their derivatives included the proapoptotic cytotoxin with selectivity for cancer cells over healthy cells at sub-μM dosage, as well as longicyclene, a metabolite that reduces the SOS effect caused by biochemical mycotoxins [96,97].

The sodium carboxylate salt (**188**) was selectively acquired by treating cycloadduct (**187**) with sodium isopropoxide. Regioselective ring opening of acid anhydride occurs due to steric factors. Because of the methyl substituent, the bulky CH(Me)_2_O^−^ (isopropoxide anion) resists targeting the carboxyl group adjoined to the quaternary carbon. Furthermore, the epimerized ester group is sterically less hindered and hence a thermodynamically favorable exo-site under various basic reaction conditions. Bromide (**190**) is produced by treating sodium carboxylate salt (**188**) with Br_2_/H_2_O. The reaction proceeds via Br^+^ synthesis on the alkene’s sterically less hindered face, by a nucleophilic carboxylate interaction on the backside.

In tetrahydrofuran, treatment of bromide (**190**) with K^+^(Me)_3_CO^−^ led to enolization of the noncyclic ester, intramolecular cyclization, and removal of potassium bromide, giving intermediate (**191**). Under acidic conditions, the noncyclic ester was selectively hydrolyzed in intermediate (**191**) to only become a molecule (**192**). In the pivotal step, alcohol (**193**) was obtained by reducing the carboxylic acid function in the molecule (**192**) with borane. To make bisalcohol (**195**), alcohol (**193**) was converted to iodide (**194**), which was then treated with LiAlH_4_. Because aldehydes were more reactive than ketones in Wittig reactions, we reduced the bisalcohol (**195**) to compound (**197**) by PCC (Swern oxidation) to chemically differentiate the two hydroxyl groups.

**Scheme 15:**
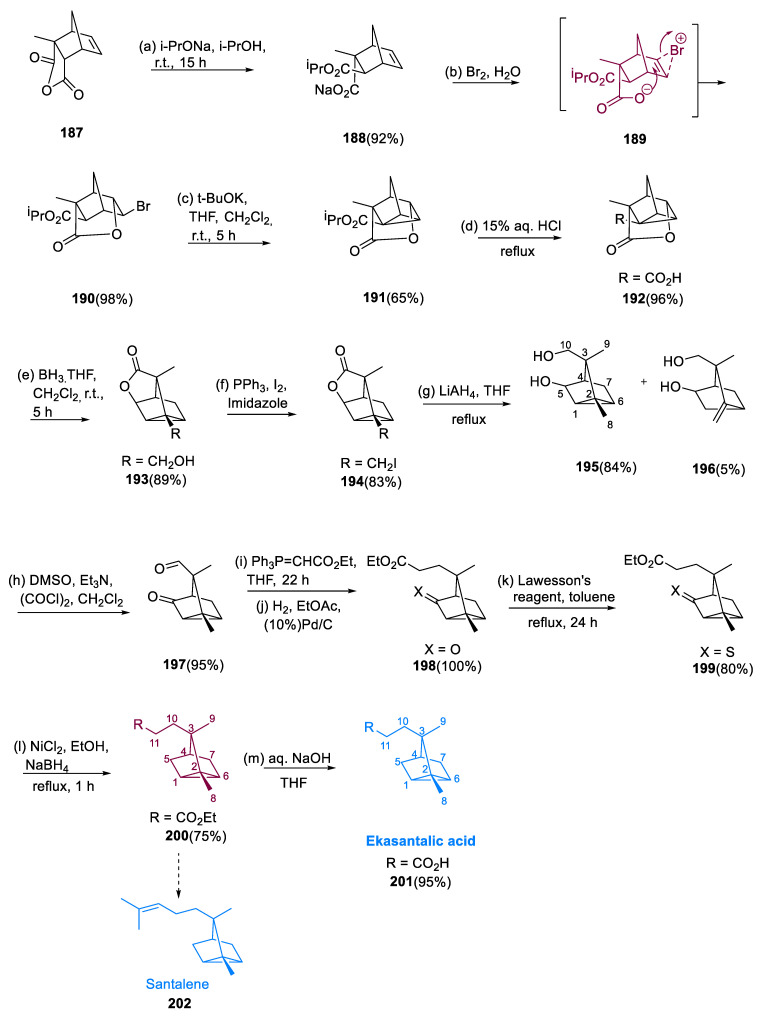
Total synthesis of α-ekasantalic acid.

Keto ekasantalic acid ethyl ester (**198**) was obtained by treating the compound (**197**) with the relevant Wittig substrate and hydrogenating the obtained alkene. The five-keto group was reduced by converting it to thione (**199**) via Lawesson’s reagent, then reducing it with NiCl_2_ and NaBH_4_ to obtain the compound (**200**). The hydrolysis of (**200**) resulted in the formation of α-ekasantalic acid (**201**). This is the first time that (±)-α-ekasantalic acid (**201**) has been synthesized entirely. In the whole synthesis of α-santalene (**202**), as a target molecule, the compound (**200**) is a recognized precursor to synthesizing significant active molecules (Scheme 15) [92].

## 5. Diterpenoids

### 5.1. Crotogoudin

Crotogoudin (**214**), a cytotoxic diterpene isolated from local *Croton* plants, was isolated by a Madagascan–French group in 2010 [98]. This genus species has long been used in ethnomedicine around the world and is a rich source of secondary metabolites having a vast range of biological activity, as well as a source of interest for synthetic chemists [9]. Crotogoudin is a diterpene from the rare 3,4-secoatisane family that has displayed cytotoxicity with an IC_50_ of 40 μM against the human lymphocytic leukemia (K562) cell line and 139 μM against rat hepatocytes, and is also used for the cure of fungal infections, fever, sexually transmitted disorders, malaria, diabetes, inflammation, cancer, and digestive problems [99,100].

A progressive methylation/allylation technique was used to make the (S)-carvone derivative (**203**). After conjugate reduction with L-Selectride^®^ (C_12_H_28_BLi) and oxidative work-up, the corresponding ketone (**204**) is obtained, which would then be deprotonated and treated with Comins’ reagent to yield vinyl triflate (**205**). Heck reaction of vinyl triflate (**205**) with ethyl acrylate as the cross-coupling partner generates an ethyl enoate (**206**). Ester allyl group (**206**) was transformed into the propyl hydroxy group. This was obtained by hydroboration of the allyl group chemoselectively in the vicinity of the isopropenyl moiety using Wilkinson’s catalyst (C_54_H_45_ClP_3_Rh) (**207**), a well-known catalyst for the hydrogenation of unsaturated hydrocarbons/catecholborane, by oxidation of the alkylborane. Then, saponification of the intermediate hydroxy ester delivered acid **(208)** in an 83% yield. After warming molecule (**208**) in (MeCH_2_CO)_2_ to 180 °C for 5 d, benzannulated bis-propionate bicycle (**209**) was produced in an 82% yield.

Tetramethylguanidine (**210**), a strong nucleophilic base for alkylations, was utilized to selectively cleave the phenyl propionate in a molecule (**209**). This opened the route for position-specific oxidative dearomatization, which formed dienone (**211**). The intention was to perform a [4+2] cycloaddition of cyclohexadiene (**211**) with ethylene diastereoselectively. Under pressure (70 bar) and 140 °C for 5 d, undergoing cycloaddition generated tricycle (**212**) (6:1) in a 90% yield. The dimethyl ketal and propionyl groups were both removed by Wittig olefination, which was carried out by acid hydrolysis of the ketone group.

**Scheme 16:**
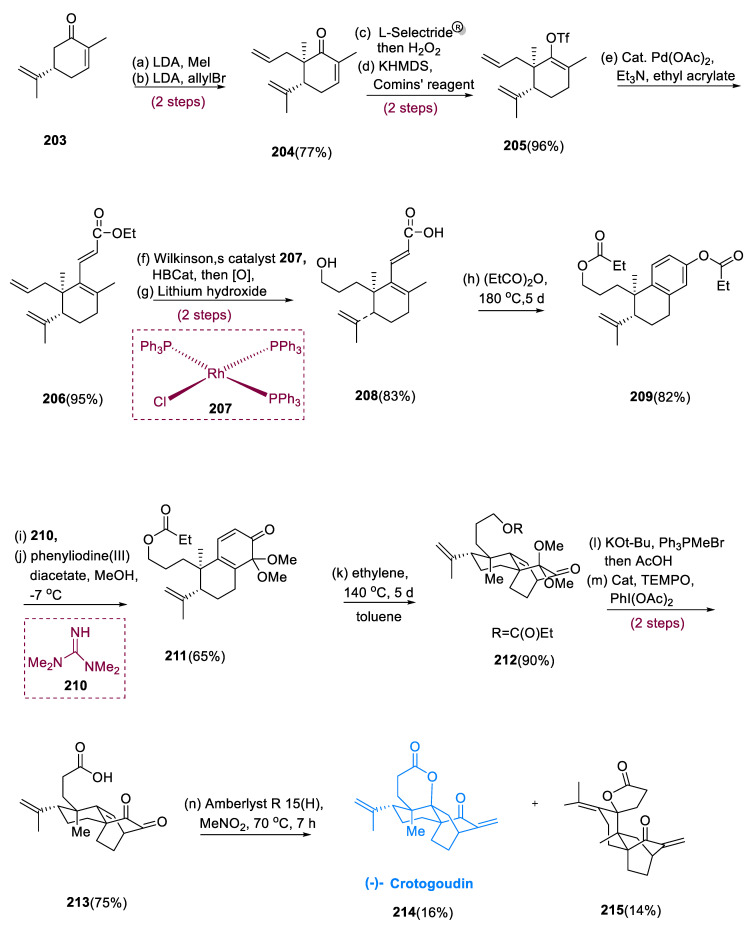
Total synthesis of (–)-crotogoudin.

Over two processes, the main hydroxyl moiety was oxidized to the carboxyl function, providing (**213**) seco crotogoudin in a 75% yield. The lactonization of tricycle (**212**) to obtain (–)-crotogoudin was afflicted with difficulties. Consequently, conditions were found that produced crotogoudin (**214**) in a yield of 16% (2.9%) and rearranged lactone (**215**) in a yield of 14% (Scheme 16) [101].

### 5.2. Waihoensene

The diterpene waihoensene (**230**) was isolated from *Podocarpus totara*, a native plant by the Weavers group in 1997 in New Zealand, and exhibited anticancer activity mainly towards (A-549) lung cells [102,103]. Waihoensene contains a tetracyclic joined ring framework imbedded in it, with six stereocenters, four of which are quaternary stereocenters, leading to the discovery of various types of drugs [104,105].

Starting with the commercial substrate (**216**), the synthetic chiral process was initiated. The quaternary site was inserted into molecule (**216**) through alkylation employing propargylic triflate (**217**), which generated vinylogous ester (**218**) in an 80% yield. The addition of lithiated (**219**) to molecule (**218**), vinylogous ester, was succeeded by an in situ elimination process, yielding enone (**220**) in an 89% yield. Through a radical cyclization reaction, the required hydrindane scaffold (**221**) was formed at C-7 (diastereomeric mixture) in 95%. By equilibration, the required stereochemistry of the methyl group at C-7 was achieved via destannylation of hydrindane (**221**) and later treated with NaOH in MeOH, yielding 97% of the desired cis-geometry (**222**) in an 88% yield.

The reduction in 1,3–allyl strain is responsible for the high preference for the desired structure of the C-7-methyl-group in the compound (**222**). The ketone compound (**222**) was defunctionalized by transferring it to hydrazone (**223**) and then reducing it with catechol borane to yield compound (**224**). Deprotection under Birch conditions, Doering–Parikh oxidation to the appropriate aldehyde, and Bestmann–Ohira alkynylation were used to convert it into alkyne (**226**). The precursor for the reaction of Pauson–Khand is alkyne (**226**), which was reacted with Co_2_(CO)_8_ (dicobalt octacarbonyl) to generate the cobalt–alkyne framework. Under stressful situations, this key intermediate was cyclized and carbon monoxide was inserted to give 46% of the target product (**227**) and therefore deliver the fully formed nucleus of waihoensene (**230**).

**Scheme 17:**
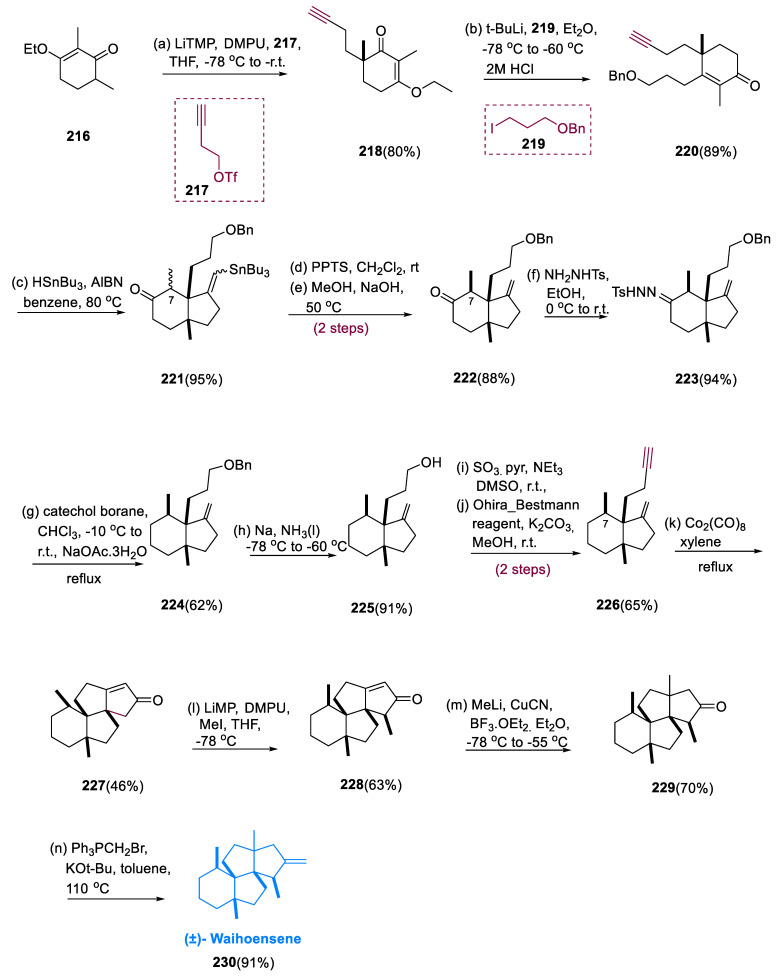
Total synthesis of (±)-waihoensene.

A three-step synthesis was used to initiate the α-alkylation of compound (**227**) (Pauson–Khand) to yield compound (**228**) as a major diastereomer. Waihoensene (**230**) was synthesized by adding a Me group to enone (**228**), followed by α-alkylation/olefination. For the competing emergence of thermodynamic and kinetic enolates, the choice of base in this α-alkylation of enone (**228**) is important. Strong bases such as LiTMP, LiICA, and LDA were treated with DMPU, generating the requisite methylated compound (**228**) as the sole intermediate. Thermodynamically stable enolates (deprotonation at the γ-position of the enone substrate) were formed when weaker bases, such as LiHMDS, are used. Methyl cuprate following 1, 4-addition to compound (**228**) yielded molecule (**229**) in 70%, resulting in a single isomer. Then, it was accomplished with Wittig olefination, which yielded 91% of the natural product waihoensene (**230**) (Scheme 17) [106].

### 5.3. Leonuketal

Leonuketal (**246**) has 8, 9-Seco labdane tetracyclic terpenoid with a large number of geometric and stereochemical structures, due to a C-C breakage process during synthesis. Peng and colleagues extracted leonuketal from *Leonurus japonicas* (Chinese liverwort) and found that it has a strong vasodilating activity, with EC_50_ values of 2.32 μM, against which KCl activation leads to the narrowing of the rat aorta [107].

Treatment of epoxide (**231**) with in situ-generated Cp_2_TiCl_2_ efficiently afforded bicyclic ketone (**232**) after acidic hydrolysis. The stiffness produced by the auxiliary ring may have increased the diastereoselectivity seen for the synthesis of compound (**233**), encouraging the equatorial method of L-selectride and inhibiting ketone ring flipping. Over two processes, compound (**233**) was reduced to tosyl hydrazone, which was then treated with MeLi, yielding alkyne (**235**) in an 89% yield. This process most likely began with the formation of vinyl lithium (**234**), which was then β-eliminated. The iodide obtained from intermediate (**23**) was made in three processes, first with the hydroxymethylation of the alkyne (**236**), then mesylation, and finally iodination. Alkyne (**239**) was obtained in an 85% yield by treating β-ketoester (**238**) with NaBH_4_/NaH and the iodide generated from intermediate (**237)**. Alkyne (**239**) was a combination of C-10 epimers, and the C-11 ketone would contain four diastereomers if reduced. As an alternate spiroketalization substrate, deprotection of TBS and lactonization of the alkyne (**239**) yielded cyclic β-keto (enol) lactone (**240**). Treatment of lactone (**240**) with PPTA and AuCl•DMS consistently produced spiroketal (**241**) in a 65% yield.

**Scheme 18:**
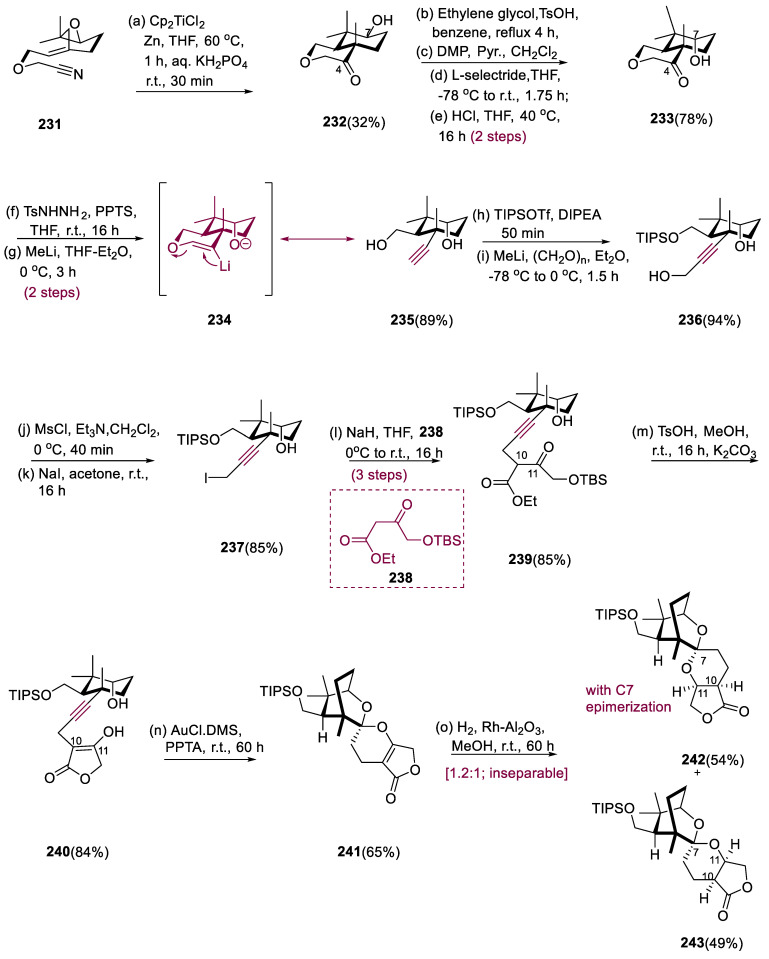

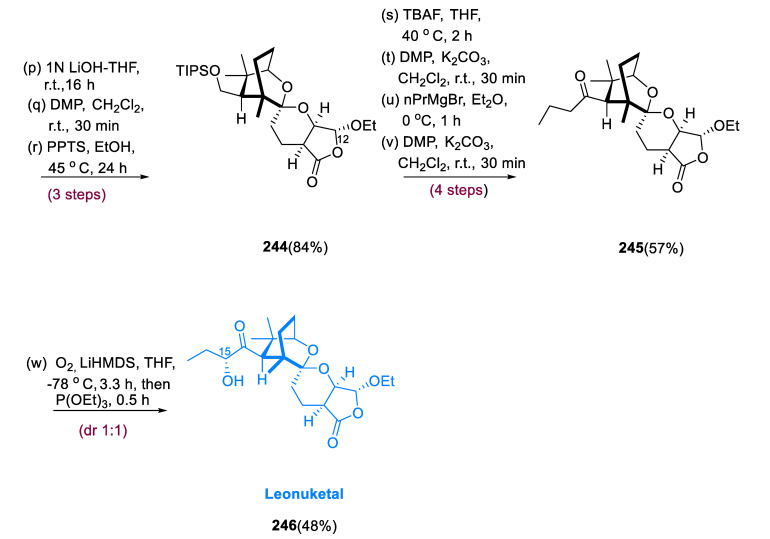
Total synthesis of leonuketal.

The mixture of compounds (**242**) and (**243**) observed is likely the result of nonselective hydrogenation of spiroketal (**241**) from either side of the alkene, resulting in a mixture of the intended hydrogenated compounds (**242**) and 7-epi-(**243**). Compound (**244**) was deprotected at a slightly higher temperature, and the liberated alcohol was quickly oxidized through DMP to inhibit transketalization. Over four steps, the aldehyde was reacted with propyl-magnesium bromide before being oxidized directly to give (**245**) deoxyleonuketal in a 57% yield. The desired oxidation of deoxylenuketal (**245**) was achieved by deprotonation with LiHMDS, which was further followed by treatment with (*1S*) (+) (10-camphorsulfonyl) oxaziridine, but the production of 15-epi-leonuketal (**246**) was favored. After reductive workup, bubbling O_2_ and deprotonated (**245**) provided leonuketal (**246**) and 15-epi-leonuketal (epi-**246**), in a 48% yield (1:1) dr (Scheme 18) [108].

### 5.4. Epoxyeujindole A

Nakadate et al. reported the extraction of epoxyeujindole A (**265**), a structurally complicated anominine from *Eupenicillium javanicum*, in 2011 [109]. Through a biomimetic synthesis, epoxyeujinode A (**265**) has a heptacyclic structure [110]. It has anti-inflammatory properties [111].

**Scheme 19:**
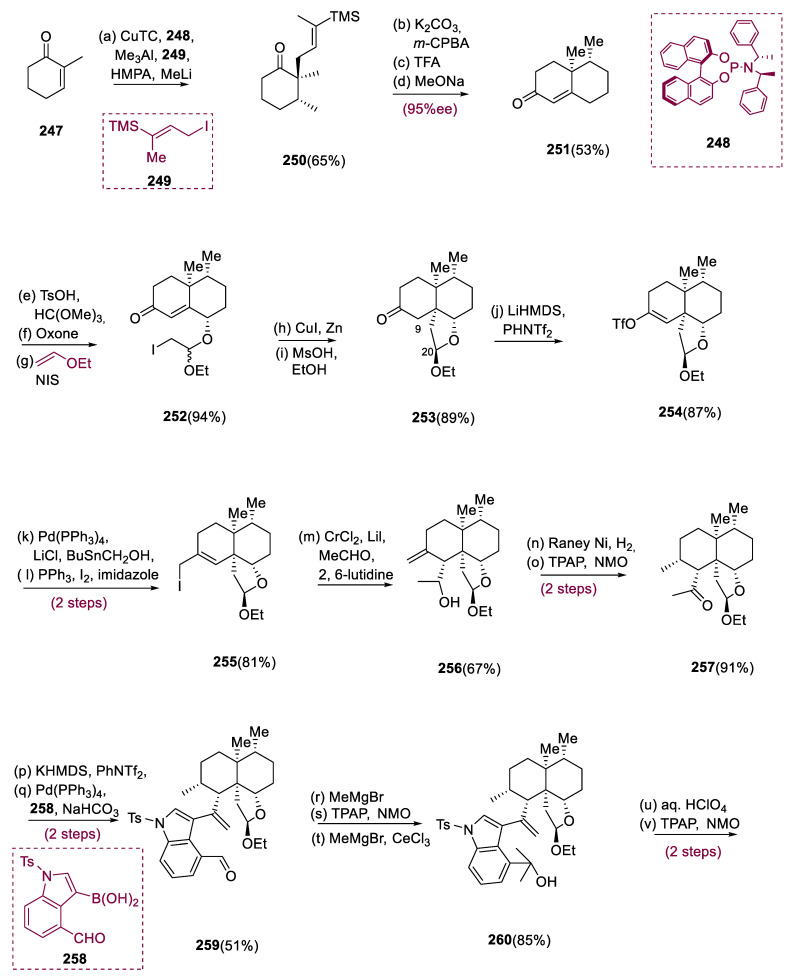

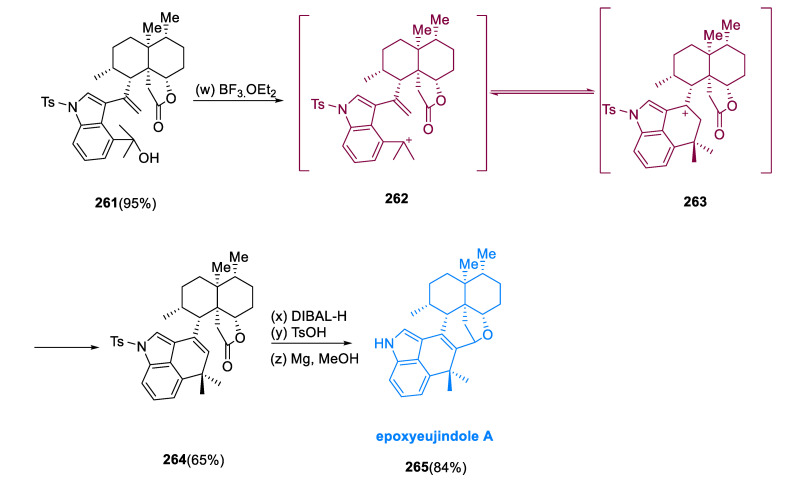
Total synthesis of epoxyeujindole A.

To set the first stereocenter, the conjugate addition of CuTC, Me_3_Al (**247**) with phosphoramidite ligand (R, S, S)-(**248**) occurred. The resulting enolate was initiated in situ with methyl lithium and HMPA before being quenched with allyl iodide (**249**), giving ketone (**250**), with excellent enantioselectivities. Allyl iodide (**249**) was a suitable substitute for methyl vinyl ketone. The epoxidation of the alkenyl silane involving *m*-CPBA was followed by TFA resulting in the equivalent diketone, which proceeded with aldol condensation employing MeONa to give enone (**251**) in a 53% total yield. This product **(251)** was treated with a well-known three-step mechanism to obtain iodide (**252**). In the case of reaction scalability/purification, the Luche protocol was more effective than conventional radical protocols. This mixture of diastereomeric acetal (1.4:1) at C-20 was smoothly transformed to the thermodynamically more stable compound (**253**) by exposing it to MsOH in ethanol. Treatment with LiHMDS affected regioselective deprotonation **(254).** The Li enolate was masked by PhNTf_2_ to give triflate (**254**) in an 87% yield, which was subsequently iodinated via Ph_3_P, I_2_, and imidazole, followed by Stille–Migita coupling [Bu_3_SnCH_2_OH, Pd(PPh_3_)_4_, LiCl] to yield allyl iodide (**255**). The insertion of the two-carbon unit at C9 was accomplished using a Nozaki–Hiyama–Kishi coupling (LiI, CrCl_2_) with acetaldehyde, which resulted in alcohol (**256**) in a 67% yield as a single diastereomer. Under the coupling conditions, alcohol (**256**) tends to cyclize to make an acetal bridged. As a result, 2,6-lutidine is significant as a buffering agent. For the cross-coupling, NaHCO_3_ was employed as a weak base. A three-step strategy was used to convert aldehyde (**259**) to tertiary alcohol (**260**) with high overall (85%) efficiency. Due to the deprotonation of the methyl ketone substrate, the second methyl addition required the use of a Ceric reagent. The steps of acid hydrolysis via TPAP oxidation yielded compound (**261**) in a 95% yield; the benzylic tertiary hydroxyl was tolerated under these conditions. When compound (**261**) was exposed to BF_3_ •OEt_2_, a ring closing occurred, yielding hexacycle (**264**) in a 65% yield. The vinylogous reaction of Friedel–Crafts can happen mainly to the intermediacy of intermediates (**262**) and (**263**). The heptacyclic framework of the natural product was produced through Prins cyclization by reinstalling the hemiacetal function group via DIBAL-H reduction by treating TsOH. In an 84% yield, reductive desulfonation happens with Mg in methanol-produced epoxyeujindole A (**265**) (Scheme 19) [112].

### 5.5. Sculponeatin N

A bioactive polycyclic containing diterpene sculponeatin N (**281**), isolated from *Isodon sculponeatus*, is produced from the parent ent-kaurene and contains 6, 7-Seco-terpenes. Sculponeatin N (**281**) has an action against the K-562 and HepG-2 cell lines of IC_50_ values of 0.21 and 0.29 mm, respectively [113].

Compound (**267**) is formed by the conjugate addition of methyl cuprate/formaldehyde aldol to 3-methyl cyclohex-2-enone (**266**). Peterson olefination protects the main alcohol, yielding enolate (**267**) in 49% overall (three steps). Dienone (**269**) was synthesized from ester enolate (**267**) via its Weinreb amide, a smooth reaction with organolithium (**268**). Following reprotection, cyclopentenone (**271**) may be synthesized as a single diastereoisomer in an 80% yield. The diastereoselective ring-closing metathesis reaction of tris-(allyl) intermediate (**271**) results in diene (**272**). The addition of halide (**270**) to cyclopentenone (**271**) yielded intermediate (**272**), which achieved 1, 4-addition in excess BF_3_•Et_2_O (78% yield). The elimination of TBS ethers, followed by ketone allylation and double Grieco elimination, resulted in tris-(allyl) intermediate (**272**). The exposure of intermediate (**272**) to the Grubbs (II) catalyst produced the required cis-hydrindane (**274**) in a 91% yield. During the reaction, compound (**272**) was initially divided, one of which was related to the product (**274**) and another into spirocycle (**273**) as the reaction proceeded. We were able to determine that this other species, spirocycle (**273**), was added with Grubbs (II) catalyst resulting in the synthesis of cis-hydrindane (**274**). Following Wacker oxidation of the terminal olefin to generate compound (**275**) (methyl ketone), diene (**275**) was converted to enol triflate (**276**), which undergoes selective triflation when exposed to KHMDS potassium-hexamethyl-disilazane and the Comins reagent.

**Scheme 20:**
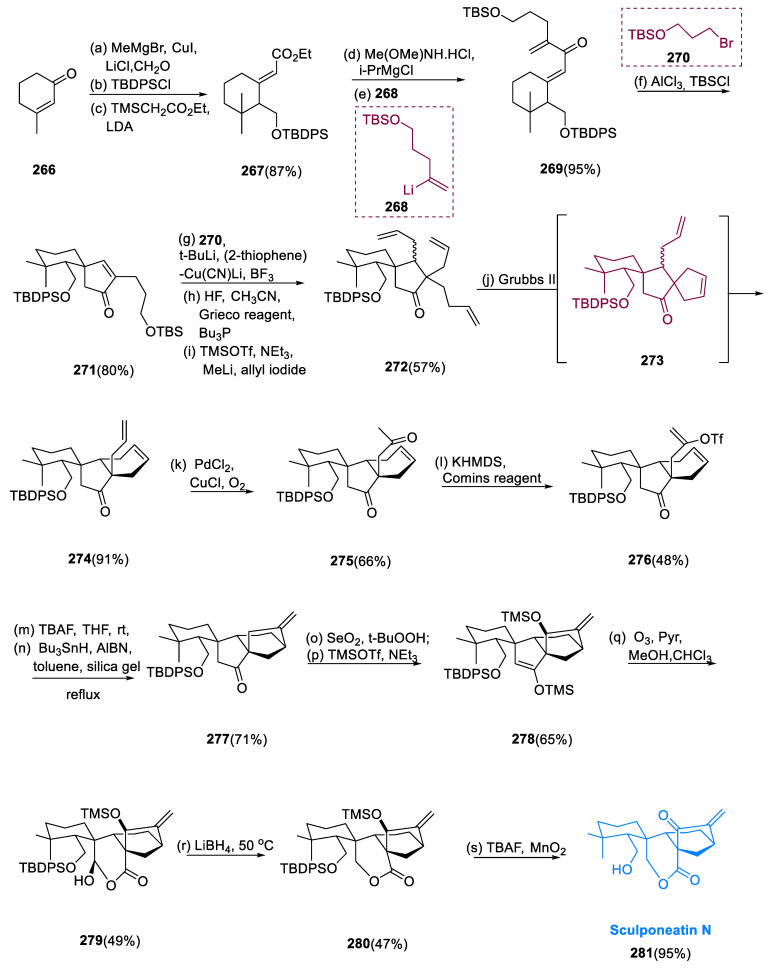
Total synthesis of sculponeatin N.

The requisite bicyclo[3.2.1]-octane functional moiety (**277**) was made from enol triflate (**276**). Competitive breakdown of the C16-C17 of exomethylene function in enol triflate (**276**) resulted in attenuation of this alkene’s reactivity, which was accomplished through allylic oxidation of compound (**277**) with SeO_2_/t-BuOOH, which transformed the resulting alcohol into bis-silylated species (**278**). After reductive workup with dimethylsulfide, bis-silylated species (**278**) were carefully exposed to O_3_ in the presence of C_5_H_5_N, yielding lactol (**279**). Using NaBH_4_ for the reductive workup instead of (Me)_2_S to directly convert secondary ozonide to a lactone (**280**) produced mainly lactol (**279**). At 50 °C, lactol (**279**) was exposed to a mixture of LiBH_4_ in diglyme and produced the required finding. The synthesis was subsequently easily completed by simultaneously removing both protecting silyl groups and selectively oxidizing the CH_2_=CHCH_2_OH with MnO_2_ giving sculponeatin N (**281**) in a 95% yield (Scheme 20) [114].

### 5.6. Canataxpropellane

Canataxpropellane (**305**), which belongs to the complex taxane group, was isolated from *Taxus Canadensis*. Diterpene containing highly oxygenated moieties belongs to one of the most complicated and sophisticated organic compounds interminably identified for the treatment of various cancers [115]. Canataxpropellane (**305**) is made up of a heptacyclic carbon structure [5,5,5,4,6,6,6]. In only two CH_2_ groups, it is highly functionalized and extremely oxidized, containing five hydroxyl groups and one ketone group [116].

Deprotonation of compound (**282**) followed by masking with tertiary butyltrimethylsilyl chloride (TBS-Cl) yielded isobenzofuran diene (**283**), to which dienophile (**284**) was added to generate Diels–Alder intermediate (**285**) in a 71% yield with high stereoselectivity. Irradiating (**285**)-(endo) (λ = 254 nm) in MeCN formed half of the bridge-like molecule (**286**). To facilitate the photoequilibrium, intermediate (**285**) was isolated from compound (**286**) and reintroduced to the photoreaction twice, yielding an inclusive photoadduct of 73%.

When compound (**286**) was treated with C_16_H_36_FN (tetra-butylammonium fluoride) in THF, it exclusively fragmented the C14–C20 link, yielding ketolactone (**287**). [Ca(BH_4_)_2_•2THF] calcium borohydride in CH_2_Cl_2_ reduced the keto group in ketolactone (**288**) with total selectivity to the equivalent alcohol at C-13. This alcohol performed in situ translactonization, yielding hydroxyl lactone (**289**), protected with (MOMCl) methoxymethylene chloride in a 73% yield over three steps. Bis-carbonyl (**290**) was produced by reducing compound (**289**) with (LiAlH_4_) by swern oxidation. When biscarbonyl moiety (**290**) was exposed to (KOtBu; 5:1) basic medium in THF/tBuOH, the following intramolecular aldol reaction with excellent diastereoselectivity restored the C14–C20 link and generated cage structure (**291**) in 53% isolated yields from compound (**290**). Endoperoxide (**292**) was produced as a single diastereomer by [4+2]-photooxygenation in the B-ring with singlet O_2_ of the diene system. The treatment of endoperoxide (**292**) with BHT (2, 6-tertiary butyl-4-methyl phenol) resulted in a clean reductive cleavage of the endoperoxide bond, yielding 71% of hydroxyenone (**293**). Selective allylic oxidation to the ketone at C5 with IBX (2-iodoxybenzoic acid), followed by the controlled reduction of ketone moiety with [NaBH(OAc)_3_] (sodium triacetoxy borohydride), assisted the nearby hydroxyl group at C–-20 initiated by intermediate (**294**), generating the proper configuration at C5 in compound (**295**). The 1,3–diols of enone (**295**), protected as the C_6_H_5_CH(OMe)_2_, proceeded via 1,4-reduction, yielding (**296**) in a 95% yield. The *B*-ring was formed by transforming the ketone at C-8 using Comin’s reagent (**297**) to the vinyl triflate and then Pd-catalyzed carboxy-methylation, yielding α, β-unsaturated ester (**298**) in a 77% yield. The appropriately saturated ester was obtained by dispersing metal reduction with Mg/MeOH, and subsequent α-alkylation with MeI, selectively installing the requisite geometry at the quaternary chiral center of C-8 to yield diol (**299**) in an 83% yield.

**Scheme 21:**
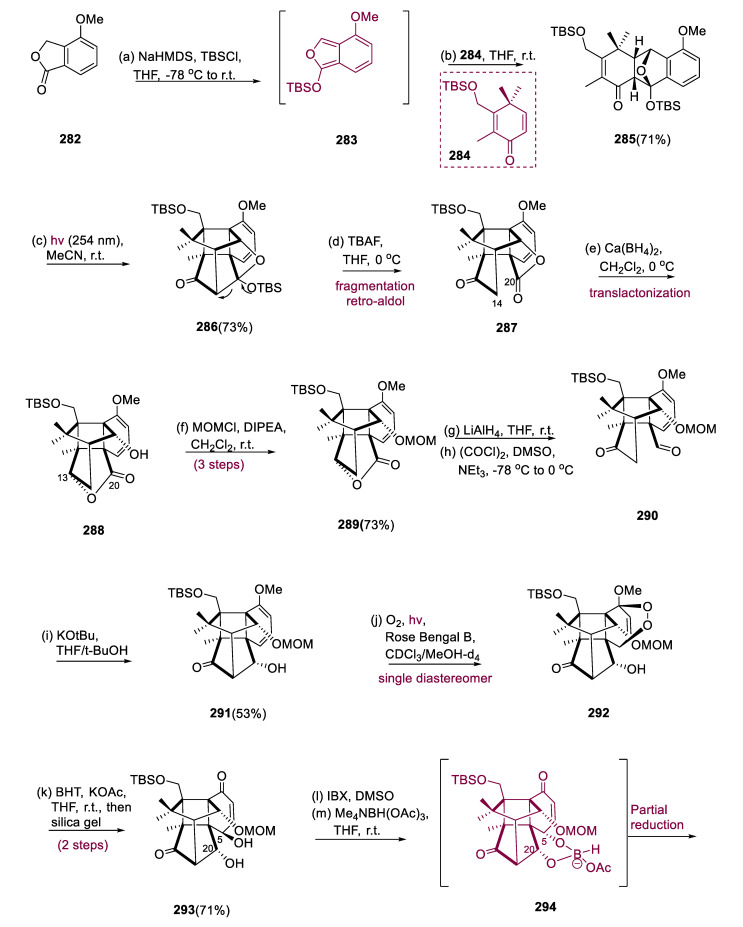

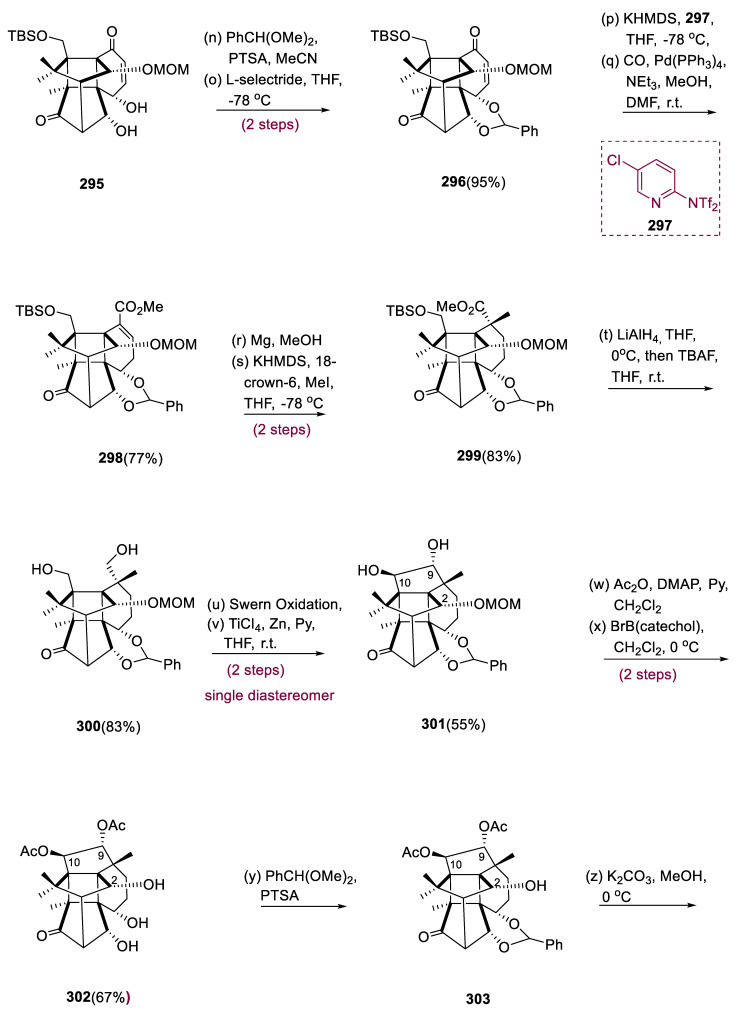

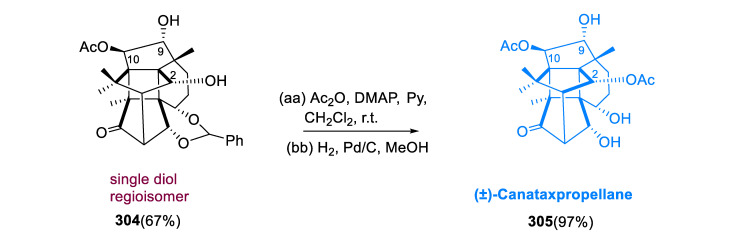
Total synthesis of (±)-canataxpropellane.

To produce diols, compound (**299**) was reduced with lithium-aluminum-hydride and the TBS group was eliminated with C_16_H_36_FN. The equivalent diols (301) were obtained by Swern oxidation. Pinacol (**301**) was formed as a single trans-stereoisomer as a result of the application of titanium tetrachloride (TiCl_4_)/Zn. We started with pinacol product (**301**) and acetylated it at both C10 and C9, then removed the methoxy-methyl with C_6_H_4_BBrO_2_ to obtain alcohol (**303**) and triol (**302**) from deprotection of the C_6_H_5_CH(OMe)_2_. The synthetic material was converged to alcohol (**303**) after reprotection of triol (**302**). The mono-deprotection of the acetate at C-9 successfully yielded diol (**304**) (67%). The hydroxyl group at C2 was selectively acetylated with regioselectivity (2.5:1) in a more reactive C-2 site. The benzylidene acetal was finally hydrogenated, yielding (±)-canataxpropellane (**305**) (Scheme 21) [117].

### 5.7. Crotophorbolone

Crotophorbolone (**348**) was isolated in 1934 as a phorbol degradation compound, and its structure was identified in 1969 [118,119]. It was isolated in 2010 from *Euphorbia Fischeriana Steud*, widely utilized in conventional Chinese treatment to treat edema, ascites, and cancer [120,121].

The dienoxysilane (**307**) was generated regioselectively by treating molecule (**306**) with TMSCl/MeMgBr in catalytic FeCl_3_. Following the vinylogous reaction of Mukaiyama-aldol of a molecule (**307**), the action of CH(OMe)_3_ and BF_3_.OEt_2,_ antiselectively connected the dimethyl-acetal moiety at the C-13 site to the bulky C-14 isopropenyl, yielding molecule (**308**) (5:1 dr). Formation of kinetic enolate by LDA from molecule (**308**) undertook the aldol reaction, accompanied by HCHO, which was instantly liberated from molecule (**309**).

The C14 substituent once again regulated the exclusive stereoselectivity in generating the C-8 center of a molecule (**310**). The conjugated C11 and C12 bonds underwent Birch reduction after the TIPS protection of molecule (**312**) to generate ketone (**313**). The sterically lithiated vinyl ether (**314**) was then equatorially added to the C-9 ketone (**313**), yielding the pentasubstituted cyclohexane (**315**).

When activated with CSA, the C-9 alcohol at the axial position of molecule (**315**) exchanged acetals with the C-13′ di-methyl acetal to form oxa-bicyclo[2.2.2]octane (**316**) (1:1 dr at C-13′). The *O*, *Se* acetal at C-9 was synthesized by converting the vinyl ether at C-9 of molecule (**316**). Thus, *m*-CPBA chemoselectively oxidized (**316**) vinyl ether to molecule (**317**) carboxylic acid via hydroxyl ketone. Following mesylation of molecule (**317**), the mesyloxy-carbonyl of the molecule (**318**) was transformed into the Aryl-Se function (**320**) in a one-pot reaction using photoirradiation and Barton ester in (PhSe)_2_. The three carbon extensions from (**322**) aldehyde produced from SO_3_•C_6_H_5_N-oxidation was followed through nucleophilic attack of vinyl lithium (**323**), resulting in the synthesis of the molecule (**324**).

The acetylation of two-hydroxy groups of the molecule (**324**) undergoes simultaneously to obtain the molecule (**325**). By *p*-allyl synthesis and site-selective reduction of acetate to the less congested primary site, a reagent combination of Pd°/KOAc rapidly converted the disubstituted olefin (**325**) into the trisubstituted olefin (**326**). The more exposed hydroxy group at C-20 of the ensuing diol (**327**) was terminated regioselectively with a hindered TIPS moiety after saponification, leading to the synthesis of molecule (**328**), and the residual hydroxy group at C-5 of (**328**), undergoing chlorination, yielding molecule (**329**). When molecule (**329**) was treated with substrate (**330**), CuTC, and K_2_CO_3_/DMF in [Pd(PPh_3_)_4_], the C-C synthesis carried on even at 0 °C, yielding (**331**) with no geometrical change.

When molecule (**331**) is treated with V-40 (**332**) and (TMS)_3_SiH, it yields ketone (**333**). Following β-elimination of the dimethyl-amino moiety with silica gel, ketone (**333**) was regioselectively converted into the TMS enol ether (**334**), and homologated to yield enone (**335**) with Eschenmoser’s reagent. RhCl_3_ efficiently facilitated the isomerization of exo-olefin (**335**) into the more stable molecule (**336**) endo-olefin. Acid hydrolysis was used to open the cyclic acetal of a molecule (**336**), and the partially deprotected C-20 hydroxy group was recapped with a TIPS to yield aldehyde (**337**). Carboxylic acid derivatives (**340**) were formed by oxidizing the (**338**) aldehyde to the (**339**) carboxylic acid and then silylating the 3°-hydroxy group at C-9 with TMSOTf.

**Scheme 22:**
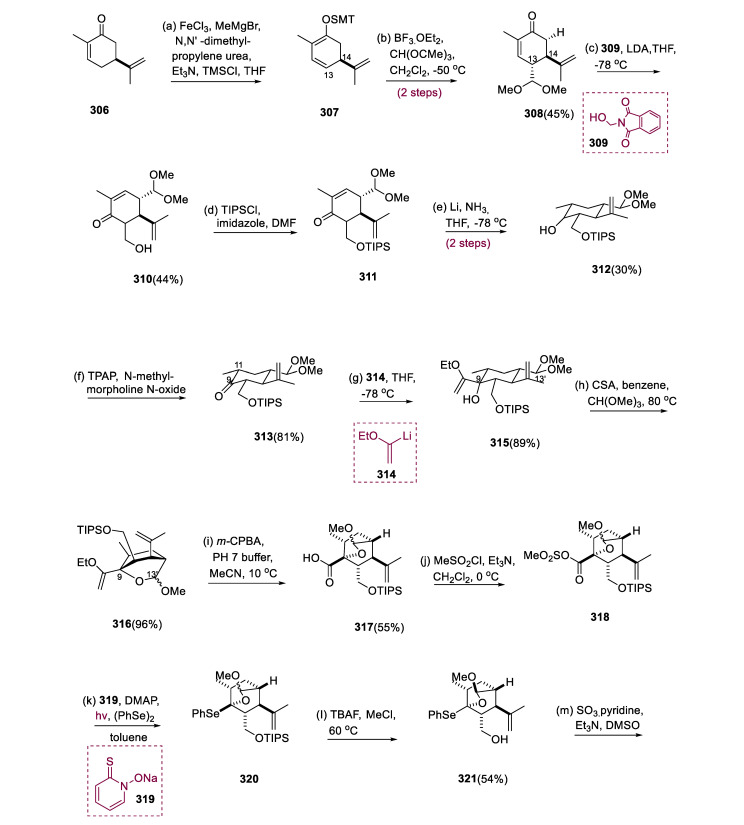

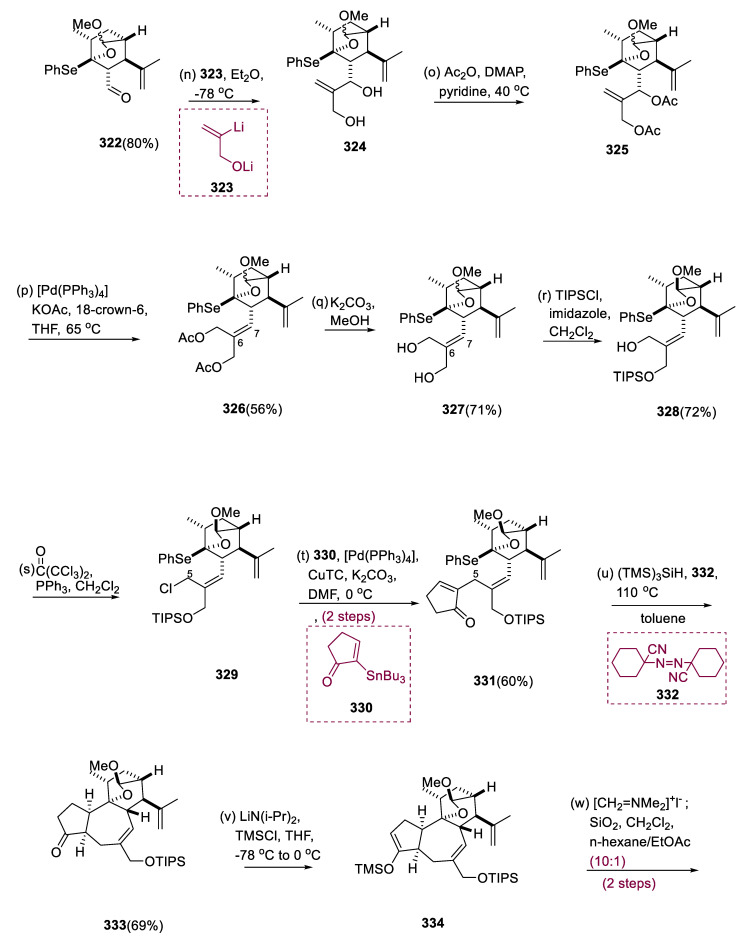

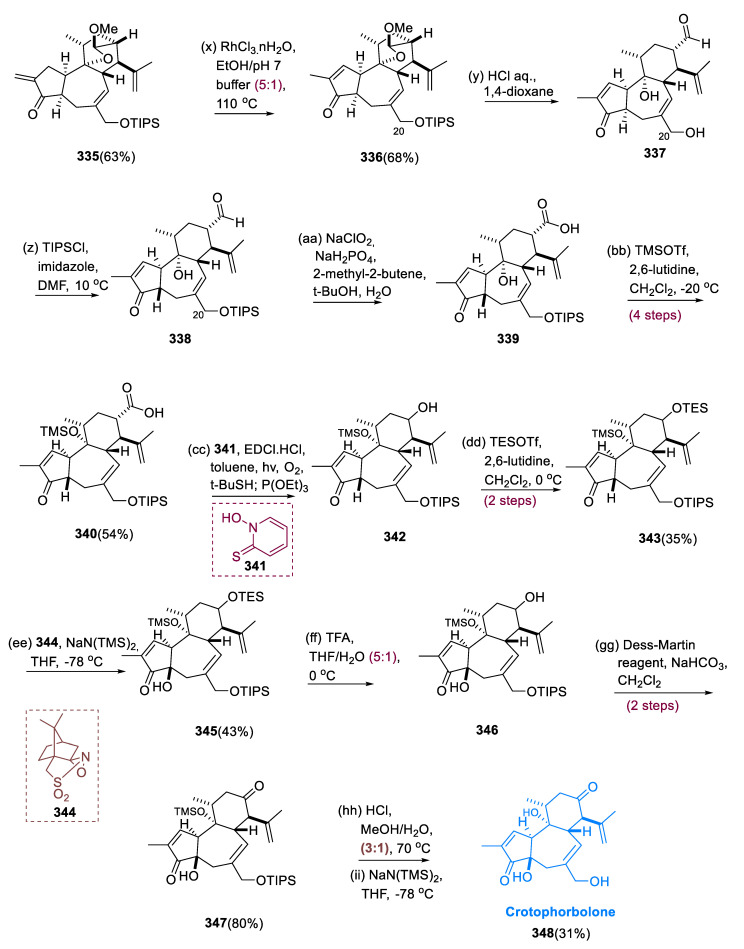
Total synthesis of crotophorbolone.

Finally, the COOH-based derivatives (**340**) were stereoselectively converted into the C-13 secondary alcohol (**342**) by the one-pot step: Barton ester with molecule (**341**) and EDCI•HCl; peroxide synthesis via photoirradiation in the presence of O_2_ and t-BuSH; and synthesis of reductive alcohol via P(OEt)_3_. The hydroxyl group at C-13 of alcohol (**342**) was thus shielded like TES ether, leading to the synthesis of ketone (**343**). When tricyclic-ketone (**343**) was exposed to sodium-azide derivatives [NaN(TMS)_2_], the resulting Na-enolate was treated with Davis reagent (**344**) to obtain the required C-4 stereoisomer (**345**) as a major isomer. The TES group of the trisilylated (**345**) was chemoselectively removed with TFA, and the released C13 alcohol of molecule (**346**) was oxidized to the equivalent ketone of a molecule (**347**) using Dess–Martin. Crotophorbolone (**348**) was produced by deprotecting disilylated (**347**) with a methanolic solution of HCl (Scheme 22) [122]. 

### 5.8. Atropurpuran

In 2009, the Wang group identified the arcutane-type atropurpuran diterpene from Aconitum species [123]. Atropurpuran (**364**) has a greatly constrained tetra-cyclo[5.3.3.0.0]-tridecane with two adjacent bicyclo[2.2.2]octane motifs. The formation of atropurpuran (**364**) displays not only the effective combination of a proficient procedure with chemoselective alterations but also demonstrates the feasibility of contradictory formulations of other structural intermediates (diterpenoid, diterpene alkaloids) [124]. It also has anti-inflammatory activities against a variety of viral infections [125].

The selective *C*-acylated product was obtained by treating commercially available 5-methoxy-tetralone (**349**) with LHMDS [lithium bis-(trimethyl silyl) amide] and pentenoyl chloride. At 78 °C, this 1, 3 diketone was treated with TBAF (tetra butylammonium fluoride) and TMS-EBX (Waser’s reagent **350**), yielding (**351**) α–alkynyl 1,3 diketone, which was intramolecularly given (RCEM) ring-closing enyne metathesis using the Grubbs (II) catalyst (**352**), which quickly formed the spiro[5.5]undecane complex; however, the associated terminal olefin keynote would later assist in the intramolecularly Diels–Alder reaction as the dienophile.

The reaction mixture containing RCEM was cooled to 78 °C before adding boron tribromide to eradicate the *O*-methyl moiety and yielded the desired phenol product (**353**)**.** To enhance the whole effectiveness of synthesis, we added a premixed LiAlH_4_/AlCl_3_ mixture to perform a stereoselective double reduction of the diketone molecule at 1, 3 positions, which was the final transformation in the one-pot reaction involving demethylation, RCEM, and double reduction. At room temperature, compound (**353**) underwent RDOD reaction, inserted two methoxy groups successfully into the phenol ortho site to make the equivalent diene moieties (**355**).

By removing the MeOH, adding mesitylene and BHT (butylated hydroxyl toluene) to the foregoing assortment, and then heating up to 1 h at 160 °C, the diene underwent an IMDA reaction. This one-pot IMDA/RDOD rapid instant synthesis yielded the required pentacyclic (**355**) with tetracyclo[5.3.3.0.0]-tridecane core in a 55% yield. In a 63% yield, pentacyclic compound (**355**) underwent homogeneous hydrogenation with Crabtree’s catalyst (**356**), following a Ley oxidation in the earlier mixture, generating the required diketone (**357**). Cross-coupling reactions of various kinds convert diketone (**357**) into progressive intermediates via (**358**) enol triflate.

The corresponding triol was successfully generated by reductive carbonylative coupling with (**358**) enol triflate. Under DMSO, TFAA, and Et_3_N conditions, two hydroxyl groups of secondary alcohol were quickly oxidized selectively to generate allyl alcohol (**359**). Using one-pot regioselectivity, the Dess–Martin oxidation/hydrogenation process efficiently transformed allylic alcohol (**359**) to aldehyde (**360**).

**Scheme 23:**
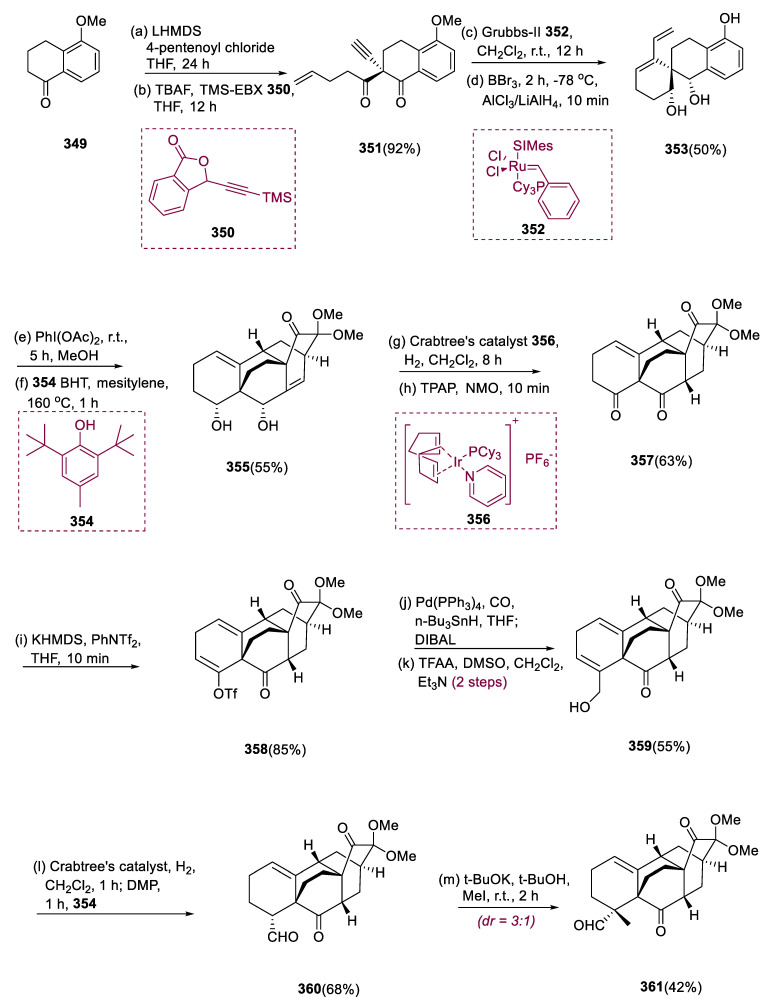

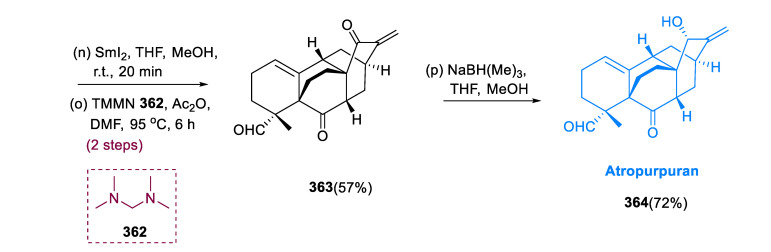
Total synthesis of atropurpuran.

With moderate yield and diastereoselectivity, α-methylation effectively produced the quaternary center of **361** (42%, 3:1). After SmI_2_ activation of demethoxylation of compound (**361**), following α-methylenation of ketone at C-15 site, with TMMN (*N, N*, *N′, N′* tetramethylmethane diamine), and (MeCO)_2_O, enone (**363**) was formed. Atropurpuran (**364**) was obtained through a stereoselective reduction of enone (**363**) with NaC_3_H_10_BO_3_ (sodium trimethoxy borohydride) (Scheme 23) [126].

### 5.9. 1-Hydroxytaxinine

1-Hydroxytaxinine (**394**), derived from *Taxus cuspidata* Japanese yew, is cytotoxic and has human epidermoid KB carcinoma cells and murine leukemia *L1210* cells of IC_50_ values 6.9 and 4.6 mg/mL, respectively [127]. This natural substrate is a component of the taxane diterpenoids family, which comprises over 400 congeners [128]. Many compounds in this class have been tried in clinical trials to treat various malignancies [129].

Using ethylene glycol and CSA, one of the two carbonyl moieties of a molecule (**365**) was protected like di-oxolane of compound (**366**). The remaining ketone of compound (**366**) was α-methylated with LiN(SiMe_3_)_2_/MeI before being transformed into compound (**368**) vinyl iodide via the formation of the hydrazone and treatment with l_2_ in DBN. The Pd^0^-catalyzed Heck coupling between compound (**368**) and (**369**), extended at C-11, resulted in the α, β, γ, δ-unsaturated ester (**370**). The less-substituted diene (**368**) at C-9 olefin was dihydroxylated enantio- and regioselectively using the AD-mix-β to obtain molecule (**371**) at (96% ee).

Methyl ester (**372**) underwent saponification with aqueous lithium hydroxide after the protection of acetonide at the subsequent vicinal diol (**371**). The activated ester was prepared with iBuOCOCl and NMM and converted instantly into α-alkoxy acyl telluride of molecule (**373**) via attacking of the TePh anion formed by (PhTe)_2_ and NaBH_4_. The adduct (**377**) was created through radical coupling between the *A*-ring (**374**) and the *C*-ring (**375**), followed by oxidative olefin regeneration at C-8. 

The treatment of the compound having an *A*-ring (**374**) and two equivalent *C*-rings (**375**) with three equivalents of Et_3_B in C_6_H_6_ at 50 °C open to the air affected the synthesis of the C-8 and C-9 bond, and then DDQ was added to the mixture to yield adduct (**377**) as a major C-9 isomer in a 65% yield. The formation of ethyl radical by O_2_/Et_3_B stimulates the homolytic breakdown of the C-Te bond to produce acyl radical, which undergoes unprompted CO discharge to yield the α-alkoxy A radical (**374**). Following 1, 4-radical addition, the 1, 2-diols of A (**374**), were protected by the acetonide group, redefining the absolute C-9 geometry, as *C*-ring in a molecule (**375**) comes from the opposite side of the hindered C-10 substituent of *A* (**374**).

Following that, Et_3_B captures the resulting radical intermediate to produce the boron enolate B (**381**), and the oxidation of DDQ yields the enone (**384**). Enantiopure molecule (**384**) was achieved by recrystallizing the obtained enone (**377**) in (96% ee). The C8-quaternary center was stereoselectively inserted from the enone (**377**) before the synthesis of a substrate (**378**) for another significant radical reaction by the 1, 4-addition of CH_3_MgBr in the presence of Me_2_S/CuI in C_6_H_5_CH_3_. Alcohol (**378**) was produced via the NaBH_4_ reduction of the C-4 ketone in one step.

The secondary alcohol of molecule (**378**) was then consecutively reacted with Et_3_N, DBU, and MsCl, leading to the synthesis of α, β-unsaturated nitrile (**379**) by removal of mesylate. With diisobutylaluminum hydride, the nitrile (**378**) was reduced to the equivalent imine (**379**) and the subsequent acidic workup effectively hydrolyzed the C-2 imine at C-2 and acetal at C-1 to yield the required ketoaldehyde (**380**). At 50 °C in the presence of pyridine in THF, keto-aldehyde (**380**) was treated with 4 equivalents of TiCl_4_ and 10 equivalents of Zn to form a compound (**381**).

When the diastereomeric mixture was acetylated using DMAP and Ac_2_O, the secondary OH of the molecule (**381**) at C-2 was chemoselectively acetylated over the tertiary-OH at C-1, synthesizing molecule (**382**), yielding 45%. Allylic methylenes of a molecule (**382**) at C-5 and C-13 were oxidized simultaneously with CrO_3_ and 3, 5-dimethyl pyrazole (**383**) to yield bis-enone (**384**). Thus, the addition of AcOH and *p*-toluene sulfonyl hydrazide converted the diketone **384**’s less-hindered C-5 carbonyl group into the sulfonyl hydrazone (**385**), which was treated to NaOAc and catechol borane.

The unprotected top surface of the hydrazone (**385**) underwent 1, 2-reduction, which resulted in the removal of a *p*-tolyl sulfinate. Following the rearrangement of allylic diazene the hydrogen atom was shifted at C-3 from the bottom surface, and the olefin site was altered, resulting in the selective production of the molecule (**386**) (47%), and its epimer C-3-epi-(**386**) in a 21% yield. The dihydroxylation of the disubstituted C-5 olefin of the diene (**386**) proceeded stereoselectively from the bottom site via catalytic OsO_4_ and stoichiometric NMO to give diol (**387**). Using the Yamaguchi reagent system, the less-hindered C-5 OH of diol (**387**) was transformed into the compound (**389**), cinnamoyl ester, while the remaining C-4 hydroxyl group of compound (**389**) underwent oxidation of ketone at C-4, by treating with PCC.

The chemo- and stereoselective nucleophilic addition of MeMgBr to the C-4 ketone (**390**) yielded compound (**391**) without altering the C-13 carbonyl, C-2, and C-5 acyloxy groups. Before dehydration, the acetonide group of C-9 and C-10 diol were substituted for the two acetyl groups in a single pot by successive treatment with CF_3_CO_2_H/MeOH and Ac_2_O. Through the Wagner–Meerwein rearrangement, activation of the C-1 bridgehead hydroxy group (**392**) triggered C-11 migration from C-15 to C-1.

**Scheme 24:**
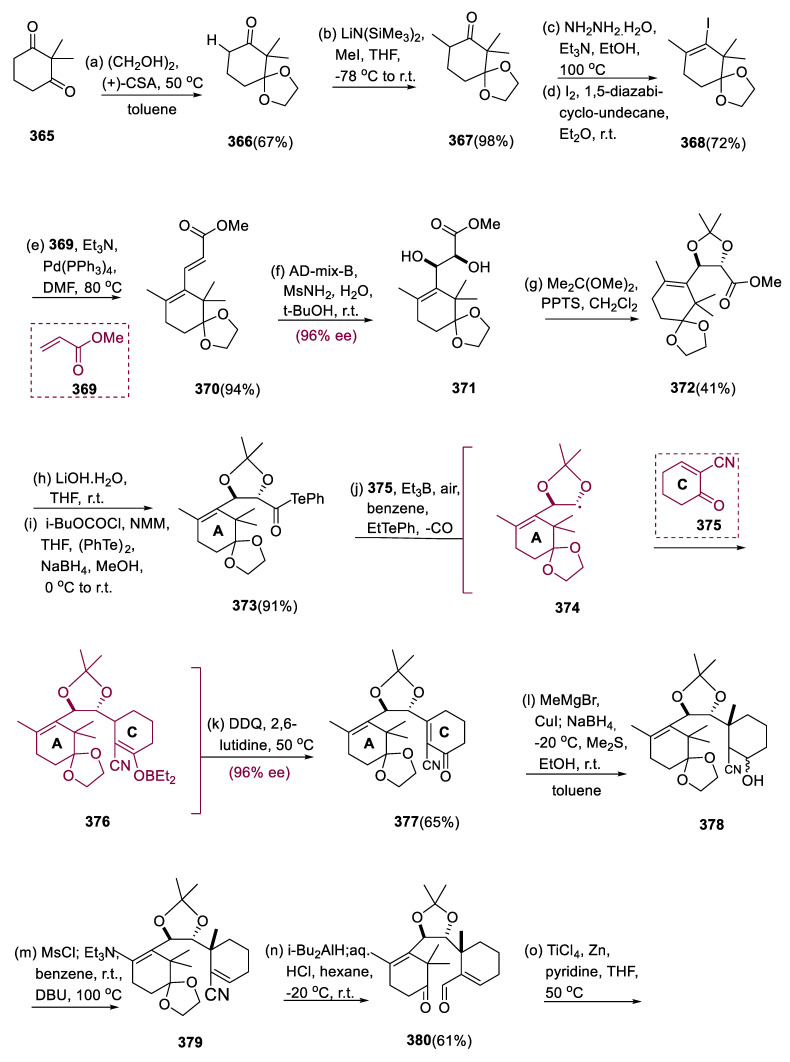

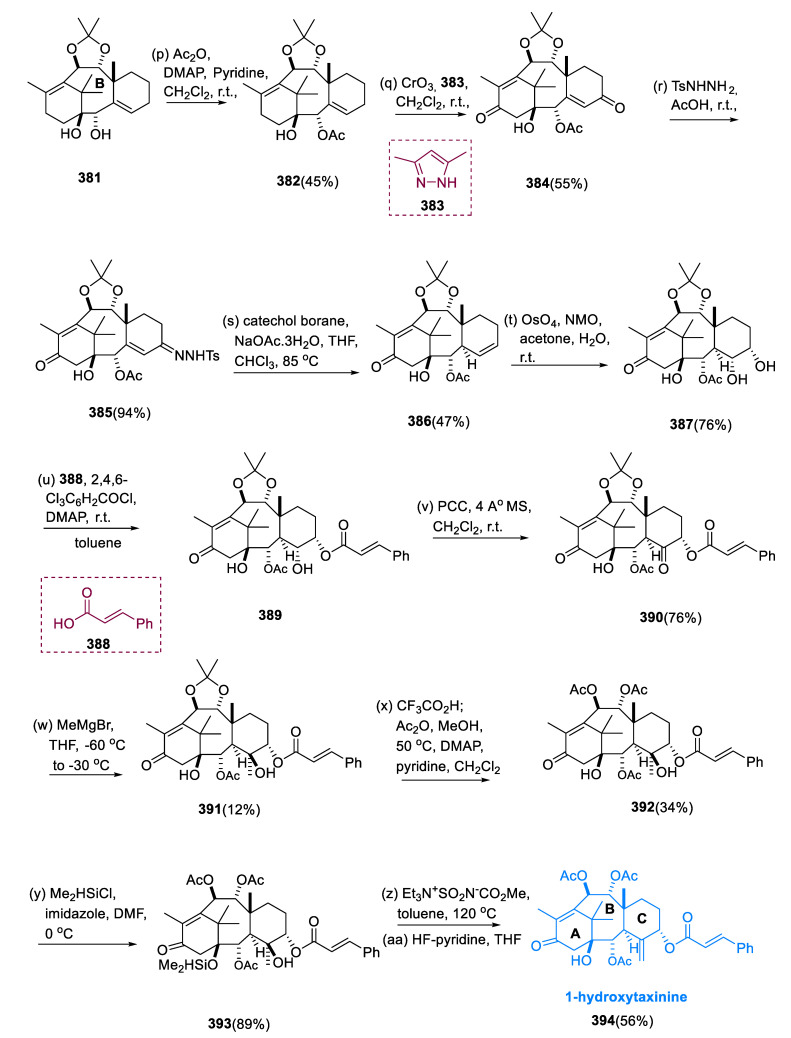
Total synthesis of 1-hydroxytaxinine.

In the presence of Me_2_HSiCl and imidazole, we protected the sterically hindered C-1 OH of a hydroxy group (**392**) as a dimethyl silyl ether, and molecule (**393**) was reacted with the Burgess mixture and then with HF•C_6_H_5_N. This allowed for the synthesis of the desired product (**394**), 1-hydroxytaxinine, in a 56% yield (Scheme 24) [130].

### 5.10. Vinigrol

Hashimoto and colleagues isolated (–)-vinigrol (**413**), a compact, unique, and biologically substantial diterpene, from the fungal strain F-5408, *Virgaria nigra* in 1987 [131]. (–)-vinigrol (**413**) has a 1,5-butanol decahydro naphthalene structure with eight adjacent stereocenters. Significantly, the strained ring system of bicyclo[5.3.1]undecane in (**413**) vinigrol, a novel bridging framework, is found in the terpene taxol [132]. As a result, vinigrol formation is a huge challenge. It has several intriguing pharmacological actions, including the inhibition of human-platelet accumulation having an IC_50_ value is 52 nM, and the inhibition of tumor-necrosis factor (TNF-α) [133].

The starting material (**395**) was prepared in THF with 2-chloro acetyl chloride and LiHMDS, following furanone reduction with DIBAL to generate furan (**396**) with a 40% total yield. Organocatalytic hydroxymethylation of 3-methyl-butanal was enantioselectively followed by Wittig olefination and brominating to generate molecule (**398**).

Compound (**398**) was obtained in a 94% yield by treating chloride (**396**) with a CH_3_MgBr produced from Br^−^ (**397**) in the presence of CuI and THF. Hydroxy methylation of the furan ring in a molecule (**398**) with nBuLi and HCHO yielded intermediate (**399**) in an 82% yield. However, the intermediate (**399**) underwent oxidative rearrangement with TBHP and VO(acac)_2_ in CH_2_Cl_2_, yielding molecule (**400**) in a 92% yield, the starting substrate for the type II intramolecular [5+2] cyclo-addition.

Chemoselective hydrogenation of the C-9 and C-18 olefin in a molecule (**403**) using Wilkinson’s reagent at 1K pressure of H_2_ in toluene, following diastereoselective hydroboration oxidation of the enone with BH_3_•THF in a one-pot step, yielded diol (**404**) with a 71% total yield. Following treatment with five equivalents of 2-iodoxybenzoic acid in DMSO at 80 °C, molecule (**404**) was obtained (30%).

**Scheme 25:**
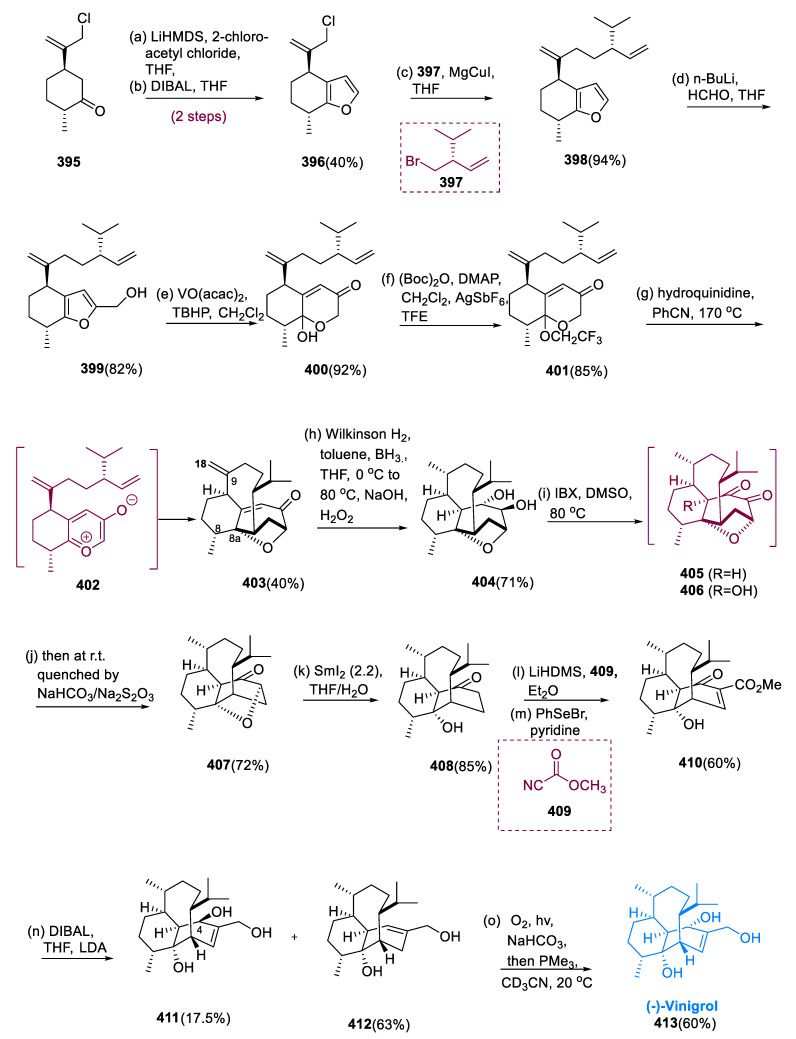
Total synthesis of (–)-vinigrol.

When diol (**404**) was treated with five equivalents of IBX in DMSO at 80 °C for 2 h, quenching with Na_2_S_2_O_3_/NaHCO_3_ at the same time, molecule (**407**) was synthesized in a 72% yield as a major intermediate. Ketone (**408**) was obtained in an 85% yield after treating molecule (**407**) with 2.2 equivalents of SmI_2_ in THF/H_2_O. Ketone (**408**) was reacted with (**409**) methyl cyanoformate (Mander’s reagent) in Et_2_O and LiHDMS, followed by phenyl selenylation with C_6_H_5_SeBr and spontaneous eradication to generate enone ester (**410**) in a 60% yield. Then, in the final step, we attempted to eliminate the ketone and ester groups of molecule (**409**) via chemo- and diastereoselectivity to yield (–)-vinigrol (**413**) (Scheme 25) [134].

### 5.11. Asperolide C

Asperolide C (**427**) is a tetranor labdane diterpenoid isolated from *EN*-*48 Aspergillus wentii*, via a unique asymmetric catalytic polycyclization cascade resembling its biogenesis [135]. The diterpenes having labdane functionality cover a structurally diverse family of natural molecules that are extensively dispersed in marine and terrestrial species [136]. Many of these compounds have significant biological activities, such as antibacterial [137], antimutagenic [138], cytotoxic [139], anti-inflammatory, and analgesic properties [140].

The synthesis of asperolide C (**427**) began with the synthesis of compound (**414**) vinyl ketone, which was prepared in three steps from commercially available *δ*-butyrolactone. Vinyl ketone (**414**) is converted into enol silane (**415**), and the silyl group is exchanged with the triflate (**416**). Under ordinary circumstances, the formation of enol silane (**415**) is impeded by the polymerization of vinyl ketone (**414**). When a molecule containing vinyl ketone (**414**) was added to a premixed solution of tertiary butyl dimethyl silyl chloride and LiHMDS in THF at −78 °C, using HMPA as a cosolvent, the required intermediate was produced in a 95% yield and had remarkable *Z*-selectivity, having a diastereomeric ratio of 95:5.

Under Johnson and Braun conditions, cross-coupling of enol triflate (**416**) and boronic acid (**417**) was attained by using 10 mol% of [Pd(dppf)Cl_2_] as a catalyst in combination with 10 mol% of AsPh_3_ as a co-ligand and Cs_2_CO_3_ as a base to produce the desired product (**418**) in a 62% yield (10:1). Diene (**418**) of terminal olefin was hydroborated with 9-BBN, and trialkyl borane was subjected to Suzuki coupling with vinyl iodide (**419**) to yield polyene in 61%. Re-introducing the re-covered starting substrate resulted in allylic alcohol (**420**) in an 81% overall yield.

Under normal circumstances, a reaction of allylic alcohol (**420**) with 3.2 mol% of [Ir(cod)C_2_] and 12.8 mol% of molecule (**421**) as catalyst precursors and 16 mol% of Zn(OTf)_2_ as a Lewis acid gives decalin (**422**), with remarkable stereoselectivity 9:1 d.r. in a 73% yield. Deprotection of decalin (**422**) was followed by stepwise oxidation of the primary hydroxy group in the production of asperolide C (**427**). The resulting carboxylic acid was reacted with trimethylsilyl diazomethane to give the appropriate methyl ester in a 62% yield.

**Scheme 26:**
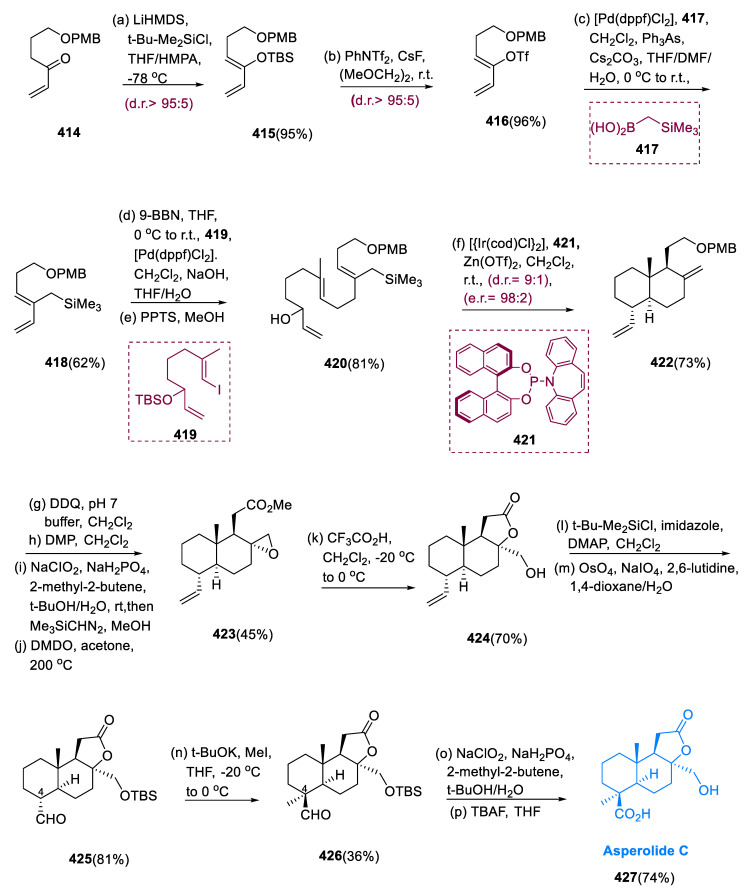
Total synthesis of asperolide C.

The exomethylene group was epoxidized with newly produced DMDO at 200 °C, yielding oxirane (**423**) in a moderate yield. At 0 °C, exposure to trifluoroacetic acid in anhydrous CH_2_Cl_2_ resulted in selective epoxide opening and rapid cyclization to yield lactone (**424**). Lemieux–Johnson oxidation yielded aldehyde (**425**) in an 81% yield after masking the main hydroxy group in (**424**) with a TBS ether. The treatment of a mixture of aldehyde (**425**) in THF at −20 °C with 1.25 equivalents of tBuOK followed by the addition of 1.25 equivalents of iodomethane to 0 °C resulted in aldehyde (**426**) as a single isolable intermediate. The first overall synthesis of asperolide C (**427**) was accomplished by Pinnick oxidation of aldehyde (**426**) to the appropriate carboxylic acid in a 76% yield and the breakdown of the TBS group in a 74% yield (Scheme 26) [141].

### 5.12. Salvinorin A

Salvinorin A (**449**) belongs to neoclerodane diterpene, was isolated from Mexican medicinal herb *Salvia divinorum* [142]. The most potent naturally occurring hallucinogen in humans is neoclerodane diterpene, which is also a robust and highly selective k-opioid receptor (KOR) agonist [143,144]. Salvinorin A (**449**) is a promising new therapeutic target for the treatment of CNS diseases, pain, depression, and drug addiction [145].

The Liebeskind coupling of compound (**428**) vinyl iodide and stannans (**429**) increased the yield of diene (**431**). Furthermore, increasing the IMDA reaction time of the resulting acrylate (**433**) led to a higher overall yield of cycloadduct (**434**). Dihydroxylation of unsaturated lactone (**434**) produced diastereomeric diols (**435**) and (**436**), which had considerably dissimilar reactivity toward NaIO_4_, which need 2 d for complete diol breakdown of the mixture.

Phenyl iodine diacetate (PIDA) significantly decreases the reaction time to 1.5 h with a better yield. Chemoselective Takai olefination of the resultant ketoaldehyde (**437**) yielded *E*-vinyl iodide (**438**), which was then olefinated with sodium salt (**439**) by Horner–Wadsworth–Emmons. During this reaction, substantial epimerization proceeded, resulting in the desired C8 epimer (**440**). With the help of DBU, stability between molecules (**440**) and (**441**) could be efficiently achieved, allowing for the complete conversion of molecule (**440**) into C8 epimer (**441**) before the IMDA phase, which was achieved by the chromatographic fractionation of these enantiomers.

Triene (**443**) was effectively prepared by Stille coupling of a molecule (**441**) with vinyl stannane (**442**) through IMDA, yielding triene (**443**), with a highly diastereoselective translink between dienophile and diene. After heating triene (**443**) for 3.5 d in chlorobenzene with BHT to 200 °C, the required diastereomer (**444**) was isolated in an 87% yield by flash chromatography (94% ds).

**Scheme 27:**
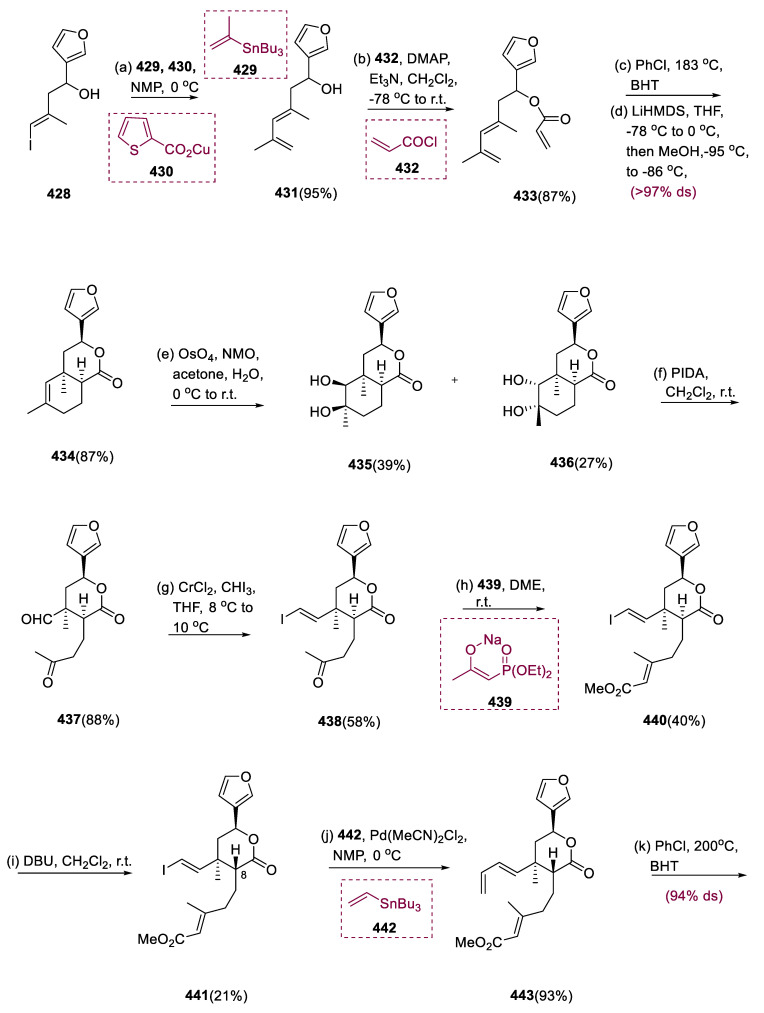

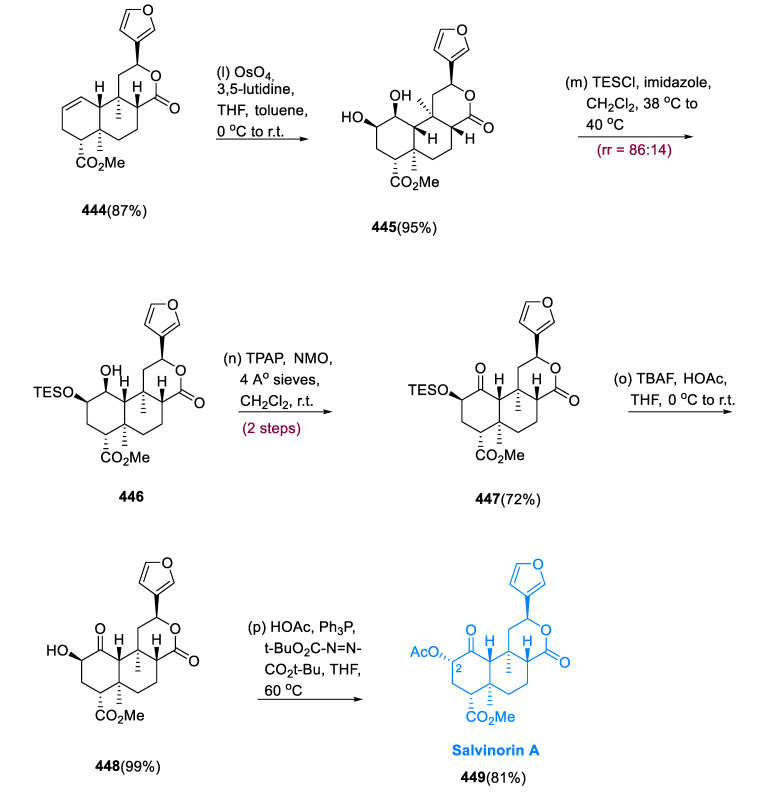
Total synthesis of salvinorin A.

Dihydroxylation of cyclohexene (**444**) from a less sterically hindered surface produced diol (**445**), which was mainly (86:14) converted to the regioisomer (**446**) by silylation with TESCl. Using Ley–Griffith oxidation and flash chromatography, ketone (**447**) was isolated as a single diastereo- and regioisomer from molecule (**446**) in a 72% yield. De-silylation of ketone (**447**) with TBAF, which was buffered with AcOH, resulted in the formation of 2-epi-salvinorin B (**448**). In an 81% yield, the final Mitsunobu inversion of (**448**) 2-epi-salvinorin B with AcOH yielded salvinorin A (**449**) (Scheme 27) [146].

## 6. Triterpenoids

### 6.1. Propindilactone G

Sun and coworkers extracted Propindilactone G (**474**), a novel category of nortriterpenoids from several *Schisandracea* plants [147]. Propindilactone G (473) has a distinct 5/5/7/6/5 pentacyclic core with seven stereocenters, three of which are quaternary centers (C-9, C-10, and C-13). The species is found throughout North America and South-East Asia, and it is utilized in prudent Chinese herb treatments for liver protection and regulating the immune system. Biological tests revealed that these nortriterpenoids have promising anti-HIV potential [148].

Asymmetric Diels–Alder reactions are used to synthesize ester (**453**) from diene (**450**) and dienophile (**451**). The requisite Diels–Alder reaction could be effectively accomplished in Hayashi’s ligand (**452**), resulting in the synthesis of (–)-ester (**453**) in an 88% yield with a high ee of 98%. Furthermore, in two steps, aldehyde (**453**) was added with MeMgBr in Al(Me)_3_ to create alcohol function, which was subsequently oxidized with DMP in NaHCO_3_ and CH_2_Cl_2_ to yield keto ester (**454**) in 74% overall yield.

Keto ester (**454**) treated with MeMgBr yielded a lactone moiety, which was oxidized by treatment with KHMDS in P(OMe)_3_/O_2_ in THF, which was further treated with TESCl, yielding product (**455**) in a 76% total yield. Further treatment of molecule (**455**) with dibromo carbene obtained from CHBr_3_/tBuOK led to the synthesis of dibromide (**456**) as a pair of diastereoisomers (1:1), which was then treated in acetone with AgClO_4_ to provide the cycloheptenone-based vinyl bromide (**457**) in a 57% yield.

Vinyl bromide (**457**) was combined with acetylene-TMS in the presence of i-Pr_2_NH and Pd(PPh_3_)_2_Cl_2_/CuI to produce enone (**458**) in an 88% yield. The treatment of enone (**458**) with (3-methyl but 3 en-1-yl) MgBr^−^ (**459**) in cerium trichloride resulted in enyne (**460**) as a single isomer in an 81% yield. Enyne (**460**) was treated with 0.5 equivalents of Co_2_(CO)_8_ in the presence of celite in toluene under reflux to prepare the cyclopentenone subunit with an all-carbon quaternary stereogenic center. 

The desired product (**461**) was produced in a 67% yield, along with its C-13 diastereoisomer in a 24% yield. When a product (**461**) was treated with silver fluoride, dienone (**462**) was produced in an 85% yield after the silyl groups were removed. The treatment of dienone (**462**) with Pd(OH)_2_/C in the existence of triethylamine at hydrogen balloon pressure triggered a reductive isomerization, yielding dienone derivative (**463**) in a 98% yield. Then, further treatment of dienone (**463**) with *m*-CPBA in CH_2_Cl_2_ yielded epoxide (**464**) in a 73% yield as a single stereoisomer. In the presence of Et_3_N, epoxide (**464**) was first treated with acetic anhydride, and the resultant acetate was reacted with LiHMDS to commence a Dieckmann condensation, yielding lactone (**465**) in a 76% yield.

To obtain the stereo- and chemoselective synthesis of molecule (**466**), intermediate (**465**) was first dehydrated with Martin’s sulfuran, and then the resulting unsaturated lactone (**466**) was subjected to both Pd-catalyzed hydrogenation at the (C-1, C-2) double bond and hydrogenolysis for opening the epoxide ring, yielding product (**467**) in 56% overall yield.

**Scheme 28:**
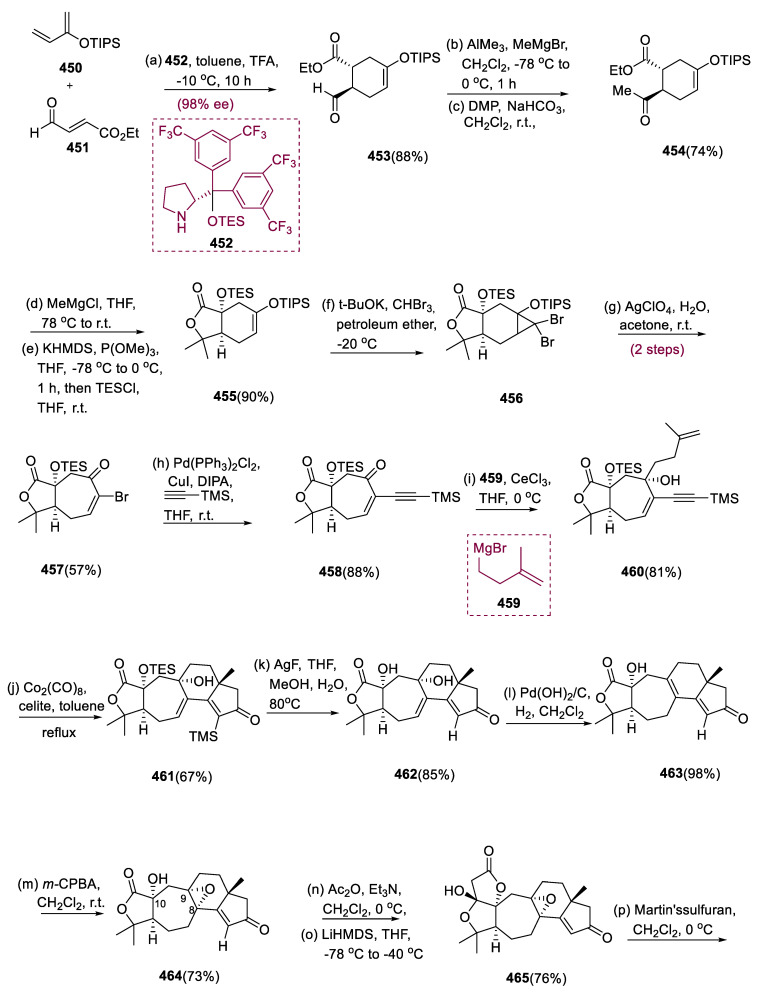

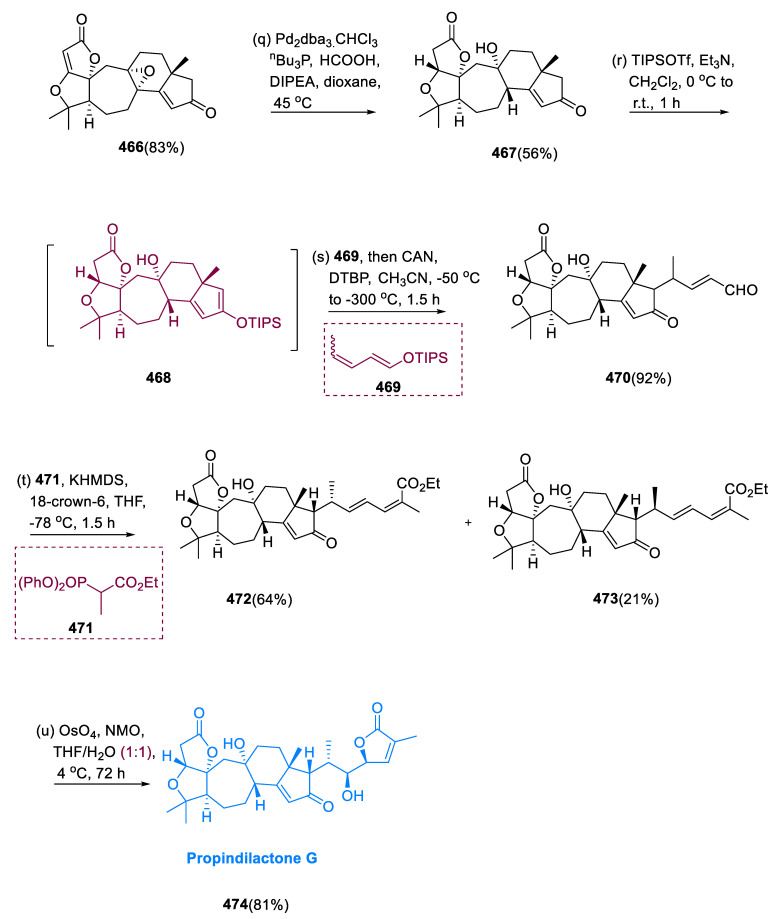
Total synthesis of propindilactone G.

The synthesis of molecule (**470**) can be accomplished through an intermolecular oxidative coupling of conjugated enol silane (**468**) with enol silane A (**469**). We demonstrated that using CAN, an oxidant allowed this coupling to continue smoothly, yielding (**470**) as two pairs of diastereoisomers in a 92% yield. This reaction was taken out in 2, 6 ditertiary butyl pyridine at −50 °C to −300 °C in MeCN, yielding inseparable products (**472**) and (**473**) in 60% yields, which were then reacted with OsO_4_, yielding propindilactone G (**474**) in an 81% yield in the presence of NMMO as a co-oxidant (Scheme 28) [149].

### 6.2. Rhabdastrellic Acid A

The main constituents of the isomalabaricane triterpenoids are rhabdastrellic acid A (**487**) and stelletin E (**488**), which belong to marine natural products that continue to arouse curiosity due to their extremely specific anticancer effects [150]. The nanomolar mean GI_50_ doses of these selective apoptosis inducers inhibiting the NCI-60 tumor cell lines panel are remarkable [151].

Starting with geranyl acetone (**475**), epoxynitrile (**476**) was synthesized via using a van Leusen reductive cyanation of the ketone, following selective epoxidation of the terminal olefin (**475**). Bicyclic ketone (**477**) was made from Ti (III)-mediated reductive-radical polyene cyclization (**476**) and silylation of the resulting alcohol at C-3, yielding insignificant epimers (5:1) at the C-8 site.

Using dichloro methyllithium as a carbon source, the ketone was homologated to an α, β unsaturated aldehyde in (**477**), a diastereoselective reduction of lithium acetylide, simultaneously following protection of the pivalate by completing the synthesis of the major cycloisomerization starting material (**478**) in an 80% yield. When pivalate (**478**) was treated with a cationic gold (**I**) catalyst in Selectfluor^®^, the desired functionalization and annulation occurred with excellent productivity, resulting in the formation of the C-ring-α-fluoro enone as a major stereoisomer.

The separation of α-fluoro hydrazone (**480**) in an 81% yield was made possible by an in situ synthesis of the equivalent *p*–toluene sulfonyl hydrazone. Under usual conditions, the exposure of molecule (**480**) to triethylamine in MeOH spontaneously produced azo alkene (**482**), followed by stereospecific reduction of H_2_ to the appropriate side by retro-ene rearrangement of allylic diazene (**480**). Reductive zirconation and Cu-catalyzed coupling with acetyl AcCl could be used to achieve the required transformation. After extensive optimization, the required C–C bond of deconjugated enone (**483**) was obtained in a 64% yield.

**Scheme 29:**
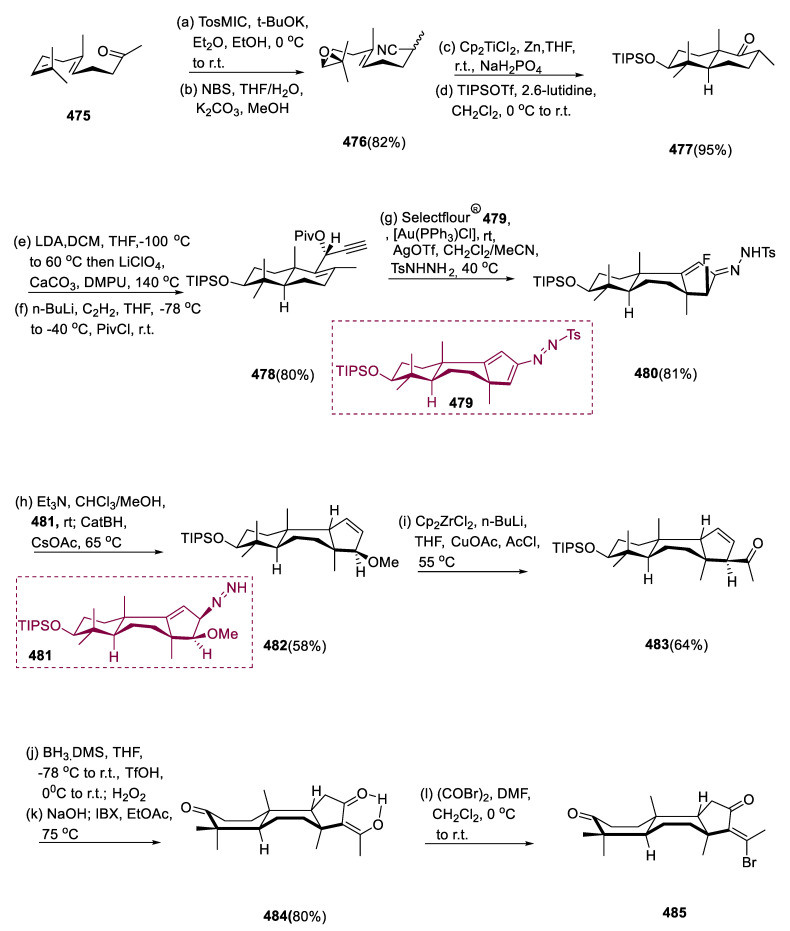

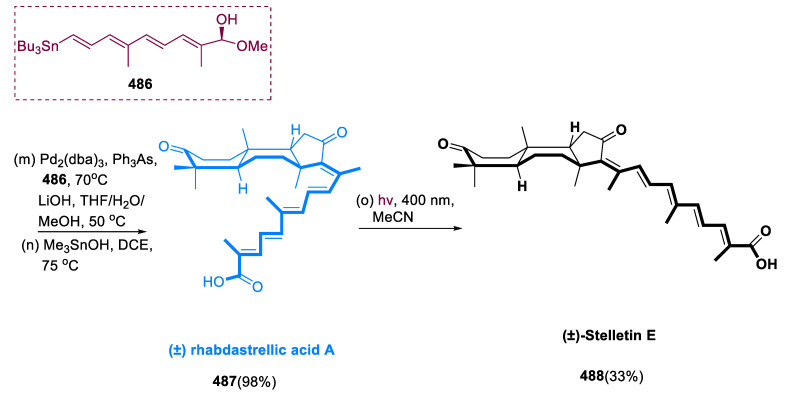
Total synthesis of (±)-rhabdastrellic acid A.

Triketone (**484**) was produced as a single constitutional isomer via relay hydroboration from the ketone, followed simultaneously by deprotection of the silyl moiety with CF_3_COOH and two-fold total oxidation. Bromination using the Vilsmeier reagent produced electrophile (**485**) as a single geometrical and constitutional isomer. Stille coupling of vinyl bromide with tetraenylstannane (**486**) resulted in a 45% overall yield, with rhabdastrellic acid A having methyl ester from triketone (**484**), with the isomeric methyl ester of stelletin E (**488**) in an (8:1) ratio. After saponification with trimethyl tin hydroxide, the desired product (±)-rhabdastrellic acid (**487**) was achieved with a 98% yield (Scheme 29) [152].

### 6.3. Asiaticoside

Asiaticoside (**497**) is an ursane-type triterpene glycoside derived from the tropical plant *Centella asiatica* (L.), sometimes known as Indian pennywort or Gotu kola. It has been used as a cure-all since prehistoric times, but most notably for the treatment of skin diseases and dermatoses such as burns, excoriations, and hypertrophic scars [153]. Asiaticoside may promote collagen synthesis by activating the Smad pathway or inhibiting glycogen-phosphorylase [154,155].

The 28-carboxyl group of a molecule ursolic acid (**489**) was acetylated with benzoyl bromide in the presence of n-Bu_4_NBr and K_2_CO_3_, succeeded by oxidation with Dess–Martin and oxime formation (H_2_NOH•HCl, NaOAc, MeOH, CH_2_Cl_2_) to provide molecule (**490**) in an 87% yield (three steps). By following Baldwin’s approach, we added NaOAc and Na_2_PdCl_4_ to a molecule (**490**) in AcOH solution at r.t. under argon, stirred for 3 d. The Pd^2+^ ursane oxime C-23 angular methyl composite resulted in a yellow solid. The C-23 Pd-linkage was oxidized with lead tetraacetate following reduction with NaBH_4_ to obtain the desired 23-*O*-acetyl-3-acetoxy oxime (**491**) in a 66% yield after acetylation [(CH_3_CO)_2_CO, DMAP, Et_3_N] (over three steps).

The acetyl groups in molecule (**491**) were removed in the presence of K_2_CO_3_ in methanol, and the resultant oximes were efficiently transformed into a 3-carbonyl function by TiCl_3_•HCl [NH_4_OAc/THF/H_2_O]. Then, in MeOH/CH_2_Cl_2_ at 0 °C, we employed *m*-chloro peroxybenzoic acid and H_2_SO_4_ as a catalyst to hydroxylate the ketone and generate the required 2α-hydroxyketone (**492**) in a remarkable yield (82%). Following the reduction in ketone (**492**) under various conditions, including NaBH(OAc)_3_/THF, NaBH_4_/THF, and NaBH(OMe)_3_/MeOH, in HOAc/MeCN, to generate the desired 3-epi-**493** (3-*β*-OH) and **493** (3-α-OH) with good diastereoselectivity (10:1) resulted in an 81% yield.

**Scheme 30:**
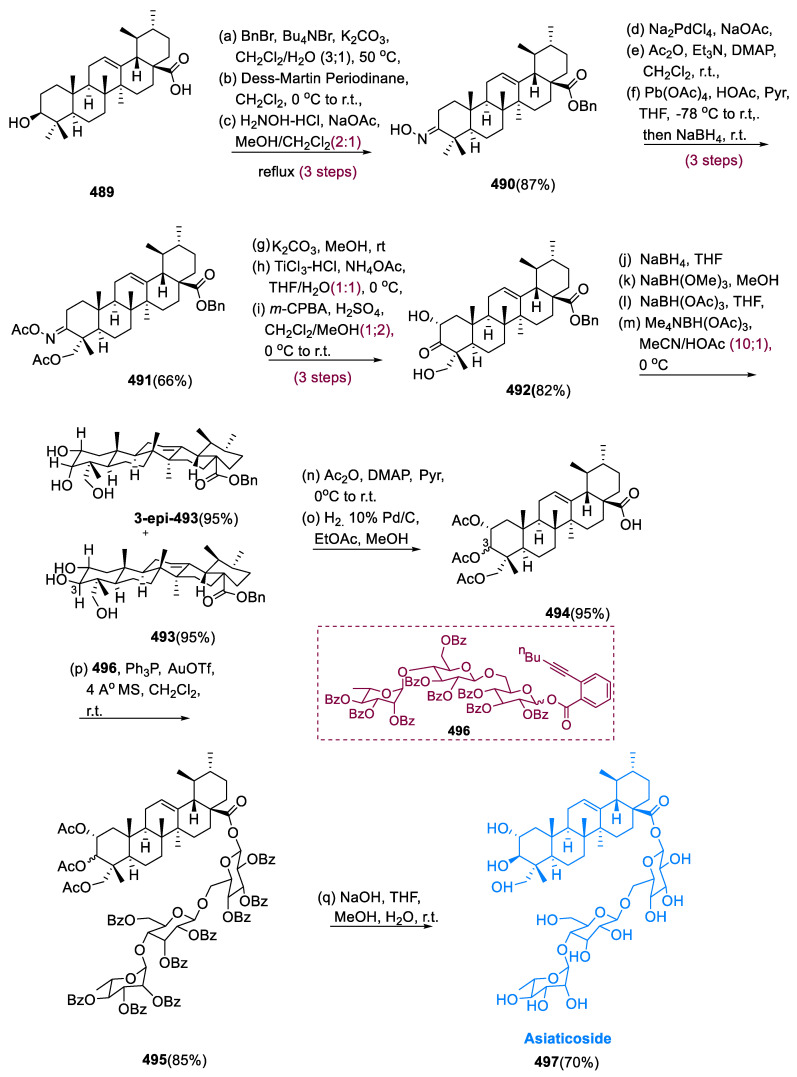
Total synthesis of asiaticoside.

Then, the three hydroxyl functions of a molecule (**493**) underwent acetyl protection in the presence of Ac_2_O, pyridine, and DMAP at ambient temperature, and the 28-benzyl ester was hydrogenolyzed with the support of Pd/C, H_2_, MeOH, and EtOAc at r.t. to yield the desired aglycone (**494**) (95% over two steps). The 3-*epi*-(**495**) derivative was synthesized in the same way, commencing with 3-*epi*-(**493**). Thus, typical AuI-catalyzed glycosylation, involving 0.1 equivalents of AuOTf, Ph_3_P, and CH_2_Cl_2_ at r.t., leading to the condensation of acid (**494**) with orthohexynyl benzoate (**496**), yielded triterpene trisaccharide 3-*epi*-(**495**) in an 85% yield. Then finally, the benzoyl and acetyl groups of (**495**) were eliminated with sodium hydroxide, yielding 70% asiaticoside (**497**) (Scheme 30) [156]. 

### 6.4. Schiglautone A

Ruan and coworkers reported schiglautone A (**524**), which has a new skeleton, in 2011 after identifying novel triterpenoids as possible therapeutic approaches from *Schisandra glaucescens* [157]. The structure of compound (**524**) is comprised of a cyclohexyl-fused bicyclo[6.4.1]tridecane carbon skeleton with a rare bridgehead δ-alkene and six chiral carbons, three of which are quaternary [158].

Schisandraceae plants are mostly utilized in conventional Chinese medicine to treat several ailments, including cough, chronic diarrhea, premature ejaculation, and drowsiness [159,160]. Schisandra triterpenoids have gained increasing consideration from the synthetic community because of their unique framework and essential biological properties, leading to elegant syntheses of their scaffolds and target complexes [161,162].

Over two processes, the epoxypolyprene (**499**/**500**) was synthesized in a 45% yield by regioselective transnerolidol (**498**) via region-selective epoxidation and acetylation. Under the reaction conditions, the titanium (III)-catalyzed 6/7-endo-trig radical cyclization of the molecule (**499**/**500**) yielded the required transfused [6,7]bicycle (**501**) in 50% overall yield.

The effectiveness of this cyclization was greatly increased by changing to carbonate (**502**). Because of the steric hindrance of the angular Me moiety at C-19, the successive epoxidation happened with perfect face selectivity, yielding molecule (**503**) in 86% as a major diastereomer. Following Ley–Griffith oxidation, Meerwein–Ponndorf–Verley reduction was used to invert the configuration at C-3, leading to the formation of an alcohol mixture in 72% total yield preferring the axial isomer. The unification of the benzyl ether (**503**) and methyl acrylate progressed easily utilizing Gansauer’s modified technique [0.5 equivalents of Cp_2_TiCl_2_, 2 equivalents of Zn, 2.5 equivalents of 2, 4, 6-collidine•HCl] for reductive epoxidation, yielding the homologated lactone (**504**) in a 91% yield. Following reduction by LiAlH_4_, Swern conditions were used to produce simultaneous oxidation of the two resulting hydroxyl functionalities, yielding ketoaldehyde via an Et_3_N-facilitated breakdown of the di-alkoxy sulfonium intermediate.

Following chemoselective Grignard addition and silylation, molecule (**505**) was obtained as insignificant mixtures (1:1) at C-17 in a 67% yield (two steps). Instead, the *O*-allylation intermediate (**507**) was synthesized by treating with allyl iodide and LiHMDS at −40 °C and then undergoing Claisen rearrangement at 120 °C to provide the transconfigured diene at C-9 and C-14 in an 85% yield. The expected tricyclic product (**508**) was obtained in a 65% yield with 15mol% of Grubbs (II) catalyst and stoichiometric concentrations of tetra-fluorobenzoquinone for oxidation of the ruthenium hydride complexes.

The 1, 2-addition by MeLi and Dauben–Michno oxidation of the resulting tertiary-allylic alcohol yielded molecule (**509**) in a 60% yield, followed by Jones oxidation. At the convex side of the C-13 and C-17 double bond, the vinyl cuprate underwent Michael addition, produced from the organo lithium (**511**). The direct usage of a secondary alkyl cuprate generated from a Grignard molecule (**512**) resulted in the synthesis of the molecule (**513**) and its *C*-20 epimer with a combined yield of 72%. Through the centrality of the cyclic chromate ester (**514**), PCC oxidants yielded the aldehyde (**515**) in a 68% yield. The *Z*-configured alkene (**518**) was produced in an 84% yield as a sole isomer by the Horner–Wadsworth reaction of molecule (**515**) with (**517**), catalyzed by KHMDS/18 C-6 (**516**). The *Z*-configured alkene (**518**) was initially treated at 78 °C with methyl acrylate, ICl, and LiHMDS to produce α-iodoketone in situ, which was then used to implant the bridgehead alkene.

**Scheme 31:**
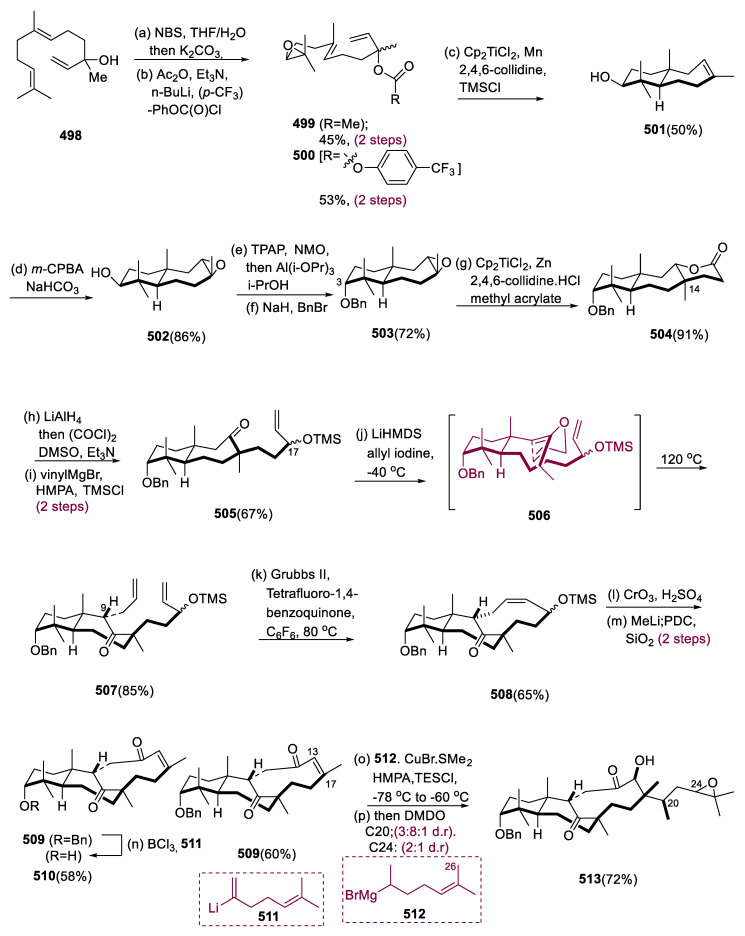

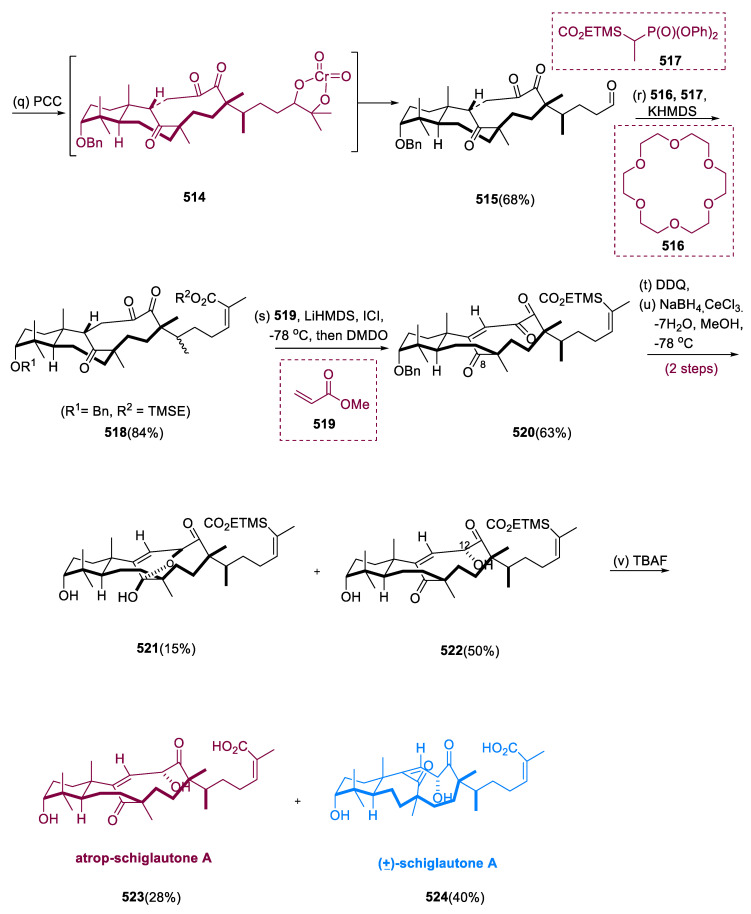
Total synthesis of (±)-schiglautone A.

By adding DMDO solution to the iodine atom, it can be oxidized to create α-iodoso functionality, which then undergoes in situ elimination to provide molecule (**520**) in a 63% yield. After removing the benzyl group at *C*-3 with DDQ, the ketone at *C*-12 is regio- and diastereoselectively reduced in the presence of the other two ketones at *C*-8 and *C*-13. We discovered that the necessary α-alcohol (**521**) was generated as a substantial diastereomer (15%) only under Luche conditions at 78 °C, and molecule (**522**), yielding 50%, respectively. The aromatization of molecule (**521**) was caused by a further reduction in molecule (**522**) on C-13 ketone followed by vinylogous Grob fragmentation. Desilylation of alcohol (**522**) with TBAF yielded (**523**) and (**524**) in a 78% yield. (±)-atrop-Schiglautone A (**523**) is a diastereomeric atropisomer of (±)-schiglautone A (**524**) (Scheme 31) [163]. 

### 6.5. Walsucochins B

Walsucochins B (**543**) is a *C*_24_ nortriterpenoid with a phenyl acetylene group joined to a five-membered contracted circle. Yue and colleagues extracted it in 2007 from the twigs and leaves of *Cochinchinensis Walsura* [164]. Walsucochin B (**543**) contains a 6/5/6/6 attached ring framework with four continuous stereocenters (including two quaternary centers) and a chiral hydroxyl functional moiety. The cell protection effect of novel *C*_24_ nortriterpenoids in contrast to H_2_O_2_-prompted PC1_2_ cell destruction is substantial.

Bromination of commercially available 2,3-dimethyl anisole (**525**) yielded the phenyl bromide (526) in a 98% yield, which was further treated with n-BuLi and also treated with ethylene oxide at 78 °C in THF to obtain the phenyl ethanol (**527**). Over two processes, the main hydroxyl group in phenyl ethanol (**527**) was oxidized with DMP, following Wittig olefination ylide of methyl propionate (**528**), to generate *α, β*-unsaturated ester (**529**) with high stereoselectivity (99:1). The allylic alcohol (**530**) was produced in good yield after the reduction of α, β-unsaturated ester (**529**) with DIBAL-H.

Allylic alcohol (**530**) undergoes oxidation with DMP to yield an unstable, *α, β*-unsaturated aldehyde. The aldehyde was promptly reacted with 1, 3-propane dithiol in the presence of Et_2_O•BF_3_ at 0 °C, yielding dithiane (**531**) in two steps in an 83% yield. The reaction of allylic bromide (**532**) with the organolithium generated from the reaction of dithiane (**531**) with n-BuLi resulted in the required polyolefin epoxide (**533**) in an 86% yield. Following dedithianation of molecule (**533**) with CaCO_3_ and I_2_ at 0 °C gives the α, β-unsaturated ketone (**534**) in an 82% yield, while the epoxy functional moiety was retained. The alcohol (**536**) was obtained in a 71% yield after reduction of ketone (**534**) asymmetrically with intermediate (**535**), *R*-CBS mixture. In a 98% yield, acetylation happened for the protection of alcohol (**536**), to give the necessary main cyclization intermediate (**537**) in the (1:4) mixture. The inseparable diastereomeric combination of a molecule (**537**) could be transformed to the respective core tetracyclic skeleton (**538**) as a single diastereoisomer in a 62% yield by cationic polyolefin cyclization in dichloromethane in DEAC at 78 °C for 10 h. TBSOTf was used to protect alcohol (**538**), and ether, leading to synthesized molecule (**539**) in a 96% yield. The radical bromination of the *C*-20 methyl of the molecule (**539**), with NBS and AIBN in carbon tetrachloride at reflux, following oxidation with DMSO, yielded intermediate (**540**) in a 73% yield (two processes). In a one-pot Gilbert–Seyferth homologation/hydrolysis sequence, (MeO)_2_P(O)CN_2_C(O)-CH_3_ (**541**) and K_2_CO_3_ in methanol transform aldehyde (**540**) to phenyl acetylene and the MeCO function was also eliminated to provide alcohol (**542**) in a 76% yield.

**Scheme 32:**
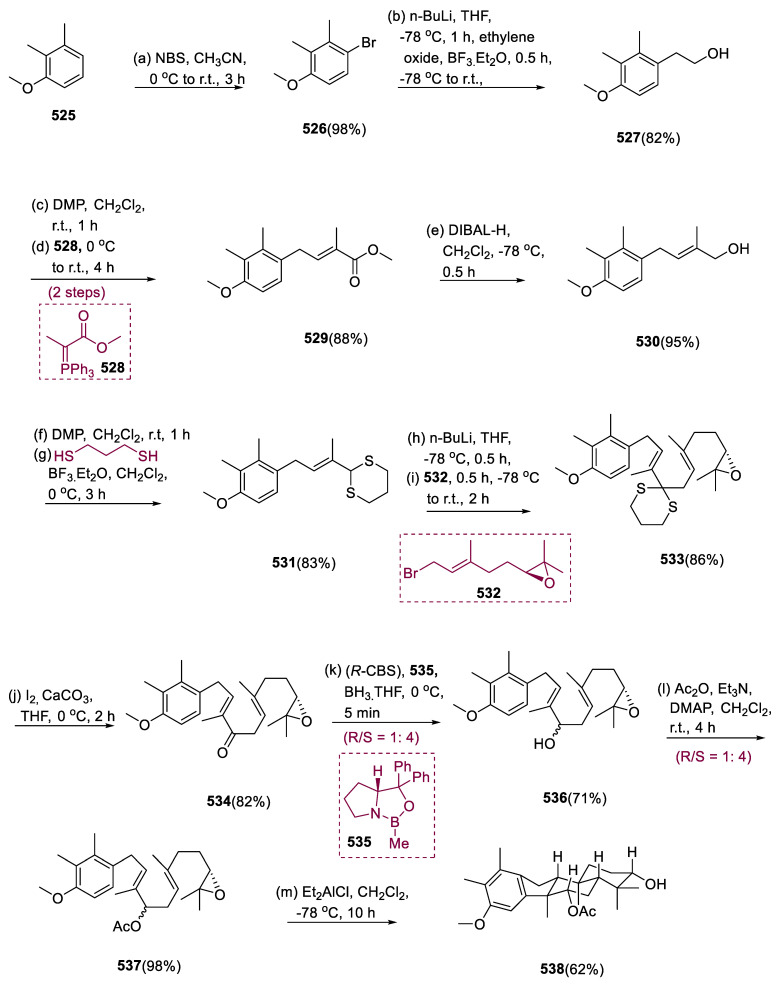

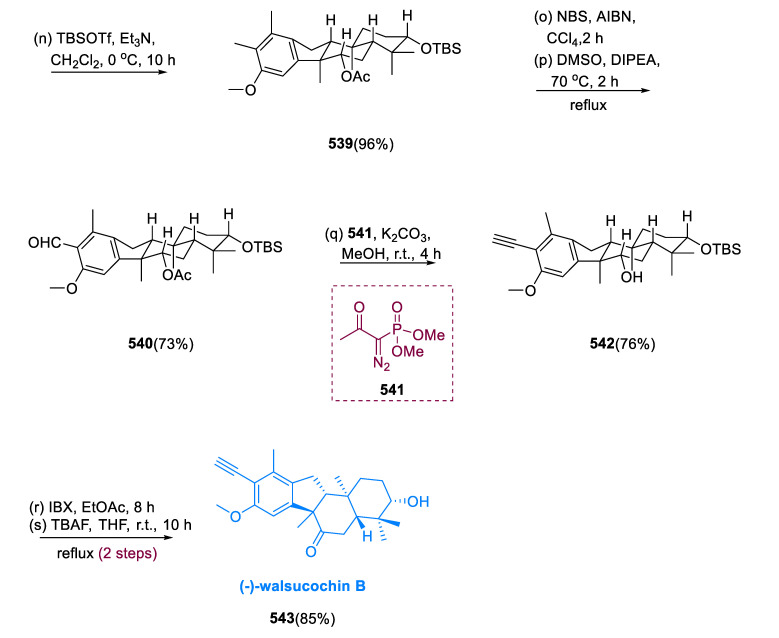
Total synthesis of (–)-walsucochin B.

Finally, the first enantioselective total synthesis of (–)-walsucochin B (**543**) was completed by oxidation of alcohol (**542**) via IBX to the appropriate ketone and desilylation with tetra-butylammonium fluoride (Scheme 32) [165]. 

### 6.6. Rubriflordilactone A

Sun and coworkers indicated the isolation of rubriflordilactone A (**566**) from *Schisandra rubriflora* in 2006, which has a long history of use in Chinese herbal medicine and has promising anti-HIV activity. Rubriflordilactone A (**566**) is a triterpenoid, having a heptacyclic framework with a multisubstituted arene motif [166].

Oxazaborolidine (**545**) and BH_3_•THF of 2-iodo 2-cyclopentenone (**544**) yielded the appropriate alcohol (99% ee), where Sonogashira coupled with acetylene TMS to obtain molecule (**546**) in an overall 81% yield. This alcohol was treated to the 2-nitrophenol and CH_3_CH_2_C(OMe)_3_ following Claisen–Johnson rearrangement to yield Me ester (**547**). Saponification and desilylation happened after treatment with aq. LiOH, yielding acid (**548**) in a 93% yield. When this molecule was exposed to NIS/NaHCO_3_, that exhibited stereo-specific iodolactonization, yielding *E*-alkene (**5499**) in a 52% yield. An asymmetric Cu-catalyzed Diels–Alder reaction was used to create the optically energetic element (**553**) from dienophile (**550**) and diene (**551**). In the presence of bis-oxazoline ligand (**552**) and Cu(OTf)_2_, the cycloaddition proceeded easily, yielding compound (**553**) in an 80% yield. The oxazolidinone moiety was replaced with organolithium, following Pd-catalyzed Me coupling with the thio-ester[*S*-Phos, MeZnI, Pd_2_(dba)_3_], generating ketone (**554**) with high productivity. Ketone (**554**) was exposed to a five-step method proposed by Yang et al. for their efficient formation of enantiomeric schindilactone A to obtain molecule (**556**) bromoenone, through lactone (**555**). The Enone olefin was saturated by Pd/C, H_2_ hydrogenation, while also reducing the resultant bromide, yielding the required cycloheptanone in a 97% yield, which was subsequently regioselectively transformed to triflate (**557**) (PhNTf_2_, LiHMDS) in a 89% yield. The remarkable selectivity could also be attributed to the steric difference between the carbonyl’s α and α′ locations. Acetate (**558**) was obtained by replacing TES with Sc(OTf)_3,_ and Ac_2_O, in a 96% yield. Involving Dieckmann condensation with LiHMDS intramolecularly yielded a hemiketal in a 93% yield and the following deoxygenation (Et_3_SiH, BF_3_•OEt_2_) yielded tricycle (**559**) in a 65% yield. Stannylation [Me_3_SnSnMe_3_, Pd(PPh_3_)_4,_ LiCl] of tricycle (**559**) yielded molecule (**560**) in a 75% yield, opening the way for segment (**561**) cross-coupling. Pd(0) and CuTC catalytic arrangements efficiently accelerated the coupling of molecules (**561, 560**) to generate triene (**562**) in a 96% yield. The *E*-configuration of the double bond at C-11, C-12 was carefully preserved during this moderate and fast coupling, which is critical for the subsequent cyclization.

**Scheme 33:**
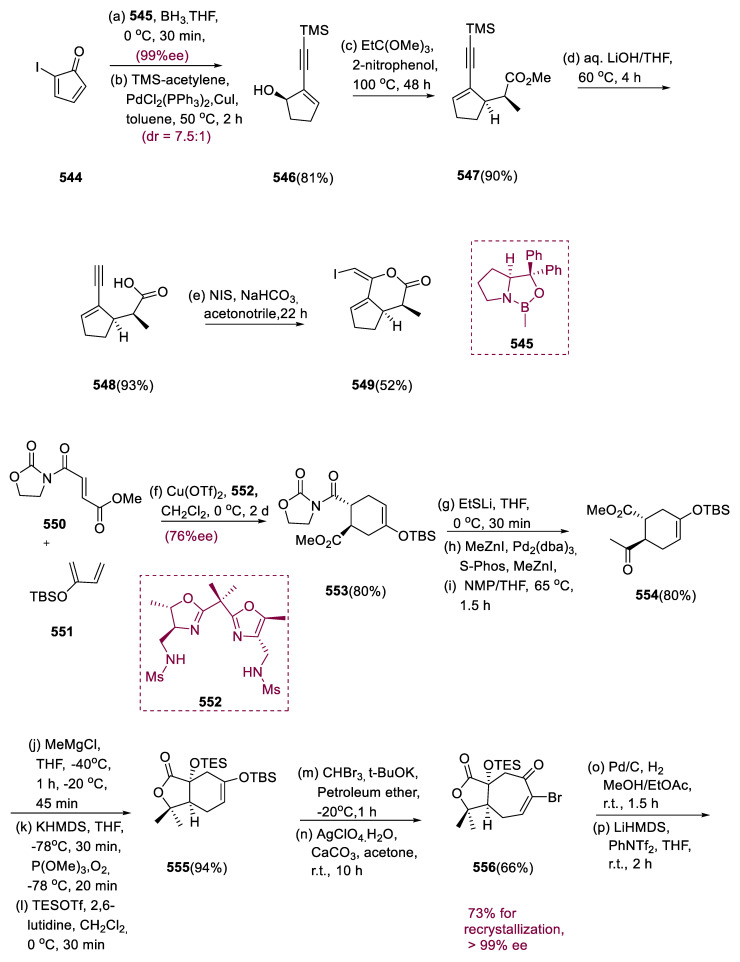

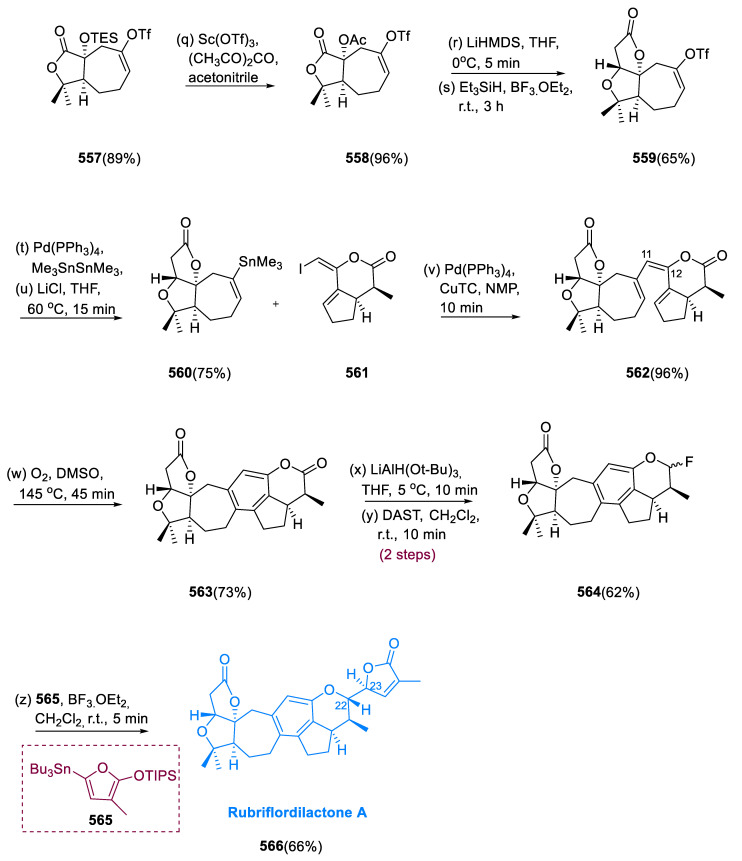
Total synthesis of rubriflordilactone A.

Heating triene (**562**) in dimethyl sulfoxide at 145 °C in an air environment influenced the 6π electrocyclization and aromatization in one step, yielding arene (**563**) in a 73% yield. After aqueous workup, arene (**563**) was treated with hindered LiAlH(Ot-Bu)_3_ to effectively separate the two carbonyl lactones, yielding a combination of its ring-opened aldehyde form and six-membered lactol. When the aldehyde/lactol mixture was exposed to Et_2_NSF_3_, the yielded fluoride (**564**) in 62% (total yield from **563**) was produced as a stimulated “donor” for the following *C*-glycosylation. Fluoride (**564**) was discovered to be unreactive, contrary to siloxy furan; only allyl tri-methyl silane may attack it after BF_3_•OEt_2_ activation. In the presence of boron trifluoride etherate, this stannane (**565**) smoothly interacted with fluoride (**564**) to generate rubriflordilactone A (**566**) in a 66% yield as a major identifiable diastereomer (Scheme 33) [167].

## 7. Sesterterpenoids

### 7.1. Astellatol

Astellatol (**589**) was extracted from *Syn. A. variecolor* (*Aspergillus stellatus*) in 1989 [168]. It contains a highly congested pentacycle having a unique bicyclo[4.1.1]octane, a cyclobutane having 2 quaternary centers, 10 stereocenters, and an exomethylene group. Astellatol (**589**) belongs to isopropyl trans-hydrindane sesterterpenoids, having miscellaneous biological activities such as antimicrobial, cytotoxic, anti-inflammatory, and anticarcinogenic activities [169].

Chiral synthon (**567**) undergoes alkylation with the homoallylic iodide (**568**) giving compound (**569**), which was further reacted by the lithiated derivative of methoxy propadiene (**570**) and then underwent protection and hydrolyzation to obtain the desired product (**571**). The following RCM reaction of compound (**571**) easily gave the desired 5, 7-bicyclic enone using the Grubbs catalyst. Hydrolysis of the TMS ether (**572**) under acidic conditions was treated with TBAF prompting an α-ketol rearrangement to synthesize the 6, 6-bicyclic structure as the predominant intermediate. The tertiary hydroxy group of a molecule (**572**) was then eradicated by a SmI_2_ accelerated α-deoxygenation to furnish (**574/575**). The C-17 epimer of (**574**) altered into compound (**575**) by NaOMe prompted epimerization, which further enhanced the overall efficacy. α-alkylation of compound (**575**) under methallyl bromide/LDA conditions exclusively synthesized the desired epimer (**576**). Ketone (**576**) was reacted with propargyl MgBr with a catalytic amount of mercuric chloride, giving propargyl-substituted lactone by Sonogashira coupling between the 2-bromo propene and alkyne derivatives under room temperature to synthesize substrate (**577**). Substrate (**577**) cyclized smoothly to give compound (**578**) having (4:1 dr) mixture at C-7. Compound (**578**) was further treated with DBU/HMPA and unveiled the afforded dienone with the free carboxylic acid functionality, which then underwent esterification to yield the ester (**579**). Intermediate (**579**) having ketone and ester motifs was reduced and reoxidized to yield the resultant aldehyde (**580**), which was further succeeded to the reductive addition of 1,6-radical under SmI_2_/MeOH/HMPA to provide a separable mixture of regioisomers (**581, 582**) in 22% and 44% yields. Under various hydrogenation circumstances, compounds (**581**) and (**582**) changed into intermediate (**583**). Successive Ley oxidation of intermediate (**583**) provided the diketone (**584**) in 98% productivity.

**Scheme 34:**
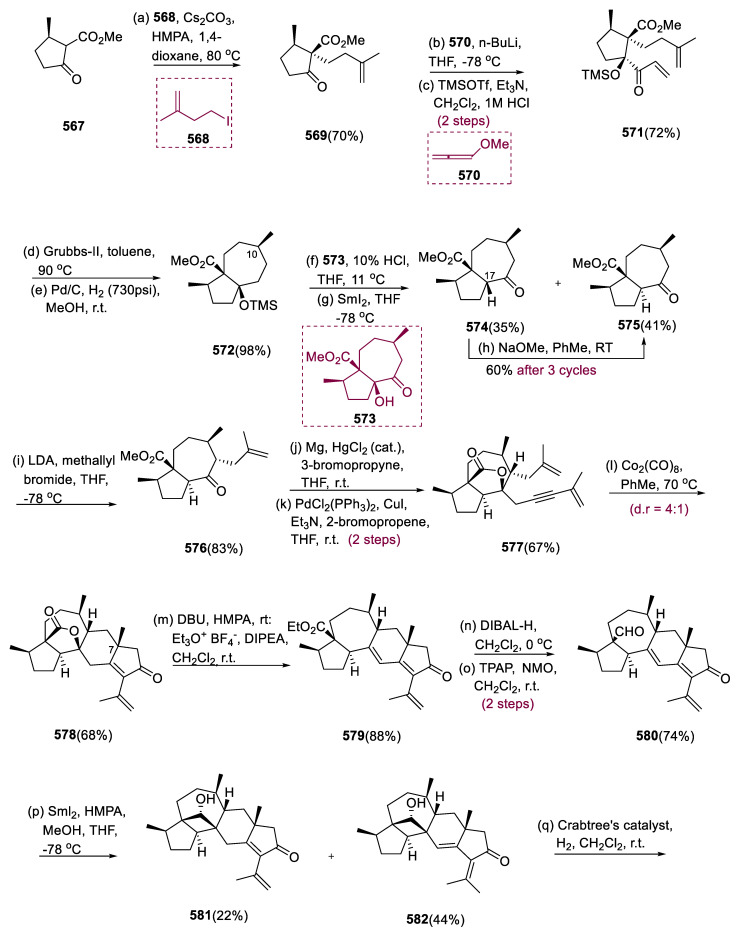

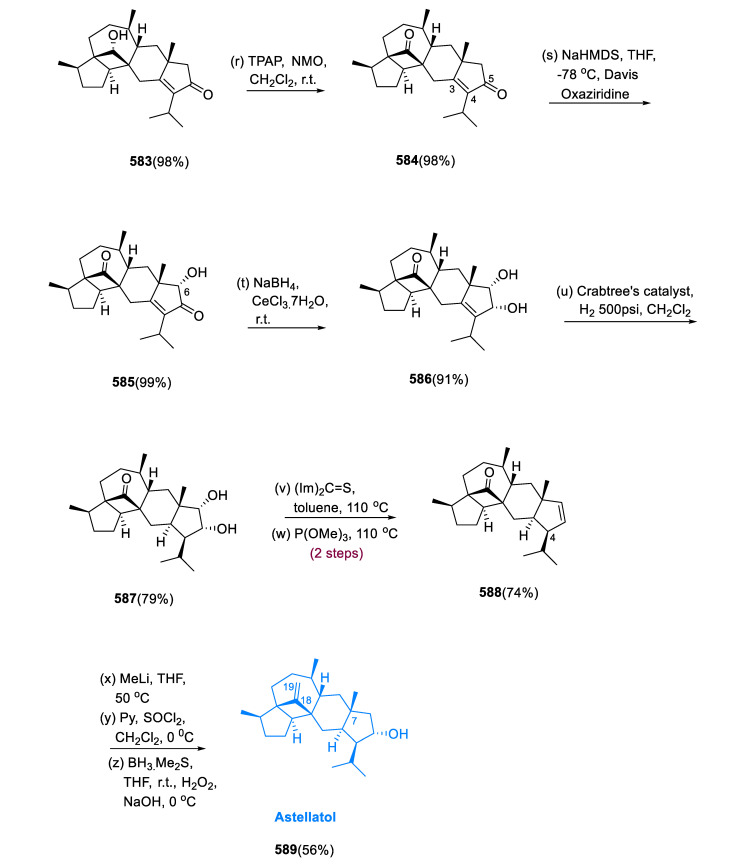
Total synthesis of astellatol.

Fast removal of the C-5 hydroxy group, a Davis α-hydroxylation of diketone (**584**) installed an extra C-6 hydroxy group on the cyclopentanone skeleton to give (**585**). The successive reduction delivered the cis-diol (**587**). Homogeneous reduction of cis-diol (**586**) using CrabtreeQs catalyst efficiently yielded the desired trans-hydrindane (**587**) in 79%. Corey–Winter settings successfully transformed the cis-diol (**587**) via alkene (**586**). Treatment of (**588**) with MeLi at 50 °C gave the tertiary alcohol-involving eradication under Et_3_N and SOCl_2_ to deliver the exomethylene moiety on the cyclobutane skeleton. Then, it later underwent hydroboration–oxidation, synthesizing the astellatol (**589**) as the preferred C-5 regioisomer in a 56% yield (Scheme 34) [170].

### 7.2. Nitidasin

Nitidasin (**602**) is isolated from *Gentianella alborosea* and *Gentianella nitida*, as a remedy against hypertension, diabetes, and hepatitis [171]. Nitidasin (**602**) belongs to the sesterterpenoid having an oxygenated trans-hydrindane scaffold, the five-membered rings that bear an isopropyl motif that is cis to an angular CH_3_ moiety. (+)-Nitidasin (**602**) contains YW-3548 and YW-3699, which are two antagonists of GPI-anchor mammalian biosynthesis [172].

Hydrindane protection of molecule (**590**) gave the analogous SEM ether (**591**) which further underwent Pd-catalyzed allylation giving hydrindanone (**593**). Disclosure of ketone (**593**) to K-Selectride gives rise to diastereoselective reduction of the carbonyl functionalities. After the Lemieux–Johnson breakdown of the terminal alkene and Cr-accelerated oxidation of the molecule lactol to lactone (**594**) for the insertion of the C-3 methyl group, the reaction of the Li-enolate with methyl iodide gives rise to a major diastereomer (**595**). Lactone (**595**) undergoes reduction with LiAlH_4_, following silylation and chemoselective oxidation under optimized Swern conditions, yielding a competent approach to aldehyde (**596**).

**Scheme 35:**
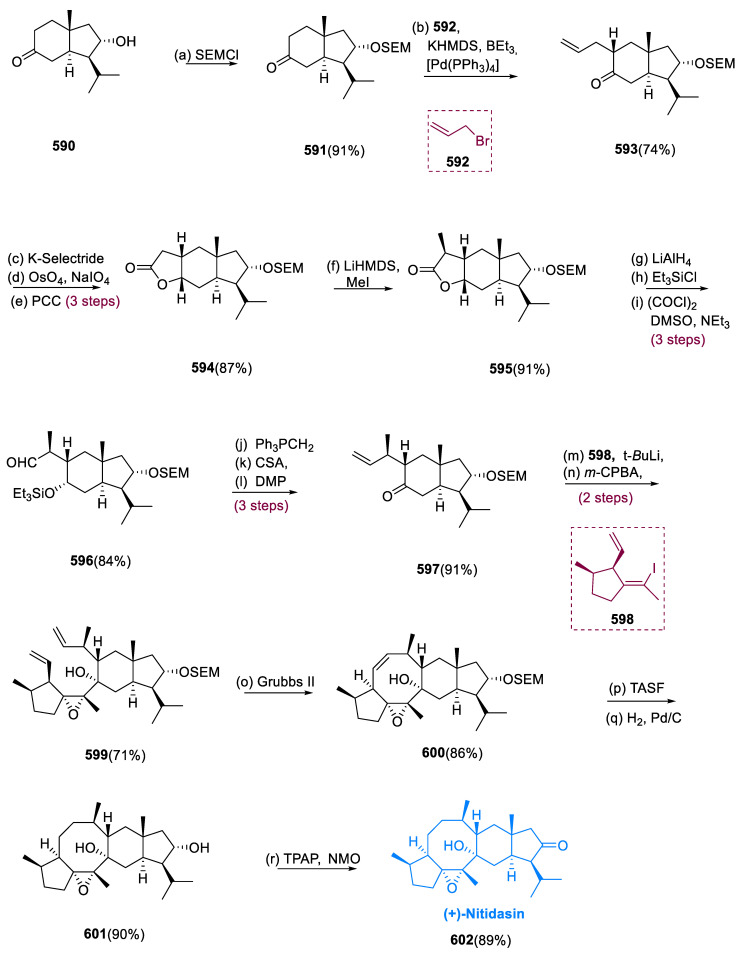
Total synthesis of (+)-nitidasin.

Finally, a -step procedure comprising Wittig olefination, oxidation, and chemoselective deprotection yielded trans-hydrindanone (**597**), which allows **5** out of the **10** stereo-centers. Treatment of trans-hydrindanone (**597**) to alkenyl lithium (**598**) instantaneously led to epoxidation (**599**). This two-step procedure gave metathesis substrate (**599**) as the only stereoisomer in a remarkable yield of 71%. The subsequent RCM proceeded smoothly in the Grubbs **II** to yield cyclo-octene (**600**). The double bond of alkene (**600**) underwent hydrogenation upon being treated with excess HMPA/TASF in the presence of MS at higher temperatures, which was quickly unmasked by the analogous secondary alcohol. Following hydrogenation, under the previous conditions, furnished “nitidasol” (**601**). This intermediate (**601**), a biosynthetic starting substrate of nitidasin, is potentially being recruited as a natural product. Then finally, the oxidation of nitidasol (**601**) by using NMO/TPAP gave nitidasin (**602**) (Scheme 35) [173].

### 7.3. Cerorubenic Acid-III

Cerorubenic acid-III (**625**), which belongs to the sesterterpenoid with a unique tetracyclo[8.4.1.0.0]pentadecane framework, was first extracted by Naya and coworkers in 1983 from *Ceroplastes Maskell Rubens*, insect secretions [174]. It plays a main role in the communication of insects [175]. On a number of cell lines, it also displayed remarkable but inert cytotoxic effects with an IC_50_ range of 0.057-3.4 μM in vitro. It also influences the transcriptional level of multiple pathways associated with cell survival, such as in apoptosis, cell cycle, and inflammation [176].

Cerorubenic acid-lll (**625**) retains an overwrought bridge head, anti-Bredt double bond at C-6, C-7, as it also originates in the well-known Taxol, a natural drug [177].

The organocatalytic Michael addition of substrate (**603**) to methyl vinylketone (**604**) was stimulated by employing 5 mol% of proline-derived initiator (**605**) together with ethyl 3, 4-dihydroxy benzoate (**606**) as a 20 mol% of cocatalyst. Then, intramolecular aldol condensation and dehydration were performed by employing i-PrOH and LiOH-synthesized molecule (**607**) in a 72% yield (93% de). The silyl enol ether treated with n-BuLi, followed by setting up the consequential enolate with the aldehyde (**609**) in the presence of zinc bromide, was reduced simultaneously via DIBAL-H to synthesize the stable diol (**610**) in a 50% yield from the molecule (**607**). Treatment of diol (**610**) underwent treatment with TsOH in acetone following TBAF in addition to giving intermediate (**611**) in an 85% yield. Intermediate (**611**) underwent oxidative rearrangement via VO(acac)_2_ and TBHP in CH_2_Cl_2_, following one-pot protection of hydroxyl function by the acetyl group, synthesizing the key starting molecule (**612**) in a 71% yield.

Starting molecule (**612**) underwent type-II-[5+2] cyclo-addition by using TMP in an airtight tube with heating, yielding molecule (**613**) as a single stereoisomer in a 72% yield. Compound (**613**) reduced to intermediate (**615**) gave a 98% yield. The hydroxyl group at C-5 in intermediate (**615**) was transformed to the equivalent bromide using a nucleophilic exchange approach to obtain compound (**616**) in a 67% yield. Compound (**616**) was further treated with Na naphthalenide at r.t. in THF and H_2_O to synthesize diol (**618**) in 78% via intermediate (**617**), which could be separated in an anhydrous state. Na-naphthalenide plays several roles in selectively removing the bond in C_4_–*O* (**616**), then eradicating the Bn moiety and reducing the sterically less-hindered double bond of the diene in intermediate (**617**) at *C*-4 and *C*-5.

The primary hydroxyl group in compound (**618**) was protected by TBS, following tosylation of the C_24_-OH functional moiety and elimination of the TBS in a one-pot step undergoing oxidation via DMP, yielding compound (**619**) in a 55% yield. The desired trans-annular cyclization of compound (**619**) using tBuOK in t-butanoic acid delivers cyclopropyl aldehyde (**621**) diastereoselectively in a 78% yield. The reaction progressed through a cationic conduit via intermediate (**620**), with the attacking of enolate at the C-24 cation steadied by the contiguous vinyl moiety. Huang’s variation of Wolff–Kishner was used to alter the formyl group of cyclopropyl aldehyde (**621**) to a methyl group, following simultaneous deprotection via acetic acid to make the diol (**622**). Chemoselective acetylation of the sterically less-hindered -OH group in diol (**622**), monitored by DMP using a one-pot method and following base-intermediated exclusion, furnished the enone (**623**) in an 80% yield.

**Scheme 36:**
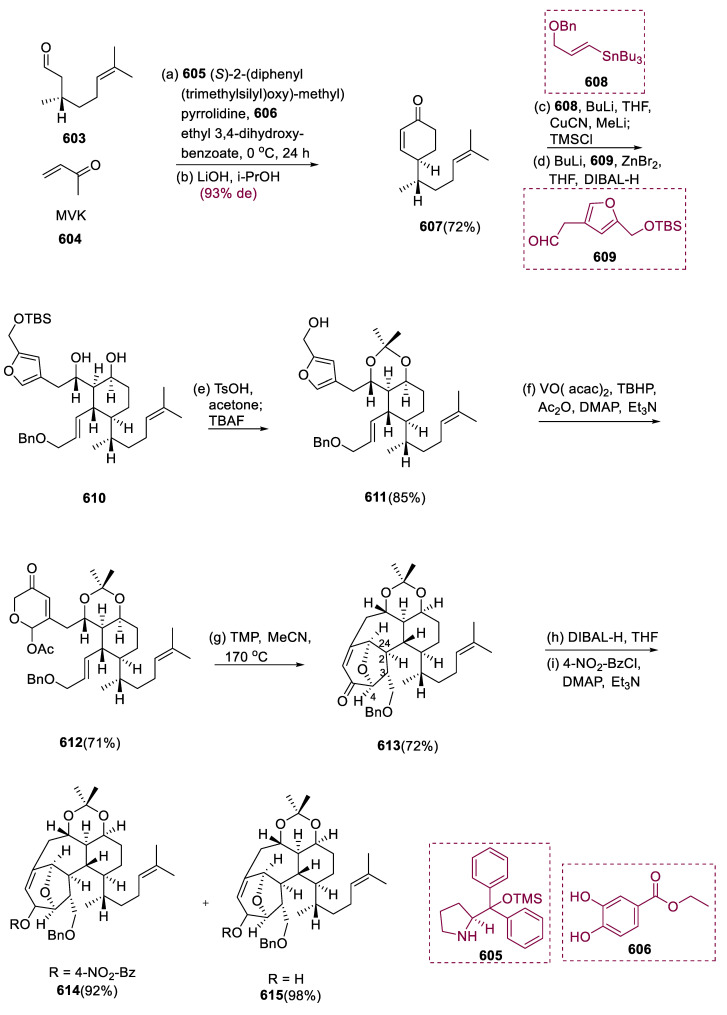

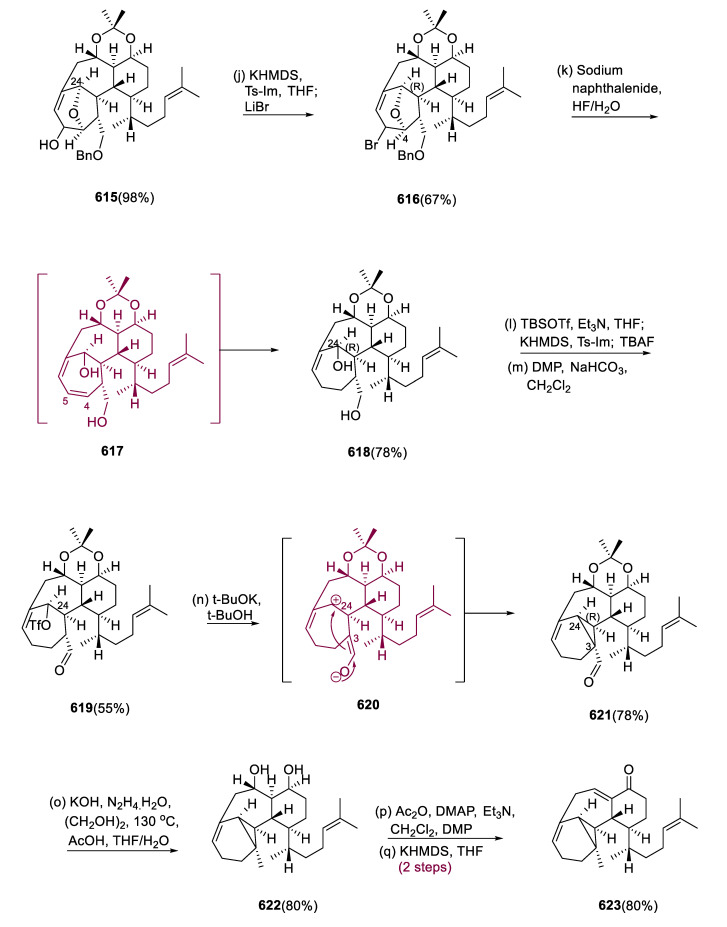

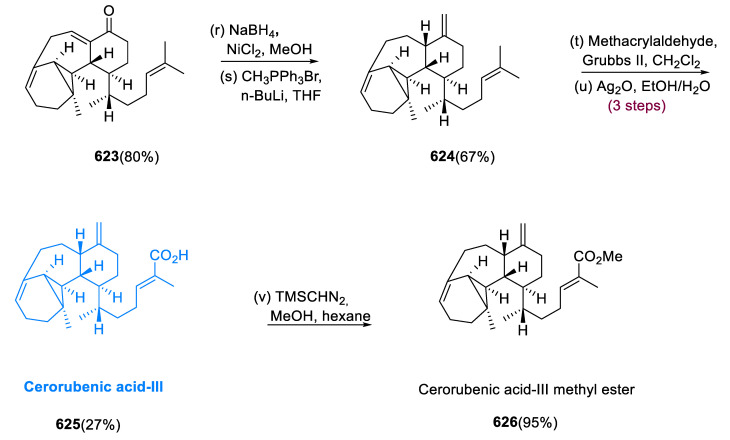
Total synthesis of cerorubenic acid-lll.

Enone (**623**) following 1,4-reduction via NiCl_2_/NaBH_4_ and carbonyl group involving Wittig olefination yielded the triene (**624**), that was in situ following the oxidation of silver oxide to give intermediate (**625**) in a 27% yield from triene (**624**). Cerorubenic acid-III having methyl ester (**626**) was prepared from intermediate (**625**) by TMS diazomethane in a 95% yield (Scheme 36) [178].

### 7.4. Ansellone A

Ansellone A (**637**) and its analogs (**638,639**) belong to the marine sester-terpenoids, isolated from the *Cadlina luteomarginata* dorid nudibranch and *Phorbas* species [179]. These sester-terpenoids keep the unique “ansellane” carbon framework and are primarily stated as a *c*-AMP stimulator. Then, biological screening of a succession of sester-terpenoids showed that ansellone A (**637**), along with its analogs (**638,639**), displays LRA evaluation. These marine-containing sester-terpenoids displaying LRA activities were reported to stimulate protein-kinase *C*-signaling [180].

Diol (**627**) then underwent oxidation of alcohol using IBX in dichloro-ethane, undergoing heating/hydrolysis of the acetate function giving ketone diol (**628**) (82%). Treatment of ketone diol (**628**) with 2, 6-lutidine, and t-Bu_2_Si(OTf)_2_ resulted in the synthesis of cyclic silyl ether (**629**) (92%). Using 2, 4-di-tertiary butyl pyridine and Tf_2_O successfully provided compound (**630**) in excellent productivity. Elimination of the silyl motif under mild conditions via TBAF with acetic acid and successive IBX-oxidation of primary alcohol yielded aldehyde (**632**). TMSOTf stimulated the Prins cyclization between aldehyde (**632**) and intermediate (**633**) to achieve the stereoselective synthesis of the 2, 6 cis-tetrahydro pyran group. The elimination of the TfO group of compound (**634**) via PPh_3_ and Pd(OAc)_2_ in the presence of HCOOH and Et_3_N delivered compound (**635**) in an 87% yield. The conversion of compound (**635**) to the final product (**637**) was succeeded via variation of Tong’s two-step procedure.

**Scheme 37:**
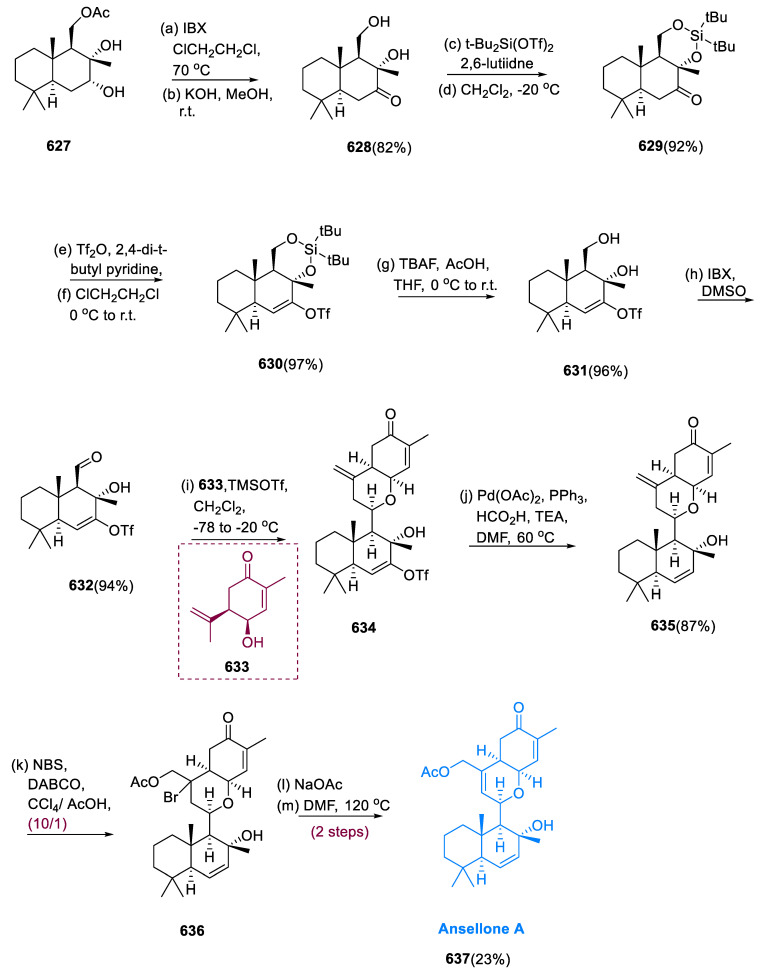

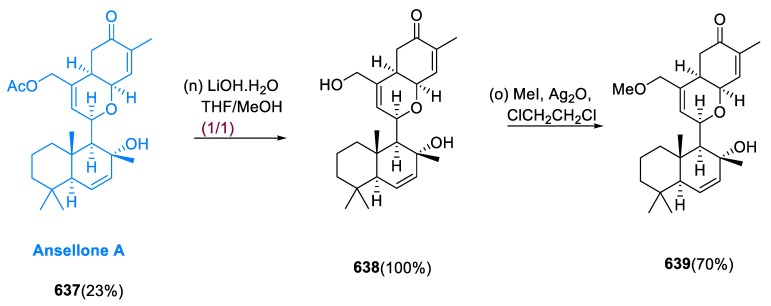
Total synthesis of ansellone A and its analogs.

Treatment of compound (**635**) with DABCO and NBS in CCl_4_/AcOH gave bromo-acetate (**636**). Further treatment with Na-acetate resulted in the removal of HBr to synthesize the product (**637**). The Ac-group of the final product (**637**) was hydrolyzed using lithium hydroxide to deliver the de-Ac analog (**638**). Then, methylation of (**638**) with methyl iodide and Ag_2_O synthesized other analogs (**639**) (70%) (Scheme 37) [181].

### 7.5. Leucosceptroids A

In 2010, Li and coworkers described the separation of leucosceptroids A and their analogs (**657**) from *Leucosceptrum canum*, which belongs to glandilar trichomes [182]. The preliminary biological potential has shown that these sesterterpenoids have potent antifeedant properties against the cotton bollworm and beet armyeorm, and antifungal effects against four strains of agricultural fungal pathogen, including *Rhizoctonia Solani* and *Colletotrichum Musae* [183,184,185].

The coupling of 3-bromo-prop-1-yne and the cuprous salt obtained from enynol (**640**) formed a diyne, which was reacted with a gold catalyst in the presence of Ag triflate to yield furan (**641**) in an 85% yield. After the conversion of furan (**641**) into a titanium-acetylide, Felkin anhydrous reduction of the aldehyde (**642**) was carried out to afford antiadduct (**643**) in a 78% yield with (7:1ds). Fe-catalyzed carbometalation of propargylic alcohol (**643**) with MeMgBr progressed efficiently to provide allyl alcohol (**644**) which was deprotected and the released alcohol reprotected with TBDPSCl to yield diol (**645**). PhSeCl-intermediated cyclization of diol (**645**) led to the diastereoselective synthesis of substituted tetra-hydrofuran (**647**). Without separation, substituted THF oxidized directly with hydrogen peroxide to deliver dihydro-furanone (**647**) in a 78% yield. Further treatment with an alkenyl aldehyde (**648**) in the presence of (Hex)_2_BCl/Et_3_N gave secondary alcohol (**649**).

Reduction of a secondary alcohol (**649**) with Me_4_NB(Oac)_3_H gave diol (**650**), which led to regioselective acetylation and subsequent oxidation of hydroxyl group to yield ketone (**651**) in 69%. The reduction in ketone (**651**) with LiBH_4_ provided the alcohol with the desired stereochemistry, and the resulting diol was oxidized selectively with IBX to form ketone (**652**) after TMSCl protection. The SmI_2_-interceded cyclization of ketone (**652**) gave the preferred triol (**653**) as the only product after desilylation in an 89% yield (overall).

**Scheme 38:**
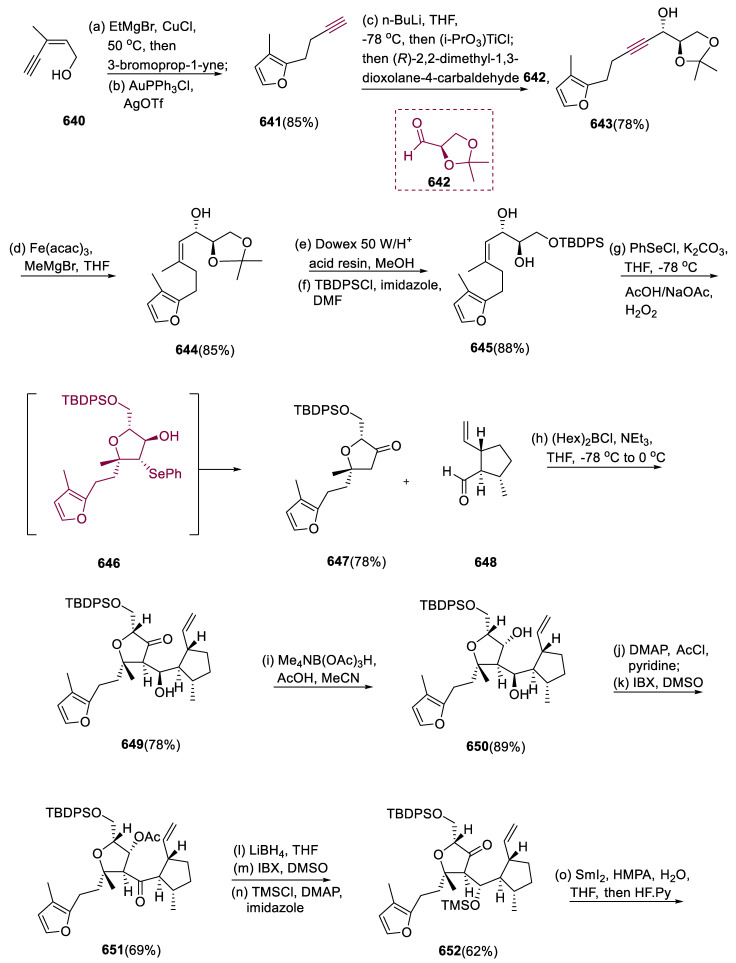

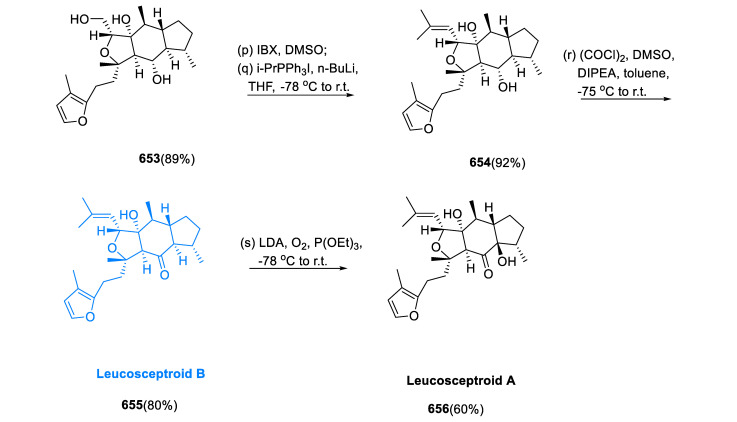
Total synthesis of leucosceptroid B and their analogs.

The primary alcohol in triol (**653**) undergoes selective oxidation, which following Wittig olefination gave diol (**654**), which was transformed into compound (**656**) through PCC oxidation. Regioselective α-hydroxylation in the presence of LDA, then P(OEt)_3_/O_2_) of compound (**655**) provided leucosceptroid A (**656**) in a 60% yield (Scheme 38) [186].

### 7.6. Aplysinoplide B

In 2008, Matsunaga et al. isolated novel cytotoxic sesterterpenoids, aplysinoplide B (**668**) from the marine sponge *Aplysinopsis digitata* (marine sponge), collected at Shinsone-Oshima [187]. Aplysinoplide B (**668**) showed cytotoxic properties against P-388 leukemia cells of a mouse having IC_50_ values (0.45, 11 µg/ML) [188,189,190].

Propargyl alcohol (**657**), freely available in two steps from commercially existing 3 butyn-1-ol, was transformed into *Z*-alkene (**659**) via homopropargyl alcohol (**658**). Then, *Z*-alkene (**659**) was successfully converted into *Z*-vinyl bromide (**660**) as a single product in excellent yield, employing a one-pot bromination process. 9-B-alkyl Suzuki coupling of vinyl bromide (**660**) yielded triene, which was synthesized from commercially available di hydro-β-ionone following the Wittig reaction. The Suzuki coupling (sp^2^-sp^3^) succeeded, giving the preferred product (**663**), which upon deprotection of a tertiary butyl dimethyl-silyl with TBAF yielded the allyl alcohol (**664**) in a 56% yield (two steps).

**Scheme 39:**
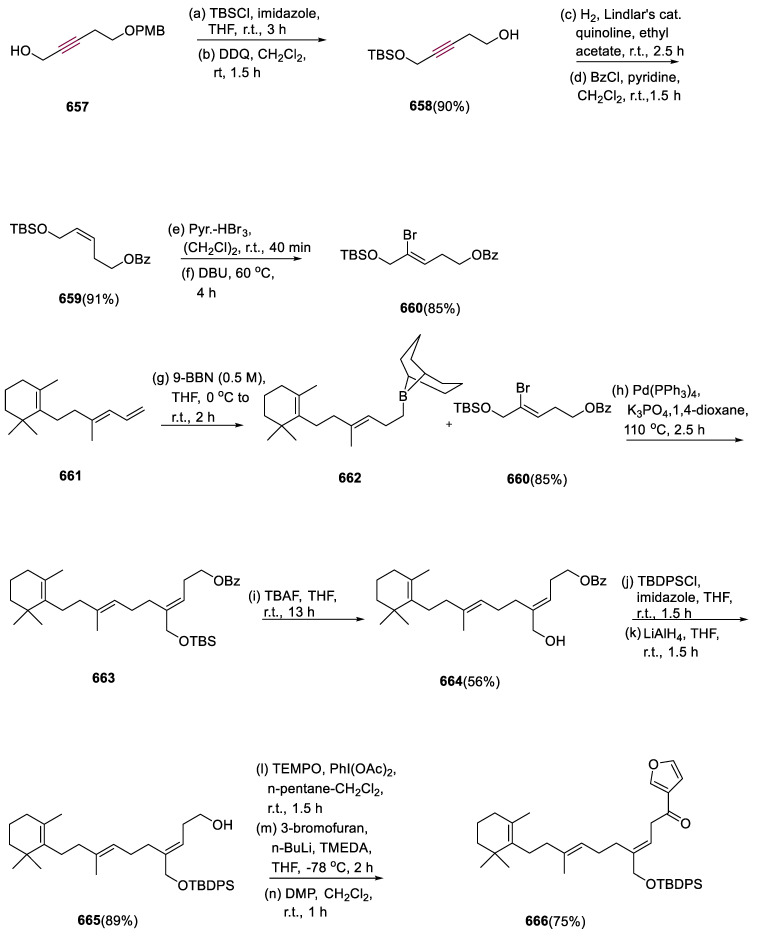

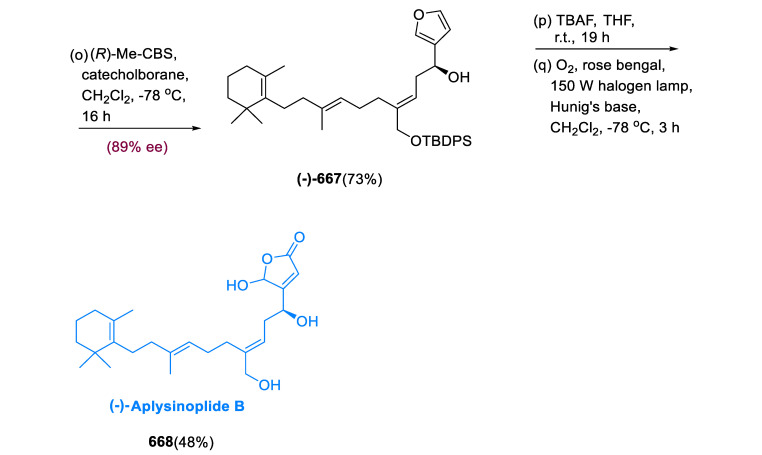
Total synthesis of (–)-aplysinoplide B.

Homoallyl alcohol (**665**) was achieved by removing the protecting group, following TEMPO-interceded oxidation, the addition of 3-lithio furan to the synthesized aldehyde, and DMP oxidation yielded the furanyl ketone (**666**). The Corey–Bakshi reduction in furanyl ketone (**666**) following (*S*)-2-methyl CBS oxa-zaborolidine and (*R*) 2-methyl CBS-oxa-zaborolidine synthesized the (*S*)-alcohol (–)-(**667)** in a 73% yield. Deprotection of both enantiomeric alcohols (–)-**667** with tetra-butyl ammonium fluoride and the subsequent region-selective oxidation of the 3-alkyl furan with [O] in the manifestation of Hünig’s base generated (–)-Aplysinoplide B (**668)** in a 48% yield (Scheme 39) [191].

## 8. Meroterpenoids

### 8.1. Manginoid A

Manginoid A (**691**) contains shikimic acid-conjugated meroterpenoids derived from *Guignardia mangiferae*, a plant pathogen [192]. Manginoid A (**691**) inhibits 11β-HSD1 (11β- hydroxyl steroid-dehydrogenase type 1), an enzyme that activates the intracellular transformation of cortisone to bioactive glucocorticoid cortisol, with an IC_50_ of 0.84mM. This variation could deliver a unique healing path in contrast to metabolic syndromes such as diabetes, osteoporosis, and obesity [193]. Manginoid A (**691**) has a unique oxabridged spiro cyclohexane-dione moiety with a highly substituted trans-hydrindane structure.

Following iso-propenyl addition to commercially available cyclopentenone to yield (**669**), treatment with TIPSOTf and KHMDS at 78 °C in THF resulted in a (2.5:1) mixture of regioisomeric silyl enol ethers, with (**670**/**671**) favored. Compound (**673**) was obtained in a 38% yield after further exposure to SnCl_4_ in methylene dimethyl-malonate (**672**). Using trimethyl orthoformate, ethylene glycol, and *p*-TsOH•H_2_O in hot toluene at 90 °C led to the ketone inside (**674**) being protected as a ketal, and epimers of the α-carbon were induced to generate compound (**674**) as a single stereoisomer in an 86% yield. Allylic chlorination with trichloroisocyanuric acid (TCCA) was followed by NaH-activated intramolecular cyclization. After that, an extensive reduction of both esters with LiAlH_4_ yielded (**675**). The addition of Dess–Martin periodinane resulted in a 65% total yield of aldehyde (**676**).

By adopting the Bestmann modification to the Seyferth–Gilbert homologation, deprotonating the newly generated terminal alkyne, and adding electrophile (**679**), compound (**680**) was synthesized from a mixture of diastereomers. The Zn powder in acetic acid reductively cleaved iodo-ether. The resulting product (**681**) was then hydrogenated with Pd/C putrefying through quinoline to give the cis-alkene (**682**) a whole yield of 88% from the compound (**680**). Crude dicarbonyl (**683**) was produced directly in good yield using Dess–Martin periodinane following a standard workup with saturated aqueous NaHCO_3_ and Na_2_S_2_O_3_ solutions. After treating dicarbonyl (**683**) with SmI_2_ in THF at r.t., the desired diol (**684**) emerged as a single diastereomer.

**Scheme 40:**
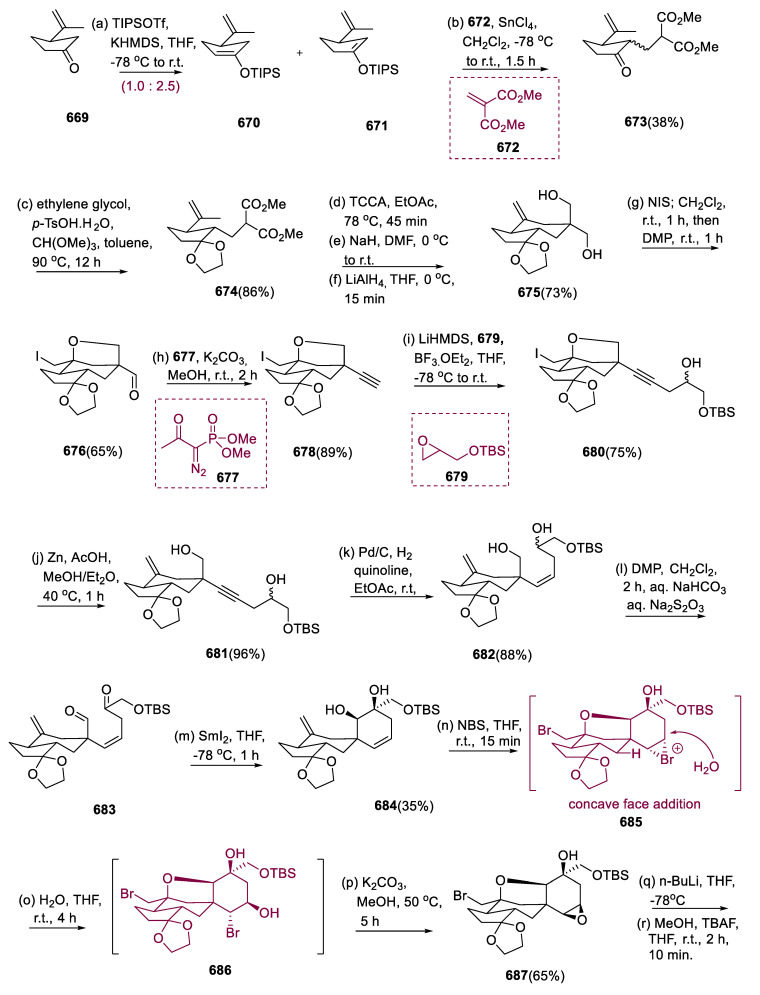

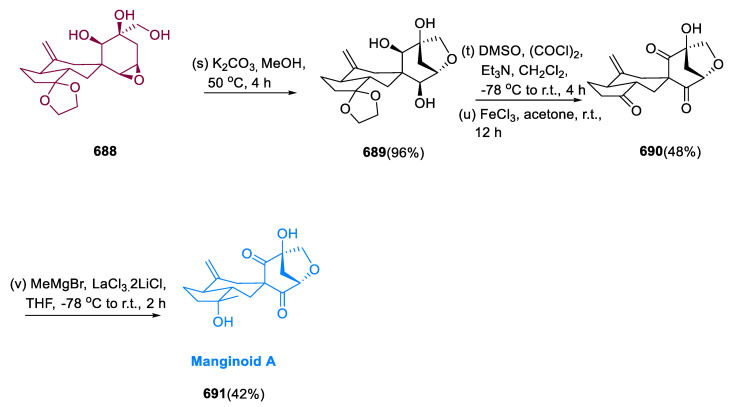
Total synthesis of manginoid A.

Following the production of a molecule (**685**) from diol (**684**) by using excess *N*-bromo succinimide in the presence of H_2_O facilitated the formation of bromohydrin (**686**), which was entirely stereo- and regioselective. Then, adding a base yielded the anticipated β-disposed-epoxide of a molecule (**687**) in a 65% yield. N-BuLi-promoted halo ether ring-opening following silyl deprotection yielded (**688**), whereas later, base-promoted etherification yielded (**689**) in a 96% yield. Double Swern oxidation was followed by DMP/PCC ketal breakdown and FeCl_3_ to give (**690**) in a 48% total yield. Finally, employing a mixture of 2.1 equivalents of LaCl_3_•2LiCl and 2.1 equivalents of MeMgBr in THF at −78 °C, a regio-selective addition of the Me group yielded manginoid A (**691**) as a single stereoisomer in a 42% yield (Scheme 40) [194].

### 8.2. Hyperjapone A

Hyperjapone A (**695**) belongs to the meroterpenoids extracted from *Hypericum japonicum*, used to treat gastrointestinal disorders, hepatitis, and tumors in conventional Chinese remedies [195,196]. Hyperjapone A (**695**) having an 11-6-6 tricyclic ring skeleton belongs to a natural product in racemic form. Further racemic analogs, hyperjaponols A–C (**695-698**), have been separated from *Hypericum japonicum* [197].

Total synthesis of hyperjapone-A (**695**) started with Friedel–Crafts acylation of compound (**692**) phloroglucinol with isobutyryl chloride to provide acyl phloroglucinol, which was dearomatized using trimethylation to yield compound (**693**) norflavesone (2 steps). Oxidation of norflavesone (**693**) with Ag_2_O and TEMPO gave hyperjapone-A (**695**) in a 32% yield, through a hetero Diels–Alder among the α, β-unsaturated ketone, generated instantly, and humulene (**694**). Treatment of hyperjapone-A (±)-**695** with *m*-CPBA gave epoxide (**696**) as a major diastereomer in a 76% yield over diastereoselective oxidation of the Δ 8, 9 alkenes.

**Scheme 41:**
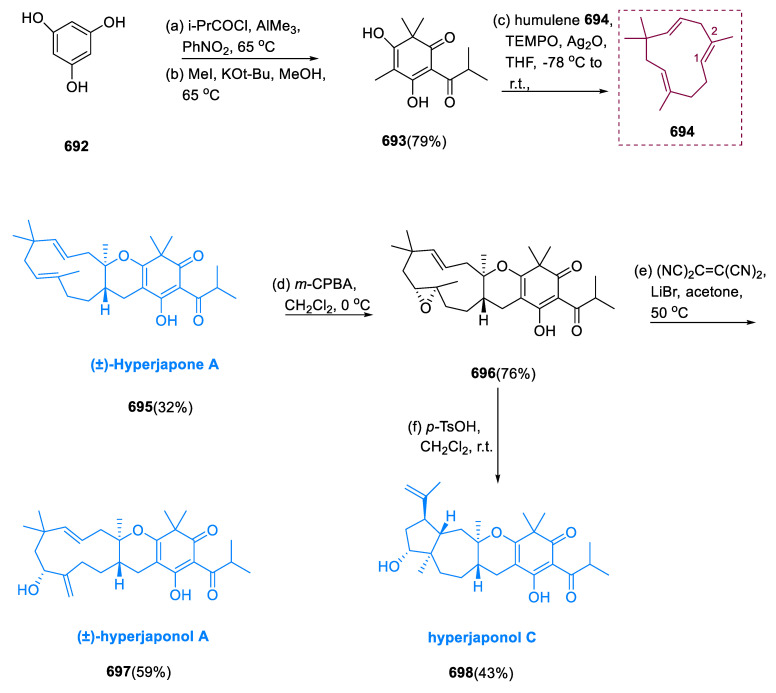
Total synthesis of (±)-hyperjapone A and their racemic analogs.

Acid-mediated rearrangement of epoxide (**696**) following *p*-TsOH in CH_2_Cl_2_ afforded hyperjaponol-C (**698**) in a 43% yield. Transformation of epoxide (**696)** into (±)-hyperjaponol-A (**697**) was obtained in a 59% yield by treating LiBr and (NC)_2_C=C(CN)_2_ in acetone (Scheme 41) [198].

### 8.3. Myrotheciumone A

Myrotheciumone A (**707**) was extracted by Lin et al. from Roridum Myrothecium, an endophytic fungus, *Ajuga decumbens* (Medicinal herb) in 2014 [199]. Myrotheciumone A **(707**) exhibited anticancer activity of IC_50_ values (5.36−7.56 μM) and prompted the PARP [poly (*ADP*-ribose) polymerase] breakdown in a time- and dose-dependent way [200]. It also displayed cell-specific cytotoxicity by prompting apoptosis to the targeted tumor cell relatively more than healthy cells and promoting the escape of cytochrome-C from mitochondria [201].

**Scheme 42:**
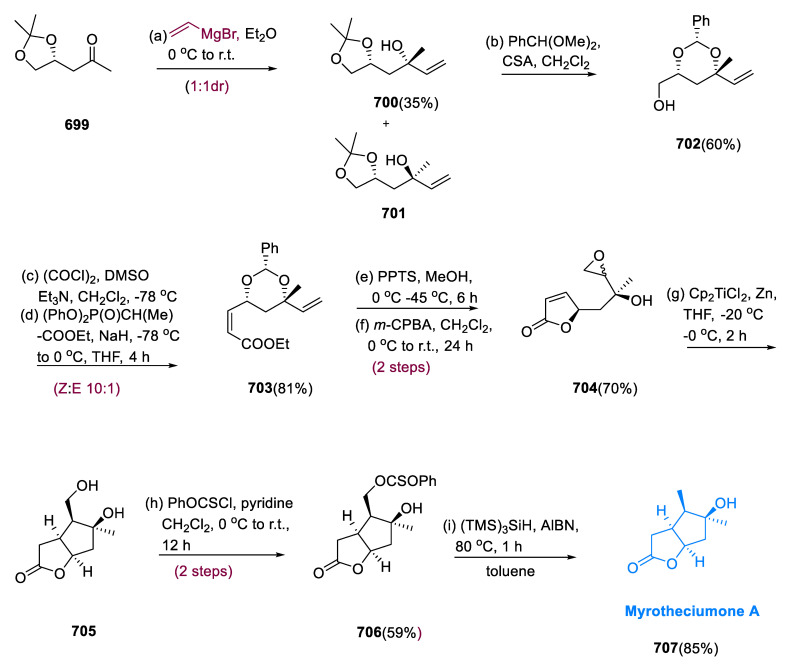
Total synthesis of myrotheciumone A.

The 3° allylic alcohols (**700**) and (**701**) were synthesized by reacting methyl ketone (**699**) with vinyl magnesium bromide in exactly (1:1) dr at 0 °C with a total of 35% yield. The t-alcohol (**700**) was then openly exposed to 1, 3-protection via benzaldehyde dimethyl acetal through a catalytic amount of CSA (camphor-sulfonic acid) to provide the primary alcohol (**702**) in a 60% yield. Oxidation of alcohol (**702**) and then the Horner–Wadsworth olefination with ethyl 2-(diphenoxy phosphoryl) acetate gave compound (**703**) in (10:1) in an 81% yield. The major cis-isomer led to deprotection and lactonization by PPTS in MeOH at 45 °C to give the analogous *γ*-butenolide upon epoxidation with *m*-CPBA, providing the chosen epoxide (**704**) in an 70% yield (two steps). Epoxide (**704**) was exposed to titanocene-(III)-chloride to obtain the desired diol (**705**) as a single diastereoisomer. Diol **(705**) was then reacted with *O*-phenyl chlorothiono formate and C_5_H_5_N at 0 °C to r.t. to synthesize xanthate (**706**) in a 59% yield (two steps) from the epoxide (**704**), undergoing deoxygenation with AIBN as radical accelerator and (TMS)_3_SiH as H_2_ origin for 1 h at 80 °C to deliver the product myrotheciumone A (**707**), as colorless crystals in an 85% yield (Scheme 42) [202].

### 8.4. Merochlorin A

Merochlorin A (**728**) contains a tetracyclic ring skeleton that comprises a highly congested bicyclo[3.2.1]octane motif including four contiguous stereocenters, a resorcinol fragment, and a bridgehead chlorine. The groups of Fenical and Moore stated the separation of a unique class of meroterpenoids from the *Action-mycetes* strain (*CNH* 189) from sediments near the beach of Oceanside, California in 2012 [203]. Merochlorin A (**728**) was discovered to show in vitro antibiotic evaluation against *dificile*, having MIC values of 0.15–0.3 μM, and several multi-drug resistant *S. aureus* with MIC values ranging from 2–4μM, which are two protruding pathogens in control for hospital acquired disinfections [204].

The acyclic starting material for the main cascade cyclization (**712**) was synthesized by reformed Wittig–Schlosser among compound (**708**) triphenyl-phosphonium bromide and aldehyde (**709**). The transitional β-lithiooxy phosphonium ylide treated with di-iodoethane provided intermediate (**710**) (72% yield) having 10:1, which yielded a highly stereoselective approach to the substituted bis-alkyl-*E* alkenyl-iodide. The vinyl iodide was transformed to 1, 3 enyne aldehyde (**712**) in a 56% total yield from intermediate (**710**). Sonogashira coupling of intermediate (**710**) with enantio-enriched propargylic-acetate (**711**) succeeded by hydroboration with 9-BBN provided the resultant alcohol. Protection of benzoyl, desilylation, and Swern oxidation afforded cyclization starting material (**712**) in high quantity.

Treatment of cyclized starting material (**712**) with Echavarren’s catalyst (**713**) in tetra-hydrofuran containing H_2_O as 0.2 vol% at −10 °C easily yielded enone (**714**) in a 73% yield along with 11% of acetate (**715**). After removal of the secondary alcohol via triflation (Tf_2_O, 2, 6-lutidine) and base (65%), regioselective Tsuji–Wacker oxidation gave ketone (**716**) as a major product (85%). Eventually, a two-step process involving La (III)-interceded Grignard reduction of iso-propenyl magnesium bromide via Et_3_SiH, BF_3_·OEt_2_ providing the requisite enone (**717**) in a 56% yield (two steps). Homo-isoprene subunit (**718**) was reduced to enone (**717**) in the form of a Lipshutz higher-demand cyanocuprate to set the (II)-quaternary stereogenic center. The hollow nature of the pentalenone involves an exclusive exo-attack, delivering the resultant ketone (**719**) in 87% productivity as a single stereoisomer. The oxidation of molecule (**719**) to the COOH (DMF, PDC) succeeded by adding sodium acetate in acetic anhydride, which afforded enol lactone (**720**) in a 68% yield (two steps). The treatment of enol lactone (**720**) with DIBAL-H at −78 °C followed reductive rearrangement to synthesize tricyclic ketol (**723**) as a major diastereoisomer in an 85% yield.

**Scheme 43:**
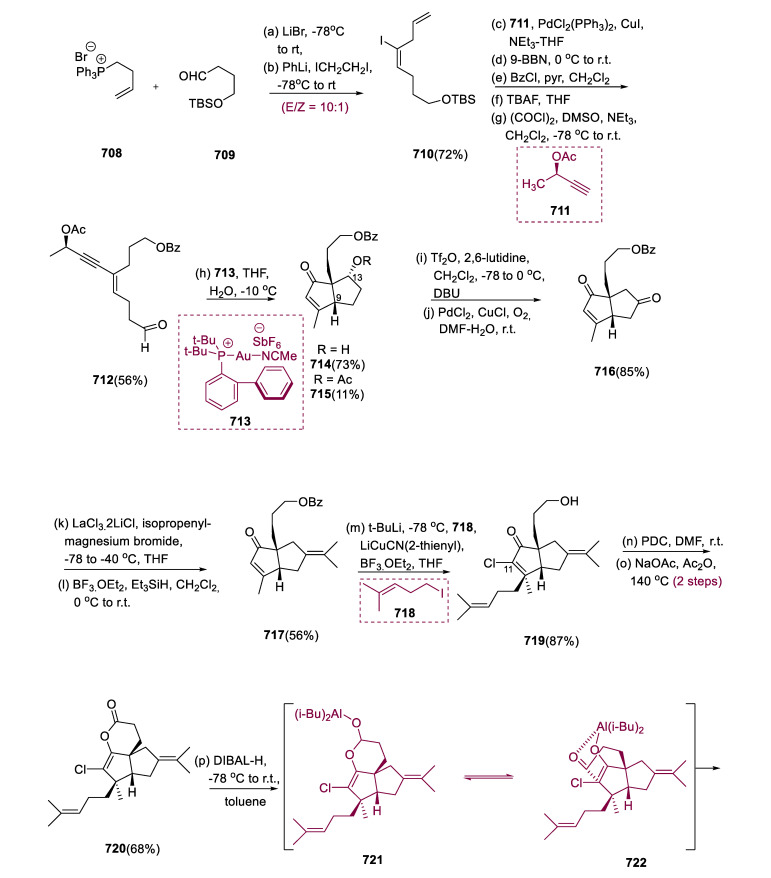

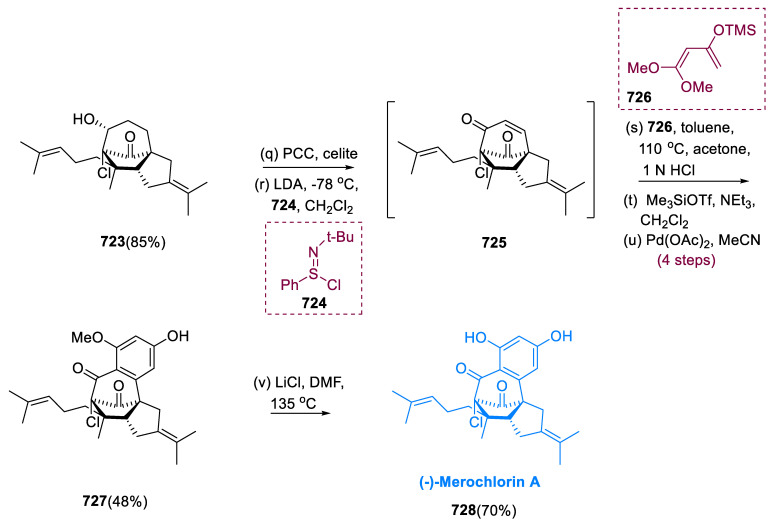
Total synthesis of (–)-merochlorin A.

PCC initiation for oxidation of ketol (**723**) yielded the resultant diketone. The diketone was found to be reactive on silica and prone to undergoing a retro Claisen sequence. Nevertheless, using PCC as an oxidant facilitated its clean and prompt separation by filtration with celite and gave the diketone purity (94% yield). Deprotonation with LDA at −78 °C following via the addition of molecule (**724**) yielded enone (**725**), which was further reacted with Brassard’s diene (**726**) in toluene at 110 °C. After acidic workup and filtration through silica gel, the crude Diels–Alder mixture underwent oxidation via Saegusa Ito to provide monomethylated merochlorin A (**727**) in 48% productivity (four steps). In conclusion, heating monomethylated merocholin A (**727**) at 135 °C for 4 h with lithium chloride in DMF gave (–)-merochlorin-A (**728**) in a 70% yield (Scheme 43) [205].

### 8.5. Cytospora Rhizophorae-A

The EtOAc-extract of Cytospora rhizophorae-A (**761**), a fungus isolated from *M*. *officinalis,* leads to the separation of four new meroterpenoids, specifically cytosporins A-D, a unique benzo [b][1,5]dioxocane skeleton representing a benzophenone and hemiterpene moieties. *Morinda officinalis* (family Rubiaceae), has been widely used in conventional herbal remedies and dietary supplements for nearly 2000 years [206,207]. Its pedigrees are broadly used for the cure of irregular menstruation, sexual impotence, spermatorrhea, female infertility [208], antiosteoporosis [209] and antifatigue [210], antioxidant [211], hypoglycemic, and antidepressant effects [212].

**Scheme 44:**
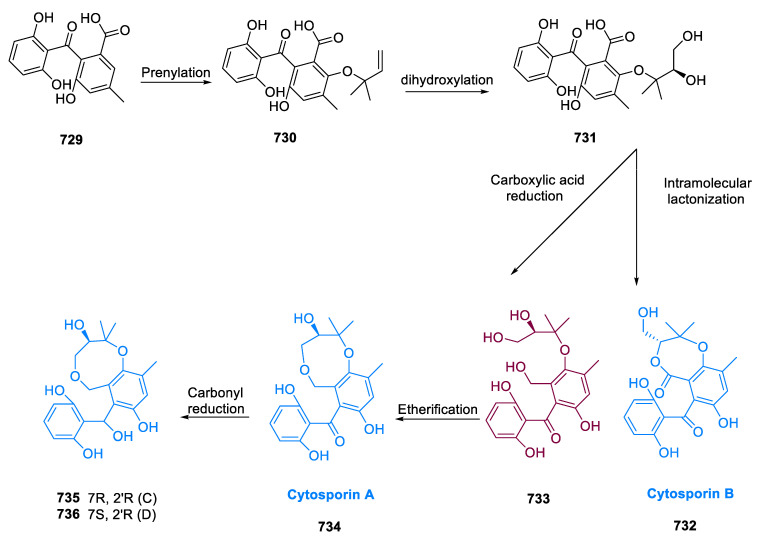
Total synthesis of cytosporins A-D.

Cytosporins A-D (**732–736**) belong to the class of benzophenone hemiterpene conjugated heterodimers, featuring an unprecedented strained seven-to-eight-membered ring structure. Cytosporins A-D (**732–736**) have to be made via a unified starting material (**731**), obtained from monodictyphenone, an unusual natural compound (**729**) by the insertion of the densely functionalized hemiterpene core through chemoselective prenylation and stereoselectively dihydroxylation. The intramolecular lactonization of the starting molecule (**731)** would directly convert it to be cytosporin-B (**732**), while cytosporin-A (**734**) might be accessible from the starting molecule (**731)** through the reduction of carboxylic acid and etherification. Furthermore, cytosporins C-D (**735**, **736**) could be easily inferred from cytosporin-A (**734**) by a simple reduction of carbonyl (Scheme 44) [213].

### 8.6. Taondiol

Taondiol (**751**) was extracted from marine algae (*Atomaria taonia*) by Gonzalez et al. in 1971 [214]. Fenical and his group in 1980 reported metabolites of algae from *Zonale Stypopodium*, and also isolated taondiol, an optical antipode that was reported by the Gonzalez. The enantiomers (+)-taondiol and (–)-taondiol are naturally occurring, contradictory to their origin in marine algae [215]. Taondiol is a basic congener of the Stypopodium class, consisting of a benzopyran moiety. These marine alkaloids exhibit cytotoxic and ichthyotoxic activities. They also exhibit diverse lethargic activities and narcosis at 10 μg/mL intensities [216].

The synthetic order was initiated by the selective protection of ketal of the Wieland Miescher ketone derivative (**737**) by ethylene glycol. A Robinson-type annulation was then stimulated on the bicyclic part to achieve the tricyclic diterpene scaffold. Using 1 chloro 3-pentanone/KOt-Bu succeeded by an intramolecular aldol condensation provided enone (**738**) in an 83% yield through thermodynamically controlled alkylation (two steps). The tricyclic moiety, which underwent reductive methylation on enone derivative (**738**) was conceded via Li/MeI/NH_3_ to afford ketone (**739**) in an 80% yield. Ketone (**739**) underwent LAH reduction, progressed by benzylation of the alcohol, giving the tricyclic benzyl compound (**740**) which further led to acid-accelerated deketalization to synthesize molecule (**741**) in a 90% yield. α-methylation of ketone (**742**) was then conceded out with LDA and MeI, following epimerization at the newly formed stereocenter by reacting with NaOMe in MeOH to achieve the single diastereomer of molecule (**743**) in an 80% yield. The second-last step was Nozaki–Yamamoto homologation on ketone (**743**) to achieve the unsaturated aldehyde (**744**), which upon Luche reduction delivered the desired tricyclic alcohol (**745**) in a 57% yield.

Hydroxyquinol (**746**) involving the Friedel−Crafts mechanism with diterpenoid (**745**) employing BF_3_·OEt_2_ promptly synthesized a pentacyclic meroterpenoid scaffold (**747**) by sequential C−C/C−O formation in a 70% yield. A prominent observation was the selective cyclization at the olefin through the requisite hydroxyl group. That could be endorsed to the intramolecular hydrogen interaction in the middle of the other hydroxy and its contiguous methoxy moiety that prevents its contribution to the cyclization reaction. Deoxygenation of pentacyclic scaffold (**747**) was then succeeded by transforming the hydroxyl scaffold into triflate via triflic anhydride to achieve the triflate (**748**) in an 85% yield, following Pd(PPh_3_)_4_-mediated reduction of triflate to synthesize (**749**) in a 60% yield.

**Scheme 45:**
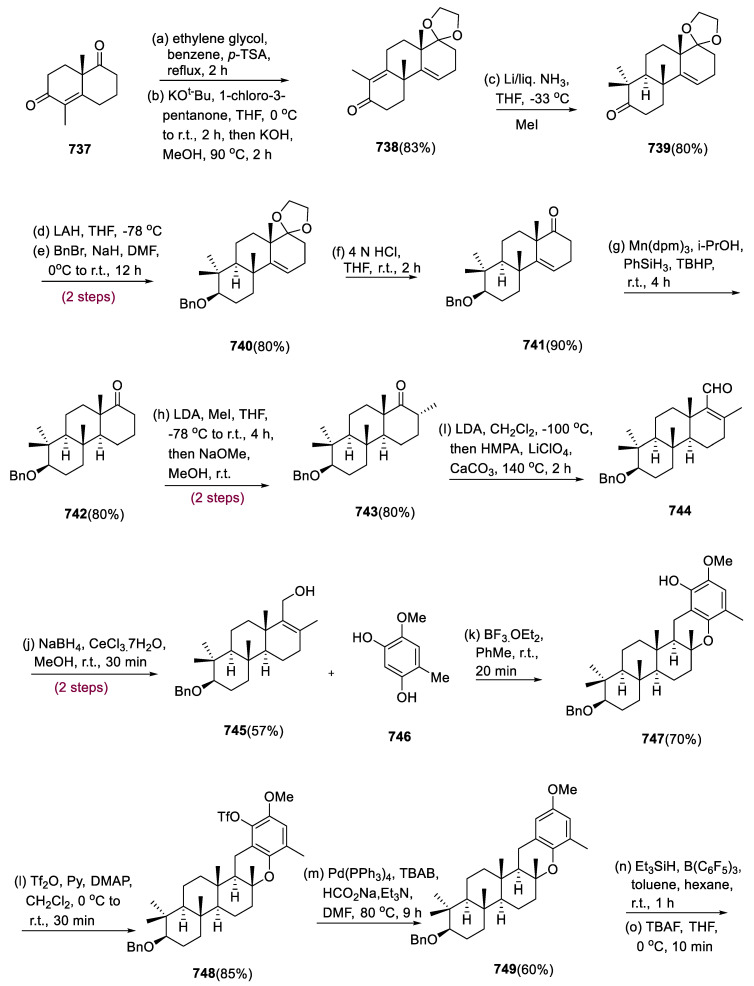

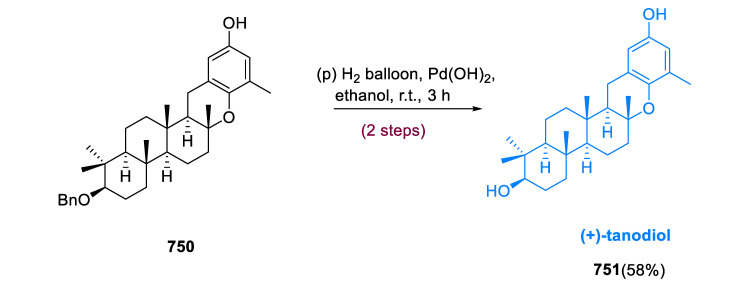
Total synthesis of (+)-tanodiol.

The upcoming step was the demethylation of the methoxy group in intermediate (**749**). Unfortunately, the common procedures of demethylation through EtSH/NaH and BCl_3_, BBr_3_ failed to deliver the preferred demethylated yield. We obtain demethylation upon applying Yamamoto’s procedure through B(C_6_F_5_)_3_/tri-ethylsilane to achieve the silyl ether and then deprotection of the silyl ether with TBAF to achieve the required demethylated yield. Finally, hydrogenolysis of a molecule (**750**) using H_2_/Pd(OH)_2_ synthesized (+)-taondiol (**751**) in a 58% yield (Scheme 45) [217].

### 8.7. Dysideanon B

Dysideanon B (**762**) was extracted from the Sponge *Dysidea avara* by Lin and coworkers in the South China sea in 2014. Dysideanon retains an unprecedented 6/6/6/6-joined tetracyclic scaffold and exhibits cytotoxicity counter to human melanoma cells and HepG-2 and HeLa cells, having IC_50_ values of 9.4 and 7.1 mm, respectively [218,219].

Highly unstable Wieland–Miescher conjugate ketone derivative (**752**), which may well be competently synthesized from commercially accessible material in either enantiomeric or racemic form, was chemoselectively masked as a glycol-acetal to afford enone (**753**) in a 94% yield (two steps). The combination of compound (**753**) bicyclic enone with the sterically hindered benzyl bromide (**754**) gives the desired C-9 alkylated ketone (**755**), which was achieved as a single diastereoisomer having an isolated yield (72%) in the presence of t-BuOK in THF at 40 °C. The methylenation was conducted by treating Nysted mixture with TiCl_4_ behaving as Lewis acid, giving terminal alkene (**756**) in a 31% yield and recovering 54% of ketone (**755**). The addition of intermediate (**756**) in 3M HCl gives ketoalkene (**757**), which further undergoes hydrogenation in the presence of (PPh_3_)_3_RhCl, giving the desired alkene bromide (**758**) in an 84% yield.

**Scheme 46:**
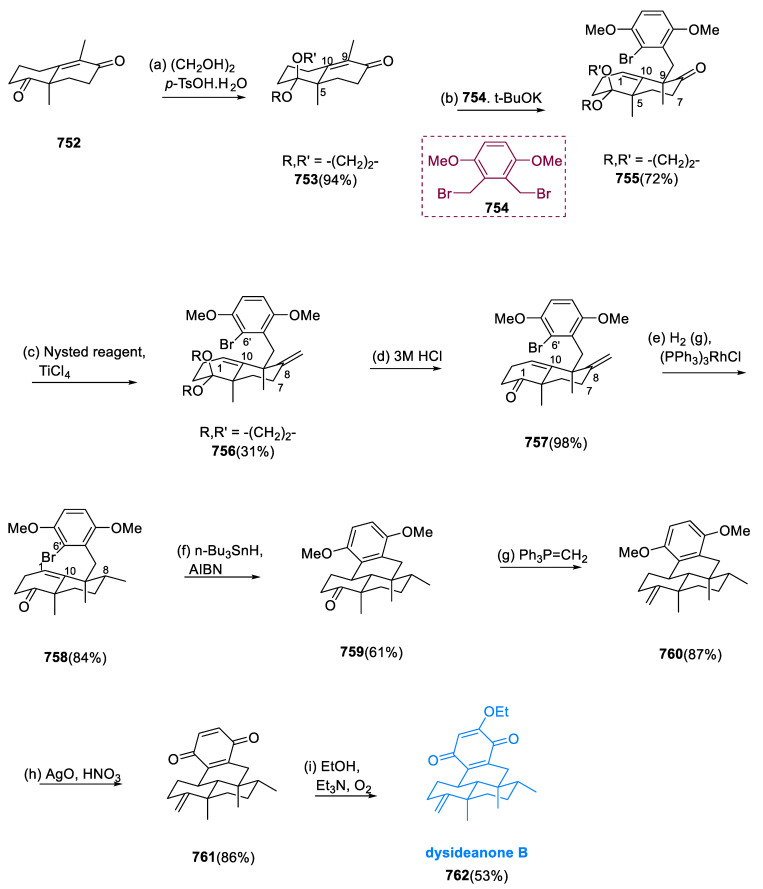
Total synthesis of dysideanone B.

Alkene bromide (**758**), undergoing radical reaction in the presence of AIBN and Bu_3_SnH, gave the estimated 6/6/6/6-fused tetra cycles (**759**), achieved in a 61% yield. Ketone underwent methylenation (**759**), which progressed efficiently with Wittig reagent to provide terminal alkene (**760**) in an 87% yield, whose methyl-protected hydroquinone moiety was oxidized via AgO/HNO_3_, affording quinone (**761**) in 86% yield. Et_3_N in EtOH under O_2_ proved active for the insertion of ethoxy functionality, achieving dysideanone-B (**762**) in a 53% yield (Scheme 46**)** [220].

## 9. Conclusions

Terpenoids, also known as isoprenoids, are a diverse group of compounds that are necessary to all living things. Future work focused at improving both screening methodologies for plant terpenoid lead compounds and novel approaches to understand complex biochemical processes of preexisting prospects will surely increase the rate of development and accessibility of plant terpenoids for medical use. The biggest and most diversified collection of naturally occurring substances is comprised of terpenes, sometimes referred to as terpenoids. They are divided into the categories of mono, di, tri, tetra, and sesquiterpenes according to the number of isoprene units they contain. They are mostly present in plants and constitute the majority of essential oils made from plants. The total synthesis, origin, and biological potential of several hemiterpenes, monoterpenes, sesquiterpenoids, diterpenoids, triterpenoids, sesterterpenoids, and meroterpenoids are covered in this review.

## Data Availability

Data sharing not applicable.

## References

[1] Gershenzon J., Dudareva N. (2007). The function of terpene natural products in the natural world. Nat. Chem. Biol..

[2] Nagel R., Schmidt A., Peters R.J. (2019). Isoprenyl diphosphate synthases: The chain length determining step in terpene biosynthesis. Planta.

[3] Guimarães A.G., Serafini M.R., Quintans-Júnior L.J. (2014). Terpenes and derivatives as a new perspective for pain treatment: A patent review. Expert Opin. Ther. Pat..

[4] Unsicker S.B., Kunert G., Gershenzon J. (2009). Protective perfumes: The role of vegetative volatiles in plant defense against herbivores. Curr. Opin. Plant Biol..

[5] Heil M. (2008). Indirect defence via tritrophic interactions. New Phytol..

[6] Martin D., Rojo A.I., Salinas M., Diaz R., Gallardo G., Alam J., De Galarreta C.M.R., Cuadrado A. (2004). Regulation of heme oxygenase-1 expression through the phosphatidylinositol 3-kinase/Akt pathway and the Nrf2 transcription factor in response to the antioxidant phytochemical carnosol. J. Biol. Chem..

[7] Pichersky E., Sharkey T.D., Gershenzon J. (2006). Plant volatiles: A lack of function or a lack of knowledge?. Trends Plant Sci..

[8] Lange B.M., Ahkami A. (2013). Metabolic engineering of plant monoterpenes, sesquiterpenes and diterpenes—Current status and future opportunities. Plant Biotechnol. J..

[9] Degu A., Engidawork E., Shibeshi W. (2016). Evaluation of the anti-diarrheal activity of the leaf extract of *Croton macrostachyus* Hocsht. ex Del.(Euphorbiaceae) in mice model. BMC Complement. Altern. Med..

[10] Dubey V.S., Bhalla R., Luthra R. (2003). An overview of the non-mevalonate pathway for terpenoid biosynthesis in plants. J. Biosci..

[11] Kirby J., Keasling J.D. (2009). Biosynthesis of plant isoprenoids: Perspectives for microbial engineering. Annu. Rev. Plant Biol..

[12] McNaught A.D., Wilkinson A. (1997). Compendium of Chemical Terminology.

[13] Zhu W., Liu X., Wang Y., Tong Y., Hu Y. (2018). Discovery of a novel series of α-terpineol derivatives as promising anti-asthmatic agents: Their design, synthesis, and biological evaluation. Eur. J. Med. Chem..

[14] Niinemets Ü., Monson R.K. (2013). Biology, Controls and Models of Tree Volatile Organic Compound Emissions.

[15] Fineschi S., Loreto F., Staudt M., Peñuelas J. (2013). Diversification of volatile isoprenoid emissions from trees: Evolutionary and ecological perspectives. Biology, Controls and Models of Tree Volatile Organic Compound Emissions.

[16] Lange B.M. (2015). The evolution of plant secretory structures and emergence of terpenoid chemical diversity. Annu. Rev. Plant Biol..

[17] Staudt M., Lhoutellier L. (2007). Volatile organic compound emission from holm oak infested by gypsy moth larvae: Evidence for distinct responses in damaged and undamaged leaves. Tree Physiol..

[18] Martin V.J., Pitera D.J., Withers S.T., Newman J.D., Keasling J.D. (2003). Engineering a mevalonate pathway in *Escherichia coli* for production of terpenoids. Nat. Biotechnol..

[19] Cho K.S., Lim Y.-R., Lee K., Lee J., Lee J.H., Lee I.-S. (2017). Terpenes from forests and human health. Toxicol. Res..

[20] Thoppil R.J., Bishayee A. (2011). Terpenoids as potential chemopreventive and therapeutic agents in liver cancer. World J. Hepatol..

[21] Huang M., Lu J.-J., Huang M.-Q., Bao J.-L., Chen X.-P., Wang Y.-T. (2012). Terpenoids: Natural products for cancer therapy. Expert Opin. Investig. Drugs.

[22] Croteau R., Johnson M.A. (1985). Biosynthesis of terpenoid wood extractives. Biosynthesis and Biodegradation of Wood Components.

[23] Brielmann H., Setzer W., Kaufman P., Kirakosyan A., Cseke L. (2006). Phytochemicals: The chemical components of plants. Natural Products from Plants.

[24] Zhao L.-M., Zhang S.-Q., Jin H.-S., Wan L.-J., Dou F. (2012). Zinc-mediated highly α-regioselective prenylation of imines with prenyl bromide. Org. Lett..

[25] Park S.H., Park K.H., Oh M.H., Kim H.H., Choe K.I., Kim S.R., Park K.J., Lee M.W. (2013). Anti-oxidative and anti-inflammatory activities of caffeoyl hemiterpene glycosides from Spiraea prunifolia. Phytochemistry.

[26] Kim M., Choi S., Park K., Kwon J., Oh M., Kim H., Jang J., Choe K., Park S., Lee M. (2011). Anti-oxidative and anti-inflammatory activities of caffeoyl derivatives from the barks of Ilex rotunda. Planta Med..

[27] Ohta N., Mori N., Kuwahara Y., Nishida R. (2006). A hemiterpene glucoside as a probing deterrent of the bean aphid, *Megoura crassicauda*, from a non-host vetch, *Vicia hirsuta*. Phytochemistry.

[28] Yang C., Wang Z., Qiu Y., Zha H., Yang X. (2020). New hemiterpene and furolactone-type lignan glycosides from Securidaca inappendiculata Hassk. Phytochem. Lett..

[29] Wang Z., Zha H., Yang X., Hu L., Zheng W., Xu L. (2017). Two new hemiterpene glycosides and one new phenolic glycoside from the roots of Securidaca inappendiculata Hassk. Phytochem. Lett..

[30] Kim M.H., Park K.H., Oh M.H., Kim H.H., Choe K.I., Park S.H., Lee M.W. (2012). Two new hemiterpene glycosides from the leaves of Ilex rotunda. thunb. Arch. Pharmacal Res..

[31] Jiang Z.-H., Wang J.-R., Li M., Liu Z.-Q., Chau K.-Y., Zhao C., Liu L. (2005). Hemiterpene Glucosides with Anti-Platelet Aggregation Activities from Ilex p ubescens. J. Nat. Prod..

[32] Liu L., Jiang Z., Li M., Wang J., Liu Z. (2008). Hemiterpene glycosides with anti-platelet aggregation activities from *Ilex pubescens*. U.S. Patent.

[33] Choudhary M.I., Naheed N., Abbaskhan A., Ali S. (2009). Hemiterpene glucosides and other constituents from *Spiraea canescens*. Phytochemistry.

[34] Zuo J., Ji C.L., Olatunji O.J., Yang Z., Xu H.F., Han J., Dong J. (2020). Bioactive fractions from *Securidaca inappendiculata* alleviated collagen-induced arthritis in rats by regulating metabolism-related signaling. Kaohsiung J. Med. Sci..

[35] Putri D.A., Santoso M. (2021). Synthesis of hemiterpene 1-bromo-3-methyl-2-butene derivatives. Proceedings of the AIP Conference Proceedings.

[36] Spisni E., Petrocelli G., Imbesi V., Spigarelli R., Azzinnari D., Donati Sarti M., Campieri M., Valerii M.C. (2020). Antioxidant, anti-inflammatory, and microbial-modulating activities of essential oils: Implications in colonic pathophysiology. Int. J. Mol. Sci..

[37] Kamatou G.P., Vermaak I., Viljoen A.M., Lawrence B.M. (2013). Menthol: A simple monoterpene with remarkable biological properties. Phytochemistry.

[38] Shah A.K., Maitlo G., Shah A.A., Channa I.A., Kandhro G.A., Maitlo H.A., Bhatti U.H., Shah A., Memon A.Q., Jatoi A.S. (2019). One pot menthol synthesis via hydrogenations of citral and citronellal over montmorillonite-supported Pd/Ni-heteropoly acid bifunctional catalysts. React. Kinet. Mech. Catal..

[39] Zhu Y.-P. (1998). Chinese Materia Medica: Chemistry, Pharmacology and Applications.

[40] Tan F., Chen Y., Tan X., Ma Y., Peng Y. (2017). Chinese materia medica used in medicinal diets. J. Ethnopharmacol..

[41] Chung B.H., Lee H.-y., Lee J.S., Young C.Y. (2006). Perillyl alcohol inhibits the expression and function of the androgen receptor in human prostate cancer cells. Cancer Lett..

[42] Cho B.O., Jin C.H., Park Y.D., Ryu H.W., Byun M.W., Seo K.I., Jeong I.Y. (2011). Isoegomaketone induces apoptosis through caspase-dependent and caspase-independent pathways in human DLD1 cells. Biosci. Biotechnol. Biochem..

[43] Jin C.H., So Y.K., Han S.N., Kim J.-B. (2016). Isoegomaketone upregulates heme oxygenase-1 in RAW264. 7 cells via ROS/p38 MAPK/Nrf2 pathway. Biomol. Ther..

[44] Bumblauskiene L., Jakstas V., Janulis V., Mazdzieriene R., Ragazinskiene O. (2009). Preliminary analysis on essential oil composition of Perilla L. cultivated in Lithuania. Acta Pol. Pharm..

[45] Tian J., Wang Y., Lu Z., Sun C., Zhang M., Zhu A., Peng X. (2016). Perillaldehyde, a promising antifungal agent used in food preservation, triggers apoptosis through a metacaspase-dependent pathway in *Aspergillus flavus*. J. Agric. Food Chem..

[46] Xu L., Li Y., Fu Q., Ma S. (2014). Perillaldehyde attenuates cerebral ischemia–reperfusion injury-triggered overexpression of inflammatory cytokines via modulating Akt/JNK pathway in the rat brain cortex. Biochem. Biophys. Res. Commun..

[47] Loutrari H., Hatziapostolou M., Skouridou V., Papadimitriou E., Roussos C., Kolisis F.N., Papapetropoulos A. (2004). Perillyl alcohol is an angiogenesis inhibitor. J. Pharmacol. Exp. Ther..

[48] Jin C.H., Lee H.J., Park Y.D., Choi D.S., Kim D.S., Kang S.-Y., Seo K.-I., Jeong I.Y. (2010). Isoegomaketone inhibits lipopolysaccharide-induced nitric oxide production in RAW 264.7 macrophages through the heme oxygenase-1 induction and inhibition of the interferon-β-STAT-1 pathway. J. Agric. Food Chem..

[49] Park H.C., So Y.K., Kim J.B., Jin C.H., Yuk H.S. (2016). Comparison of anti-inflammatory activity of extracts with supercritical carbon dioxide from radiation mutant *Perilla frutescens* (L.) Britton and wild-type. J. Radiat. Ind..

[50] Nam B., So Y., Kim H.-Y., Kim J.-B., Jin C.H., Han A.-R. (2017). A new monoterpene from the leaves of a radiation mutant cultivar of *Perilla frutescens* var. crispa with inhibitory activity on LPS-induced NO production. Molecules.

[51] Hájíček J. (2012). Recent developments in syntheses of the post-secodine indole alkaloids. Part III: Rearranged alkaloid types. Collect. Czechoslov. Chem. Commun..

[52] Banerji A., Majumder P., Chatterjee A. (1970). Occurrence of geissoschizine and other minor biogenetically related alkaloids in *Rhazya stricta*. Phytochemistry.

[53] Sirindil F., Weibel J.-M., Pale P., Blanc A. (2019). Total synthesis of Rhazinilam through gold-catalyzed cycloisomerization—Sulfonyl migration and palladium-catalyzed Suzuki—Miyaura coupling of pyrrolyl sulfonates. Org. Lett..

[54] Zheng Y., Yue B.-B., Wei K., Yang Y.-R. (2018). Short synthesis of the monoterpene indole alkaloid (±)-arbornamine. J. Org. Chem..

[55] Lim K.-H., Hiraku O., Komiyama K., Koyano T., Hayashi M., Kam T.-S. (2007). Biologically active indole alkaloids from *Kopsia arborea*. J. Nat. Prod..

[56] Tooriyama S., Mimori Y., Wu Y., Kogure N., Kitajima M., Takayama H. (2017). Asymmetric total synthesis of pentacyclic indole alkaloid andranginine and absolute configuration of natural product isolated from *Kopsia arborea*. Org. Lett..

[57] Tokuda R., Okamoto Y., Koyama T., Kogure N., Kitajima M., Takayama H. (2016). Asymmetric total synthesis of Kopsiyunnanine K, A monoterpenoid indole alkaloid with a rearranged skeleton. Org. Lett..

[58] Knölker H. (2008). KR Reddy in The Alkaloids.

[59] Mancebo F., Hilje L., Mora G.A., Castro V.H., Salazar R. (2001). Biological activity of *Ruta chalepensis* (Rutaceae) and *Sechium pittieri* (Cucurbitaceae) extracts on *Hypsipyla grandella* (Lepidoptera: Pyralidae) larvae. Rev. Biol. Trop..

[60] Iyer D., Devi U. (2008). Phyto-pharmacology of *Murraya koenigii* (L.). Pharmacogn. Rev..

[61] Bandaranayake W., Begley M., Brown B.O., Clarke D.G., Crombie L., Whiting D.A. (1974). Syntheses of acridone and carbazole alkaloids involving pyridine-catalysed chromen formation: Crystal and molecular structure of dibromocannibicyclol and its bearing on the structures of the ‘cyclol’ alkaloids. J. Chem. Soc. Perkin Trans.

[62] Wu T.-S., Wang M.-L., Wu P.-L., Ito C., Furukawa H. (1996). Carbazole alkaloids from the leaves of *Murraya euchrestifolia*. Phytochemistry.

[63] Wu T.-S., Wang M.-L., Wu P.-L. (1996). Seasonal variations of carbazole alkaloids in *Murraya euchrestifolia*. Phytochemistry.

[64] Tan S.-P., Ali A.M., Nafiah M.A., Awang K., Ahmad K. (2015). Isolation and cytotoxic investigation of new carbazole alkaloids from *Murraya koenigii* (Linn.) Spreng. Tetrahedron.

[65] Gassner C., Hesse R., Schmidt A.W., Knölker H.-J. (2014). Total synthesis of the cyclic monoterpenoid pyrano [3,2-*a*] carbazole alkaloids derived from 2-hydroxy-6-methylcarbazole. Org. Biomol. Chem..

[66] Kam T.S., Choo Y.M. (2004). Angustilodine, an unusual pentacyclic indole alkaloid from Alstonia. Helv. Chim. Acta.

[67] Koyama K., Hirasawa Y., Zaima K., Hoe T.C., Chan K.-L., Morita H. (2008). Alstilobanines A–E, new indole alkaloids from *Alstonia angustiloba*. Bioorg. Med. Chem..

[68] Feng Y., Majireck M.M., Weinreb S.M. (2014). Total syntheses of the monoterpene indole alkaloids (±)-alstilobanine A and E and (±)-angustilodine. J. Org. Chem..

[69] Liang-Yan L., Zheng-Hui L., Ji-Kai L. (2013). A new menthane-type monoterpene from *Pleurotus eryngii*. Chin. J. Nat. Med..

[70] Kobayashi T., Ishida M., Imaida K., Abe H., Ito H. (2017). Total synthesis of pleurolactone. Tetrahedron Lett..

[71] Yang X.W., Li S.M., Li Y.L., Xia J.H., Wu L., Shen Y.H., Tian J.M., Wang N., Liu Y., Zhang W.D. (2010). Abiespiroside A, an Unprecedented Sesquiterpenoid Spirolactone with a 6/6/5 Ring System from Abies Delavayi.

[72] Hu C.L., Xiong J., Li J.Y., Gao L.X., Wang W.X., Cheng K.J., Yang G.X., Li J., Hu J.F. (2016). Rare Sesquiterpenoids from the Shed Trunk Barks of the Critically Endangered Plant *Abies Beshanzuensis* and Their Bioactivities.

[73] Davis D.C., Hoch D.G., Wu L., Abegg D., Martin B.S., Zhang Z.-Y., Adibekian A., Dai M. (2018). Total synthesis, biological evaluation, and target identification of Rare Abies Sesquiterpenoids. J. Am. Chem. Soc..

[74] Lim T. (2016). Cyperus rotundus. Edible Medicinal and Non-Medicinal Plants.

[75] Kilani S., Sghaier M.B., Limem I., Bouhlel I., Boubaker J., Bhouri W., Skandrani I., Neffatti A., Ammar R.B., Dijoux-Franca M.G. (2008). In vitro evaluation of antibacterial, antioxidant, cytotoxic and apoptotic activities of the tubers infusion and extracts of *Cyperus rotundus*. Bioresour. Technol..

[76] Klahn P., Duschek A., Liébert C.M., Kirsch S.F. (2012). Total synthesis of (+)-cyperolone. Org. Lett..

[77] Ma S.-G., Li M., Lin M.-B., Li L., Liu Y.-B., Qu J., Li Y., Wang X.-J., Wang R.-B., Xu S. (2017). Illisimonin A, a Caged Sesquiterpenoid with a Tricyclo [5.2.1.0^1,6^] decane Skeleton from the Fruits of Illicium simonsii. Org. Lett..

[78] Burns A.S., Rychnovsky S.D. (2019). Total Synthesis and Structure Revision of (−)-Illisimonin A, a Neuroprotective Sesquiterpenoid from the Fruits of *Illicium simonsii*. J. Am. Chem. Soc..

[79] Jiao W.-H., Huang X.-J., Yang J.-S., Yang F., Piao S.-J., Gao H., Li J., Ye W.-C., Yao X.-S., Chen W.-S. (2012). Dysidavarones A–D, new sesquiterpene quinones from the marine sponge Dysidea avara. Org. Lett..

[80] Lee S., Wang Q. (2007). Recent development of small molecular specific inhibitor of protein tyrosine phosphatase 1B. Med. Res. Rev..

[81] Fukui Y., Narita K., Katoh T. (2014). Enantioselective total synthesis of dysidavarone A, a novel sesquiterpenoid quinone from the marine sponge Dysidea avara. Chem.–Eur. J..

[82] Chung H.-M., Wang W.-H., Hwang T.-L., Fang L.-S., Wen Z.-H., Chen J.-J., Wu Y.-C., Sung P.-J. (2014). Rumphellaoic acid A, a novel sesquiterpenoid from the formosan gorgonian coral *Rumphella antipathies*. Mar. Drugs.

[83] Chung H.-M., Chen Y.-H., Lin M.-R., Su J.-H., Wang W.-H., Sung P.-J. (2010). Rumphellaone A, a novel caryophyllane-related derivative from the gorgonian coral *Rumphella antipathies*. Tetrahedron Lett..

[84] Ranieri B., Obradors C., Mato M., Echavarren A.M. (2016). Synthesis of rumphellaone A and hushinone by a gold-catalyzed [2+ 2] cycloaddition. Org. Lett..

[85] Rasmussen U., Sandberg F. (1978). Thapsigargine and thapsigargicine, two new histamine liberators from *Thapsia garganica* L. Acta Pharm. Suec..

[86] Ali H., Christensen S.B., Foreman J., Pearce F., Piotrowski W., Thastrup O. (1985). The ability of thapsigargin and thapsigargicin to activate cells involved in the inflammatory response. Br. J. Pharmacol..

[87] Davidson G.A., Varhol R.J. (1995). Kinetics of thapsigargin-Ca2+-ATPase (sarcoplasmic reticulum) interaction reveals a two-step binding mechanism and picomolar inhibition. J. Biol. Chem..

[88] Denmeade S.R., Mhaka A.M., Rosen D.M., Brennen W.N., Dalrymple S., Dach I., Olesen C., Gurel B., DeMarzo A.M., Wilding G. (2012). Engineering a prostate-specific membrane antigen–activated tumor endothelial cell prodrug for cancer therapy. Sci. Transl. Med..

[89] Chu H., Smith J.M., Felding J., Baran P.S. (2017). Scalable synthesis of (−)-thapsigargin. ACS Cent. Sci..

[90] Kubo M., Nishikawa Y., Harada K., Oda M., Huang J.-M., Domon H., Terao Y., Fukuyama Y. (2015). Tetranorsesquiterpenoids and santalane-type sesquiterpenoids from *Illicium lanceolatum* and their antimicrobial activity against the oral pathogen Porphyromonas gingivalis. J. Nat. Prod..

[91] Lee B., Bohmann J., Reeves T., Levenson C., Risinger A.L. (2015). α-and β-Santalols directly interact with tubulin and cause mitotic arrest and cytotoxicity in oral cancer cells. J. Nat. Prod..

[92] Lomba L., Afarinkia K., Vinader V. (2019). A new route to tricyclane sesquiterpenoids: Total synthesis of α-ekasantalic acid. Org. Biomol. Chem..

[93] Bommareddy A., Knapp K., Nemeth A., Steigerwalt J., Landis T., Vanwert A.L., Gorijavolu H.P., Dwivedi C. (2018). Alpha-Santalol, a component of sandalwood oil inhibits migration of breast cancer cells by targeting the β-catenin pathway. Anticancer Res..

[94] Schlosser M., Zhong G.F. (1993). A shortcut to a-santalol. Tetrahedron Lett..

[95] Ochi T., Shibata H., Higuti T., Kodama K.-h., Kusumi T., Takaishi Y. (2005). Anti-Helicobacter pylori Compounds from *Santalum album*. J. Nat. Prod..

[96] Matsuo Y., Mimaki Y. (2012). α-Santalol derivatives from *Santalum album* and their cytotoxic activities. Phytochemistry.

[97] Wang L., Zhang C.-F., Wang Z.-T., Zhang M., Xu L.-S. (2009). Five new compounds from *Dendrobium crystallinum*. J. Asian Nat. Prod. Res..

[98] Rakotonandrasana O.L., Raharinjato F.H., Rajaonarivelo M., Dumontet V., Martin M.-T.R.S., Bignon J., Rasoanaivo P. (2010). Cytotoxic 3,4-seco-atisane diterpenoids from Croton barorum and Croton goudotii. J. Nat. Prod..

[99] Drummond G.J., Grant P.S., Brimble M.A. (2021). ent-Atisane diterpenoids: Isolation, structure and bioactivity. Nat. Prod. Rep..

[100] Moremi M.P., Makolo F., Viljoen A.M., Kamatou G.P. (2021). A review of biological activities and phytochemistry of six ethnomedicinally important South African Croton species. J. Ethnopharmacol..

[101] Finkbeiner P., Murai K., Röpke M., Sarpong R. (2017). Total synthesis of terpenoids employing a “benzannulation of carvone” strategy: Synthesis of (−)-crotogoudin. J. Am. Chem. Soc..

[102] Surowiak A.K., Balcerzak L., Lochyński S., Strub D.J. (2021). Biological activity of selected natural and synthetic terpenoid lactones. Int. J. Mol. Sci..

[103] Rosenbaum L.C., Häfner M., Gaich T. (2021). Total synthesis of the diterpene waihoensene. Angew. Chem..

[104] Clarke D.B., Hinkley S.F., Weavers R.T. (1997). Waihoensene. A new laurenene-related diterpene from Podocarpus totara var waihoensis. Tetrahedron Lett..

[105] Yang Z. (2021). Navigating the Pauson–Khand Reaction in Total Syntheses of Complex Natural Products. Acc. Chem. Res..

[106] Peng C., Arya P., Zhou Z., Snyder S.A. (2020). A Concise Total Synthesis of (+)-Waihoensene Guided by Quaternary Center Analysis. Angew. Chem..

[107] Xiong L., Zhou Q.-M., Zou Y., Chen M.-H., Guo L., Hu G.-Y., Liu Z.-H., Peng C. (2015). Leonuketal, a spiroketal diterpenoid from *Leonurus japonicus*. Org. Lett..

[108] Carreira E.M., Freis M. (2021). Synthesis of (±)-Leonuketal. Synfacts.

[109] Nakadate S., Nozawa K., Horie H. (2011). New type indole diterpene, eujindoles, from eupenicillium javanicum. Heterocycles.

[110] Bian M., Wang Z., Xiong X., Sun Y., Matera C., Nicolaou K., Li A. (2012). Total syntheses of anominine and tubingensin A. J. Am. Chem. Soc..

[111] Xu K., Li G., Zhu R., Xie F., Li Y., Yang W., Xu L., Shen T., Zhao Z., Lou H. (2020). Polyketides from the endolichenic fungus *Eupenicillium javanicum* and their anti-inflammatory activities. Phytochemistry.

[112] Lu Z., Li H., Bian M., Li A. (2015). Total synthesis of epoxyeujindole A. J. Am. Chem. Soc..

[113] Jiang H.-Y., Wang W.-G., Zhou M., Wu H.-Y., Zhan R., Li X.-N., Du X., Li Y., Pu J.-X., Sun H.-D. (2013). Enmein-type 6, 7-seco-ent-kauranoids from Isodon sculponeatus. J. Nat. Prod..

[114] Moritz B.J., Mack D.J., Tong L., Thomson R.J. (2014). Total synthesis of the isodon diterpene sculponeatin N. Angew. Chem..

[115] Senneville S., Beaulieu J., Daoust G., Deslauriers M., Bousquet J. (2001). Evidence for low genetic diversity and metapopulation structure in Canada yew (*Taxus canadensis*): Considerations for conservation. Can. J. For. Res..

[116] Huo C.-H., Su X.-H., Wang Y.-F., Zhang X.-P., Shi Q.-W., Kiyota H. (2007). Canataxpropellane, a novel taxane with a unique polycyclic carbon skeleton (tricyclotaxane) from the needles of Taxus canadensis. Tetrahedron Lett..

[117] Schneider F., Samarin K., Zanella S., Gaich T. (2020). Total synthesis of the complex taxane diterpene canataxpropellane. Science.

[118] Flaschenträger B., von Falkenhausen F.F. (1934). Über den Giftstoff im Krotonöl. II. Zur Konstitution von Krotophorbolon. Justus Liebigs Ann. Der Chem..

[119] Thielmann H.W., Hecker E. (1969). Zur Chemie des Phorbols, XIV Die Flaschenträger-Reaktion. Justus Liebigs Ann. Der Chem..

[120] Wang H.-B., Chu W.-J., Wang Y., Ji P., Wang Y.-B., Yu Q., Qin G.-W. (2010). Diterpenoids from the roots of *Euphorbia fischeriana*. J. Asian Nat. Prod. Res..

[121] Ma Q.-G., Liu W.-Z., Wu X.-Y., Zhou T.-X., Qin G.-W. (1997). Diterpenoids from *Euphorbia fischeriana*. Phytochemistry.

[122] Asaba T., Katoh Y., Urabe D., Inoue M. (2015). Total synthesis of crotophorbolone. Angew. Chem. Int. Ed..

[123] Tang P., Chen Q.-H., Wang F.-P. (2009). Atropurpuran, a novel diterpene with an unprecedented pentacyclic cage skeleton, from *Aconitum hemsleyanum* var. atropurpureum. Tetrahedron Lett..

[124] Weber M., Owens K., Sarpong R. (2015). Atropurpuran—Missing biosynthetic link leading to the hetidine and arcutine C20-diterpenoid alkaloids or an oxidative degradation product?. Tetrahedron Lett..

[125] Verma S., Ojha S., Raish M. (2010). Anti-inflammatory activity of *Aconitum heterophyllum* on cotton pellet-induced granuloma in rats. J. Med. Plants Res..

[126] Xie S., Chen G., Yan H., Hou J., He Y., Zhao T., Xu J. (2019). 13-Step total synthesis of atropurpuran. J. Am. Chem. Soc..

[127] Kosugi K., Sakai J.-i., Zhang S., Watanabe Y., Sasaki H., Suzuki T., Hagiwara H., Hirata N., Hirose K., Ando M. (2000). Neutral taxoids from *Taxus cuspidata* as modulators of multidrug-resistant tumor cells. Phytochemistry.

[128] Wang Y.-F., Shi Q.-W., Dong M., Kiyota H., Gu Y.-C., Cong B. (2011). Natural taxanes: Developments since 1828. Chem. Rev..

[129] Bailey D.T. (1995). Taxane Anticancer Agents Basic Science and Current Status Edited by Gunda I. Georg (University of Kansas), Thomas T. Chen (University of Tennessee), Iwao Ojima (State University of New York at Stony Brook), and Dolatrai M. Vyas (Bristol-Myers Squibb). ACS Symposium Series 583. 15× 22.5 cm. $99.95.

[130] Imamura Y., Yoshioka S., Nagatomo M., Inoue M. (2019). Total Synthesis of 1-Hydroxytaxinine. Angew. Chem..

[131] Uchida I., Ando T., Fukami N., Yoshida K., Hashimoto M., Tada T., Koda S., Morimoto Y. (1987). The structure of vinigrol, a novel diterpenoid with antihypertensive and platelet aggregation-inhibitory activities. J. Org. Chem..

[132] Maimone T.J., Baran P.S. (2007). Modern synthetic efforts toward biologically active terpenes. Nat. Chem. Biol..

[133] Ando T., Tsurumi Y., Ohata N., Uchida I., Yoshida K., Okuhara M. (1988). Vinigrol, a Novel Antihypertensive and Platelet Aggregation Inhibitory Agent Produced by a Fungus, Virgaria Nigra I. Taxonomy, Fermentation, Isolation, Physico-Chemical and Biological Properties. J. Antibiot..

[134] Min L., Lin X., Li C.-C. (2019). Asymmetric total synthesis of (−)-vinigrol. J. Am. Chem. Soc..

[135] Sun H.-F., Li X.-M., Meng L., Cui C.-M., Gao S.-S., Li C.-S., Huang C.-G., Wang B.-G. (2012). Asperolides A–C, tetranorlabdane diterpenoids from the marine alga-derived endophytic fungus Aspergillus wentii EN-48. J. Nat. Prod..

[136] Chinou I. (2005). Labdanes of natural origin-biological activities (1981–2004). Curr. Med. Chem..

[137] Singh M., Pal M., Sharma R. (1999). Biological activity of the *labdane diterpenes*. Planta Med..

[138] Miyazawa M., Shimamura H., Nakamura S.-I., Kameoka H. (1995). Antimutagenic activity of (+)-polyalthic acid from *Vitex rotundiforia*. J. Agric. Food Chem..

[139] Itokawa H., Morita H., Katou I., Takeya K., Cavalheiro A.J., de Oliveira R.C., Ishige M., Motidome M. (1988). Cytotoxic diterpenes from the rhizomes of *Hedychium coronarium*. Planta Med..

[140] Morita H., Itokawa H. (1988). Cytotoxic and antifungal diterpenes from the seeds of *Alpinia galanga*. Planta Med..

[141] Jeker O.F., Kravina A.G., Carreira E.M. (2013). Total Synthesis of (+)-Asperolide C by Iridium-Catalyzed Enantioselective Polyene Cyclization. Angew. Chem..

[142] Rodriguez B.E. (2017). Contribución al estudio químico sistemático del género Salvia (Lamiaceae) en México. Ph.D. Thesis.

[143] Roth B.L., Baner K., Westkaemper R., Siebert D., Rice K.C., Steinberg S., Ernsberger P., Rothman R.B. (2002). Salvinorin A: A potent naturally occurring nonnitrogenous κ opioid selective agonist. Proc. Natl. Acad. Sci. USA.

[144] Cunningham C.W., Rothman R.B., Prisinzano T.E. (2011). Neuropharmacology of the naturally occurring κ-opioid hallucinogen salvinorin A. Pharmacol. Rev..

[145] Fajemiroye J.O., Prabhakar P.R., da Cunha L.C., Costa E.A., Zjawiony J.K. (2017). 22-azidosalvinorin A exhibits antidepressant-like effect in mice. Eur. J. Pharmacol..

[146] Wang Y., Metz P. (2018). Total Synthesis of the Neoclerodane Diterpene Salvinorin A via an Intramolecular Diels–Alder Strategy. Org. Lett..

[147] Lei C., Huang S.-X., Chen J.-J., Yang L.-B., Xiao W.-L., Chang Y., Lu Y., Huang H., Pu J.-X., Sun H.-D. (2008). Propindilactones E−J, Schiartane Nortriterpenoids from *Schisandra propinqua* var. propinqua. J. Nat. Prod..

[148] Li R.-T., Han Q.-B., Zheng Y.-T., Wang R.-R., Yang L.-M., Lu Y., Sang S.-Q., Zheng Q.-T., Zhao Q.-S., Sun H.-D. (2005). Structure and anti-HIV activity of micrandilactones B and C, new nortriterpenoids possessing a unique skeleton from *Schisandra micrantha*. Chem. Commun..

[149] You L., Liang X.-T., Xu L.-M., Wang Y.-F., Zhang J.-J., Su Q., Li Y.-H., Zhang B., Yang S.-L., Chen J.-H. (2015). Asymmetric total synthesis of Propindilactone G. J. Am. Chem. Soc..

[150] Ebada S.S., Lin W., Proksch P. (2010). Bioactive sesterterpenes and triterpenes from marine sponges: Occurrence and pharmacological significance. Mar. Drugs.

[151] McKee T.C., Bokesch H.R., McCormick J.L., Rashid M.A., Spielvogel D., Gustafson K.R., Alavanja M.M., Cardellina J.H., Boyd M.R. (1997). Isolation and characterization of new anti-HIV and cytotoxic leads from plants, marine, and microbial organisms. J. Nat. Prod..

[152] Boyko Y.D., Huck C.J., Sarlah D. (2019). Total synthesis of isomalabaricane triterpenoids. J. Am. Chem. Soc..

[153] Kunjumon R., Johnson A.J., Baby S. (2022). Phytomedicine Plus.

[154] Madhujith T., Sivakanthan S. (2016). 7 Centella. Leafy Med. Herbs.

[155] Wen X., Sun H., Liu J., Cheng K., Zhang P., Zhang L., Hao J., Zhang L., Ni P., Zographos S.E. (2008). Naturally occurring pentacyclic triterpenes as inhibitors of glycogen phosphorylase: Synthesis, structure–activity relationships, and X-ray crystallographic studies. J. Med. Chem..

[156] Shao W., Cao X., Shen L., Zhang F., Yu B. (2017). A convergent synthesis of the triterpene saponin asiaticoside. Asian J. Org. Chem..

[157] Meng F.-Y., Sun J.-X., Li X., Yu H.-Y., Li S.-M., Ruan H.-L. (2011). Schiglautone A, a new tricyclic triterpenoid with a unique 6/7/9-fused skeleton from the stems of *Schisandra glaucescens*. Org. Lett..

[158] Gansäuer A., Bluhm H., Rinker B., Narayan S., Schick M., Lauterbach T., Pierobon M. (2003). Reagent-Controlled Stereoselectivity in Titanocene-Catalyzed Epoxide Openings: Reductions and Intermolecular Additions to α, β-Unsaturated Carbonyl Compounds. Chem.–Eur. J..

[159] Wang H., Zhang X., Tang P. (2017). Total syntheses of schilancidilactones A and B, schilancitrilactone A, and 20-epi-schilancitrilactone A via late-stage nickel-catalyzed cross coupling. Chem. Sci..

[160] Xia Y.-G., Yang B.-Y., Kuang H.-X. (2015). Schisandraceae triterpenoids: A review. Phytochem. Rev..

[161] Liu H., Qi Y., Xu L., Peng Y., Zhang B., Xiao P. (2012). Ethno-pharmacological investigation of *Schisandraceae* plants in China. Zhongguo Zhong Yao Za Zhi = Zhongguo Zhongyao Zazhi = China J. Chin. Mater. Med..

[162] Gan S.-R., Du W., Wang X.-F. (2022). Functional Differentiation of Floral Color and Scent in Gall Midge Pollination: A Study of a *Schisandraceae* Plant. Plants.

[163] Ma B., Zhao Y., He C., Ding H. (2018). Total synthesis of an atropisomer of the Schisandra triterpenoid schiglautone A. Angew. Chem..

[164] Zhou Z.-W., Yin S., Zhang H.-Y., Fu Y., Yang S.-P., Wang X.-N., Wu Y., Tang X.-C., Yue J.-M. (2008). Walsucochins A and B with an unprecedented skeleton isolated from Walsura cochinchinensis. Org. Lett..

[165] Xu S., Gu J., Li H., Ma D., Xie X., She X. (2014). Enantioselective Total Synthesis of (−)-Walsucochin B. Org. Lett..

[166] Xiao W.-L., Yang L.-M., Gong N.-B., Wu L., Wang R.-R., Pu J.-X., Li X.-L., Huang S.-X., Zheng Y.-T., Li R.-T. (2006). Rubriflordilactones A and B, Two Novel Bisnortriterpenoids from *Schisandra* r ubriflora and Their Biological Activities. Org. Lett..

[167] Li J., Yang P., Yao M., Deng J., Li A. (2014). Total synthesis of rubriflordilactone A. J. Am. Chem. Soc..

[168] Sadler I.H., Simpson T.J. (1992). Determination by NMR methods of the structure and stereochemistry of astellatol, a new and unusual sesterterpene. Magn. Reson. Chem..

[169] Wang L., Yang B., Lin X.-P., Zhou X.-F., Liu Y. (2013). Sesterterpenoids. Nat. Prod. Rep..

[170] Zhao N., Yin S., Xie S., Yan H., Ren P., Chen G., Chen F., Xu J. (2018). Total synthesis of astellatol. Angew. Chem. Int. Ed..

[171] Senatore F., Defeo V., Zhou Z. (1991). Chemical-Constituents of Gentianella-Alborosea (GILG) Fabris. Ann. Chim..

[172] Wang Y., Dreyfuss M., Ponelle M., Oberer L., Riezman H. (1998). A glycosylphosphatidylinositol-anchoring inhibitor with an unusual tetracarbocyclic sesterterpene skeleton from the fungus Codinaea simplex. Tetrahedron.

[173] Hog D.T., Huber F.M., Mayer P., Trauner D. (2014). The Total Synthesis of (−)-Nitidasin. Angew. Chem. Int. Ed..

[174] Tempesta M.S., Iwashita T., Miyamoto F., Yoshihara K., Naya Y. (1983). A new class of sesterterpenoids from the secretion of ceroplastes rubens (Coccidae). J. Chem. Soc. Chem. Commun..

[175] Noda T., Kitamura C., Takahashi S., Takagi K., Kashio T., Tanaka M. (1982). Host Selection Behavior of Anicetus beneficus ISHII et YASUMATSU (Hymenoptera: Encyrtidae): I. Ovipositional Behavior for the Natural Host Ceroplastes rubens MASKELL (Hemiptera: Coccidae). Appl. Entomol. Zool..

[176] Pour P.M., Behzad S., Asgari S., Khankandi H.P., Farzaei M.H. (2020). Sesterterpenoids. Recent Advances in Natural Products Analysis.

[177] Mak J.Y., Pouwer R.H., Williams C.M. (2014). Natural Products with Anti-Bredt and Bridgehead Double Bonds. Angew. Chem. Int. Ed..

[178] Liu X., Liu J., Wu J., Huang G., Liang R., Chung L.W., Li C.-C. (2019). Asymmetric total synthesis of cerorubenic acid-III. J. Am. Chem. Soc..

[179] Daoust J., Fontana A., Merchant C.E., de Voogd N.J., Patrick B.O., Kieffer T.J., Andersen R.J. (2010). Ansellone A, a sesterterpenoid isolated from the nudibranch Cadlina luteromarginata and the sponge *Phorbas* sp., activates the cAMP signaling pathway. Org. Lett..

[180] Wang M., Tietjen I., Chen M., Williams D.E., Daoust J., Brockman M.A., Andersen R.J. (2016). Sesterterpenoids isolated from the sponge *Phorbas* sp. activate latent HIV-1 provirus expression. J. Org. Chem..

[181] Yanagihara M., Murai K., Kishimoto N., Abe T., Misumi S., Arisawa M. (2021). Total synthesis and biological evaluation of the potent HIV latency-reversing agent Ansellone A and its analogues. Org. Lett..

[182] Huang X., Song L., Xu J., Zhu G., Liu B. (2013). Asymmetric total synthesis of leucosceptroid B. Angew. Chem. Int. Ed..

[183] Luo S.H., Luo Q., Niu X.M., Xie M.J., Zhao X., Schneider B., Gershenzon J., Li S.H. (2010). Glandular trichomes of *Leucosceptrum canum* harbor defensive sesterterpenoids. Angew. Chem..

[184] Hugelshofer C.L., Magauer T. (2014). A General Entry to Antifeedant Sesterterpenoids: Total Synthesis of (+)-Norleucosceptroid A,(−)-Norleucosceptroid B, and (−)-Leucosceptroid K. Angew. Chem..

[185] Luo S.-H., Hua J., Niu X.-M., Liu Y., Li C.-H., Zhou Y.-Y., Jing S.-X., Zhao X., Li S.-H. (2013). Defense sesterterpenoid lactones from *Leucosceptrum canum*. Phytochemistry.

[186] Guo S., Liu J., Ma D. (2015). Total synthesis of leucosceptroids A and B. Angew. Chem. Int. Ed..

[187] Chen Y., Zhao J., Li S., Xu J. (2019). Total synthesis of sesterterpenoids. Nat. Prod. Rep..

[188] Ueoka R., Nakao Y., Fujii S., van Soest R.W., Matsunaga S. (2008). Aplysinoplides A−C, cytotoxic sesterterpenes from the marine sponge Aplysinopsis digitata. J. Nat. Prod..

[189] Evidente A., Kornienko A., Lefranc F., Cimmino A., Dasari R., Evidente M., Mathieu V., Kiss R. (2015). Sesterterpenoids with anticancer activity. Curr. Med. Chem..

[190] Zhang C., Liu Y. (2015). Targeting cancer with sesterterpenoids: The new potential antitumor drugs. J. Nat. Med..

[191] Kutsumura N., Matsuo K., Saito T. (2015). Total synthesis of aplysinoplide B. Tetrahedron Lett..

[192] Chen K., Zhang X., Sun W., Liu J., Yang J., Chen C., Liu X., Gao L., Wang J., Li H. (2017). Manginoids A–G: Seven monoterpene–shikimate-conjugated meroterpenoids with a spiro ring system from *Guignardia mangiferae*. Org. Lett..

[193] Hamilton B.S., Himmelsbach F., Nar H., Schuler-Metz A., Krosky P., Guo J., Guo R., Meng S., Zhao Y., Lala D.S. (2015). Pharmacological characterization of the selective 11β-hydroxysteroid dehydrogenase 1 inhibitor, BI 135585, a clinical candidate for the treatment of type 2 diabetes. Eur. J. Pharmacol..

[194] Zhang Y.A., Milkovits A., Agarawal V., Taylor C.A., Snyder S.A. (2021). Total Synthesis of the Meroterpenoid Manginoid A as Fueled by a Challenging Pinacol Coupling and Bicycle-forming Etherification. Angew. Chem..

[195] Yang X.-W., Li Y.-P., Su J., Ma W.-G., Xu G. (2016). Hyperjapones A–E, terpenoid polymethylated acylphloroglucinols from *Hypericum japonicum*. Org. Lett..

[196] Wu Q.-L., Wang S.-P., Du L.-J., Yang J.-S., Xiao P.-G. (1998). Xanthones from *Hypericum japonicum* and *H. henryi*. Phytochemistry.

[197] Hu L., Zhang Y., Zhu H., Liu J., Li H., Li X.-N., Sun W., Zeng J., Xue Y., Zhang Y. (2016). Filicinic acid based meroterpenoids with anti-Epstein–Barr virus activities from *Hypericum japonicum*. Org. Lett..

[198] Lam H.C., Spence J.T., George J.H. (2016). Biomimetic total synthesis of hyperjapones A–E and hyperjaponols A and C. Angew. Chem..

[199] Ni B., Dong X., Fu J., Yin X., Lin L., Xia Z., Zhao Y., Xue D., Yang C., Ni J. (2015). Phytochemical and biological properties of *Ajuga decumbens* (Labiatae): A review. Trop. J. Pharm. Res..

[200] Bedi A., Adholeya A., Deshmukh S.K. (2018). Novel anticancer compounds from endophytic fungi. Curr. Biotechnol..

[201] Lin T., Wang G., Shan W., Zeng D., Ding R., Jiang X., Zhu D., Liu X., Yang S., Chen H. (2014). Myrotheciumones: Bicyclic cytotoxic lactones isolated from an endophytic fungus of *Ajuga decumbens*. Bioorg. Med. Chem. Lett..

[202] Begum S., Chakraborty T.K. (2021). Cp2TiCl-Mediated Reductive Cyclization: Total Synthesis of Pestalotiolactone A, Myrotheciumone A, and Scabrol A. J. Org. Chem..

[203] Wilson M.C., Nam S.-J., Gulder T.A., Kauffman C.A., Jensen P.R., Fenical W., Moore B.S. (2011). Structure and biosynthesis of the marine streptomycete ansamycin ansalactam A and its distinctive branched chain polyketide extender unit. J. Am. Chem. Soc..

[204] Sakoulas G., Nam S.-J., Loesgen S., Fenical W., Jensen P.R., Nizet V., Hensler M. (2012). Novel bacterial metabolite merochlorin A demonstrates in vitro activity against multi-drug resistant methicillin-resistant *Staphylococcus aureus*. PLoS ONE.

[205] Brandstätter M., Freis M., Huwyler N., Carreira E.M. (2019). Total Synthesis of (−)-Merochlorin A. Angew. Chem..

[206] Yang Z., Yi Y., Gao C., Hou D., Hu J., Zhao M. (2010). Isolation of inulin-type oligosaccharides from Chinese traditional medicine: Morinda officinalis How and their characterization using ESI-MS/MS. J. Sep. Sci..

[207] Yang Y., Shu H., Min Z. (1992). Anthraquinones isolated from *Morinda officinalis* and *Damnacanthus indicus*. Yao Xue Xue Bao= Acta Pharm. Sin..

[208] Wang M.-Y., West B.J., Jensen C.J., Nowicki D., Su C., Palu A.K., Anderson G. (2002). Morinda citrifolia (Noni): A literature review and recent advances in Noni research. Acta Pharmacol. Sin..

[209] Wu Y.-B., Zheng C.-J., Qin L.-P., Sun L.-N., Han T., Jiao L., Zhang Q.-Y., Wu J.-Z. (2009). Antiosteoporotic activity of anthraquinones from Morinda officinalis on osteoblasts and osteoclasts. Molecules.

[210] Zhang H., Li J., Li G., Wang D., Zhu L., Yang D. (2009). Caracterización estructural y actividad antifatiga de los polisacáridos de las raíces de Morinda officinalis. Int. J. Biol. Macromol..

[211] Soon Y., Tan B. (2002). Evaluation of the hypoglycemic and anti-oxidant activities of *Morinda officinalis* in streptozotocin-induced diabetic rats. Singap. Med. J..

[212] Zhang Z.-Q., Yuan L., Yang M., Luo Z.-P., Zhao Y.-M. (2002). The effect of Morinda officinalis How, a Chinese traditional medicinal plant, on the DRL 72-s schedule in rats and the forced swimming test in mice. Pharmacol. Biochem. Behav..

[213] Liu H.-X., Tan H.-B., Chen K., Zhao L.-Y., Chen Y.-C., Li S.-N., Li H.-H., Zhang W.-M. (2019). Cytosporins A–D, novel benzophenone derivatives from the endophytic fungus Cytospora rhizophorae A761. Org. Biomol. Chem..

[214] Sanchez-Ferrando F., San-Martin A. (1995). Epitaondiol: The first polycyclic meroditerpenoid containing two fused six-membered rings forced into the twist-boat conformation. J. Org. Chem..

[215] Gerwick W.H., Fenical W. (1981). Ichthyotoxic and cytotoxic metabolites of the tropical brown alga *Stypopodium zonale* (Lamouroux) Papenfuss. J. Org. Chem..

[216] Areche Medina C., Benites J., Cornejo A., Ruiz L.M., García Beltrán O., Simirgiotis M.J., Sepúlveda B. (2015). Seco-Taondiol, an Unusual Meroterpenoid from the Chilean Seaweed Stypopodium flabelliforme and Its Gastroprotective Effect in Mouse Model. Mar. Drugs.

[217] Dethe D.H., Mahapatra S., Sau S.K. (2018). Enantioselective total synthesis and assignment of the absolute configuration of the meroterpenoid (+)-taondiol. Org. Lett..

[218] Jiao W.-H., Xu T.-T., Yu H.-B., Chen G.-D., Huang X.-J., Yang F., Li Y.-S., Han B.-N., Liu X.-Y., Lin H.-W. (2014). Dysideanones A–C, unusual sesquiterpene quinones from the south china sea sponge dysidea avara. J. Nat. Prod..

[219] Jiao W.-H., Shi G.-H., Xu T.-T., Chen G.-D., Gu B.-B., Wang Z., Peng S., Wang S.-P., Li J., Han B.-N. (2016). Dysiherbols A–C and dysideanone E, cytotoxic and NF-κB inhibitory tetracyclic meroterpenes from a *Dysidea* sp. marine sponge. J. Nat. Prod..

[220] Chong C., Zhang Q., Ke J., Zhang H., Yang X., Wang B., Ding W., Lu Z. (2021). Total Synthesis of Anti-Cancer Meroterpenoids Dysideanone B and Dysiherbol A and Structural Reassignment of Dysiherbol A. Angew. Chem. Int. Ed..

